# Monocytic Subsets Impact Cerebral Cortex and Cognition: Differences Between Healthy Subjects and Patients With First-Episode Schizophrenia

**DOI:** 10.3389/fimmu.2022.900284

**Published:** 2022-07-11

**Authors:** Song Chen, Fengmei Fan, Fang-Ling Xuan, Ling Yan, Meihong Xiu, Hongzhen Fan, Yimin Cui, Ping Zhang, Ting Yu, Fude Yang, Baopeng Tian, L. Elliot Hong, Yunlong Tan, Li Tian

**Affiliations:** ^1^ Peking University HuiLongGuan Clinical Medical School, Beijing Huilongguan Hospital, Beijing, China; ^2^ Institute of Biomedicine and Translational Medicine, Department of Physiology, Faculty of Medicine, University of Tartu, Tartu, Estonia; ^3^ Department of Pharmacy, Peking University First Hospital, Beijing, China; ^4^ Maryland Psychiatric Research Center, Department of Psychiatry, University of Maryland School of Medicine, Baltimore, MD, United States

**Keywords:** monocytes, immune genes, first-episode schizophrenia, cerebral cortex, cognition

## Abstract

Monocytes are a highly heterogeneous population subcategorized into classical, intermediate and nonclassical subsets. How monocytes and their subsets may shape brain structures and functions in schizophrenia remains unclear. The primary goal of this cross-sectional study was to investigate monocytic subsets and their specific signature genes in regulation of cerebral cortical thickness and cognitive functions in first-episode schizophrenia (FES) patients. Whole-blood RNA sequencing of 128 FES patients and 111 healthy controls (HCs) were conducted and monocyte-specific differentially expressed genes were further analyzed. The MATRICS Consensus Cognitive Battery (MCCB) test, cortical neuroimaging and flow cytometric staining of peripheral blood monocytic subsets were performed among the participants. Significant changes in expressions of 54 monocytic signature genes were found in patients, especially for intermediate and nonclassical monocytic subsets with the most outstanding alterations being downregulated S100 Calcium Binding Protein A (*S100A*) and upregulated Interferon Induced Transmembrane Protein (*IFITM*) family members, respectively. Meanwhile, percentage of blood nonclassical monocytes was decreased in patients. Cortical thicknesses and MCCB performance were expectantly reduced and weaker intra-relationships among monocytic signature genes and cortices, respectively, were noted in patients compared to HCs. Monocytic genes were negatively associated with both cortical thicknesses and cognition in HCs, which was interestingly weakened or even reversed in patients, with nonclassical monocytic genes showing the greatest statistical significance. This study reveals that while monocytes may have negative effects on brain structure and cognition, the ameliorated phenomenon observed in schizophrenia may reflect an (mal)adaptive change of monocytes at early stage of the disorder.

## Introduction

The immune system has been found to interact with the central nervous system (CNS) in various cognitive and behavioral functions, the dysregulation of which contributes to chronic low-grade inflammation in schizophrenia (1). We recently observed that allostatic load, a composite index (including immune factors) of stress maladaptation, negatively contributed to cortical and cognitive deficits (2) and was associated with enlargement of the choroid plexus – an important cerebral portal for circulatory immune cells – in treatment-naïve first-episode schizophrenia (FES) patients (3).

Circulating human monocytes are notably a heterogeneous population, which is reflected for example by the relative expression levels of CD14 and the low-affinity Fcγ-III receptor (CD16) that namely divide monocytes into the classical (CD14++CD16-), intermediate (CD14++CD16+) and nonclassical subsets (CD14+CD16++). Classical, intermediate, and nonclassical monocytes account for ~85%, 5%, and 10% of circulating monocytes, respectively (4, 5). Each of these subsets is regarded as phenotypically distinct, carrying different signature genes and playing nonoverlapping roles in a myriad of chronic inflammatory and autoimmune conditions including neurological diseases (6–8). Classical monocytes have the highest phagocytic capacity and a predominant antimicrobial function (4). By contrast, nonclassical monocytes are generally viewed as anti-inflammatory and patrol along the vasculature, thereby playing a critical role in maintaining vascular homeostasis (8). Nevertheless, as a highly complex functional subset, nonclassical monocytes can also be pro-inflammatory and sensitive to immune senescence after being stimulated (9). Intermediate monocytes presumably represent a transitional stage between the classical and nonclassical monocytes, sharing some phenotypic and functional characteristics of both subsets (5), and are involved in antigen presentation to induce T-cell proliferation in infectious (4, 10) and inflammatory conditions (11, 12).

Limited studies produced some knowledge on monocytes in schizophrenia previously. For instance, a higher monocyte count (13, 14) and increased monocytic subcellular organelles including the nucleolus, mitochondria and lysosomes (15) were observed in schizophrenic patients compared to healthy controls (HCs). Monocytic transcriptomes of patients with schizophrenia had stronger inflammatory gene expression fingerprints (16). Studies including ours also found altered levels of toll-like receptors (TLR) and proinflammatory cytokine responses such as IL-1β in monocytes following stimulation with TLR ligands in schizophrenia (15, 17, 18).

However, imaging studies measuring microglial/myeloid activation in both living and post-mortem brains have yielded mixed results in schizophrenia in recent years, and some evidence even suggests a noninflammatory thereby protective phenotype of microglia in non-affective psychosis (1, 19, 20). Similar situation may also be true for monocytes. These call for more vigorous interrogations of these mononuclear phagocytic components to fully understand their functions.

No study has investigated monocytic subsets in psychiatric disorders and whether they may differently shape brain structures and functions in both normal and schizophrenic conditions so far. Hereby, we recruited FES patients and HCs and performed whole-blood RNA sequencing (RNAseq) along with flow cytometry, cognitive measurements and neuroimaging. Our primary goal was to investigate the inter-relationships of monocytic subsets and their specific transcriptomic profiles with cerebral cortical structures and cognitive functions in normal individuals and early illness schizophrenia patients with minimal antipsychotic exposure.

## Materials and Methods

### Ethical Approval and Participants

The present study used a cross-sectional research design. This study complied with the Declaration of Helsinki regarding an investigation in humans, and the study protocol was approved by the Medical Ethical Committee of Beijing Huilongguan Hospital. All participants gave written informed consent before the initiation of study procedures.

FES patients (n = 128) were enrolled in Beijing Huilongguan Hospital from 2016 to 2018, and the inclusion criteria were: 1) diagnosis of schizophrenia based on the Structured Clinical Interview for DSM-IV (SCID), which was administered by two experienced psychiatrists; 2) Han nationality and aged 18-55 years old; 3) illness duration ≤36 months; 4) education equal or greater than 8 years; 5) right handedness, and physically healthy in the past; 6) cumulative exposure to psychotropic drugs ≤ 14 days; 7) receiving no immunomodulators, immune-suppressive or anti-inflammatory agents in the past 6 months; 8) no substance and alcohol abuse/dependence.

Healthy controls (n = 111) were recruited from the local community simultaneously. Complete medical histories of HCs were collected, and physical examinations were conducted for all participants to identify those with chronic medical or psychiatric conditions. Potential control participants who had previously been diagnosed with an Axis I psychiatric disorder based on SCID criteria or had experienced substance abuse or dependence within the previous six months, and those who had a history of autoimmune disorders or other significant medical conditions or received anti-inflammatory medications were excluded. The other general criteria were the same as to FES patients.

### Blood Sample Collection, RNAseq and Data Analysis

Whole blood (5 ml) was collected between 7 and 9 AM after overnight fasting using PAXgene™ blood RNA tubes (Applied Biosystems, USA). Tubes were shaken vigorously for at least 10 seconds after sampling and immediately stored at -80°C. Total RNAs were extracted using Mag-MAX™ RNA Isolation Kit (Applied Biosystems, USA) by following the manufacturers’ instructions. RNAs were quantified and assessed for purity by optical density ratios of 260nm/280nm and 260nm/230nm using NanoDrop spectrophotometry, and samples (1 µg) were immediately shipped on dry ice to the laboratory of the Beijing Genomics Institute (BGI), China for sequencing on the BGIseq-500 platform. Quality controls (QCs) on RNA samples (RIN/RQN ≥ 7.0, 28S/18S ≥ 1.0) were confirmed by BGI, followed by globin mRNA removal and cDNA library construction. Clean data of at least 4Gb (20M clean reads) per sample were collected.

After QC of fastq files, an mRNA-seq count table was obtained from bam files. Gene expression analysis was done on NetworkAnalyst platform using DESeq2 (21). Counts with variance percentile rank < 15% and counts < 4 were filtered out, transformed and normalized to Log2 reads per million values. Fold changes (Log2FC) were calculated for differentially expressed genes (DEGs) between FES patients and HCs, with significance of *p* < 0.05 adjusted by Benjamini-Hochberg (BH)’s false discovery rate (FDR).

Monocyte-specific DEGs according to the relevant literatures of monocytic transcriptomic profiling (7, 22–25) were further selected for subsequent analyses of gene ontology (GO)-based biological processes (BP), Kyoto Encyclopedia of Genes and Genomes (KEGG), and protein-protein interaction (PPI) by the Database for Annotation, Visualization and Integrated Discovery (DAVID) and Search Tool for the Retrieval of Interacting Genes (STRING), respectively, with FDR < 0.05 as the cut‐off for significantly enriched GO terms and KEGG pathways. PPI network was set at a high confidence threshold of 0.7 and clustered with k-means method. Plots of DEGs were made using R, GraphPad, and online Morpheus.

### Blood Sampling and Flow Cytometric Staining of Peripheral Blood Monocytes

Five (5) ml of fresh heparin lithium-anticoagulated peripheral blood samples were collected from 29 FES patients and 27 HC subjects of the above participants after overnight fast, and processed within half an hour for fluorescent staining of cell surface receptors as described previously (18). The fluorochrome-conjugated antibodies used in this study were 10 μl FITC-labeled mouse anti-human CD14 (Clone M5E2; Catalog Number 555397; BD Biosciences) and 3 μl PerCP-Cy™5.5-labeled mouse anti-human CD16 (Clone B73.1; Catalog Number 565421; BD Biosciences). The percentages of classical (CD14++CD16-), intermediate (CD14++CD16+) and non-classical (CD14+CD16++) subsets among the total monocyte population were determined based on corresponding gatings. Single cells were filtered through cell strainers, carefully suspended, and immediately acquired by a BD FACSCalibur flow cytometer and the analyses were performed with FlowJo V10 software, as shown in **Figure 2A**.

### Cognitive Assessments

The MATRICS™ Consensus Cognitive Battery (MCCB) test was applied to assess the cognitive functioning for the subjects. It consists of ten tests encompassing seven cognitive domains, and domain scores as well as a composite score were computed using the MCCB scoring program. The clinical validity and reliability of Chinese version of MCCB had been previously established in both healthy volunteers and schizophrenia patients (26).

### Magnetic Resonance Image (MRI) Acquisition and Processing

Structural T1-weighted MRI brain images were acquired with a 3.0-T Prisma scanner (Siemens, Germany) and a 64-channel head coil at the Brain Imaging Center of Beijing Huilongguan Hospital. We used a sagittal 3D magnetization-prepared rapid acquisition gradient echo (MP-RAGE) sequence to get the T1 structural images. The acquisition parameters were as follows: repetition time (TR) =2,530 ms, echo time (TE) =2.98 ms, field-of-view (FOV) =256×224 mm, flip angle (FA) =7°, matrix size =256×224, thickness/gap =1/0 mm, and inversion time (TI) =1100 ms. Cortical thickness was estimated using FreeSurfer v5.3 (http://surfer.nmr.mgh.harvard.edu/) following the protocol of the Enhancing Neuro Imaging Genetics Through Meta Analysis (ENIGMA) Consortium (http://enigma.ini.usc.edu/). Images were visually checked for quality to make sure that cortical regions were properly segmented and labeled. Thirty-four distinct gyrally-defined regions per hemisphere were extracted according to the Desikan-Killiany atlas (27). All cortical regions were averaged for the left and right hemispheres (28). Brain images were obtained within 7 days after signing informed consent form, and 60 FES patients and 54 HCs from the total cohort, including the subjects in the aforementioned flow cytometric experiment, completed the MRI scan.

### Statistical Methods

Statistical procedures were performed with the IBM SPSS Statistics 21.0. Normality of distribution was confirmed through the Shapiro-Wilkinson test. Demographic and clinical data were compared between the two groups by Student’s *t*-test, Mann-Whitney *U* test, or Chi-Square test. Pearson and Spearman correlation were used to determine associations between variables where appropriate. Age and sex were used as covariates for ANCOVA as well as partial correlation analyses. For analysis involving cognitive function, the covariates also included the education years. Partial correlations were employed to test the relationships among normalized RNAseq counts of monocytic DEGs, cortical thicknesses, and cognitions, and all partial correlation coefficients were transformed into Fisher Z_r_ values. The Z_r_ values within the monocytic DEGs and brain regions, as well as among the three modules of genes, cortices and cognitions were compared between the FES and HC groups by paired *t*-test, respectively. Two-tailed *p* values were corrected for multiple testing with the FDR < 0.05 considered as significance. The PROCESS v3.3 for SPSS was used to perform the mediation analyses, with age, gender, and education years as covariates. The 95% confidence intervals (CIs) of direct and indirect effects were obtained from 5000 bootstrap samples. Statistically significant mediation effect was determined if the CI of indirect path did not contain zero.

## Results

### Demographic and Clinical Data

A total of 128 FES patients and 111 healthy subjects were recruited according to the inclusion and exclusion criteria. No difference was observed between the groups for age, sex and smoking status (all *p* > 0.05) (**Table 1**). Years of education was significantly less in FES patients than that in HCs (*p* < 0.01) (**Table 1**).

**Table 1:** Participant demographic and clinical characteristics.

Characteristics	FES (*n*=128)	HC (*n*=111)	Z/X^2^	*p*-value
Age (years)	30.64 ± 9.53	32.99 ± 9.57	-1.937	0.053
Males/females	55/73	56/55	1.338	0.247
Education (years)	12.76 ± 3.41	13.79 ± 2.59	-2.612	0.009^**^
Smoker/Non-smoker	18/110	23/88	1.854	0.173
Illness duration (months)	12.28 ± 12.25	NA	NA	NA
PANSS total	76.45 ± 12.94	NA	NA	NA
P subscore	22.21 ± 5.10	NA	NA	NA
N subscore	17.41 ± 6.22	NA	NA	NA
G subscore	36.85 ± 7.17	NA	NA	NA

PANSS Positive and Negative Syndrome Scale. ^**^p < 0.01. The bold values indicate statistically significant differences. NA, Not Applicable.

In the patient group, 18 patients were drug-naïve, and 110 patients had been exposed to antipsychotics for 1-14 days (median 4 days) at the time of blood drawing. The majority of patients were taking risperidone (n = 48), aripiprazole (n = 16), risperidone combined with haloperidol injection (n = 15), and olanzapine (n = 14) (**Supplementary Table 1**).

### Peripheral Blood RNAseq Transcriptomic Profiling for Monocytic Genes

**Figure 1:**
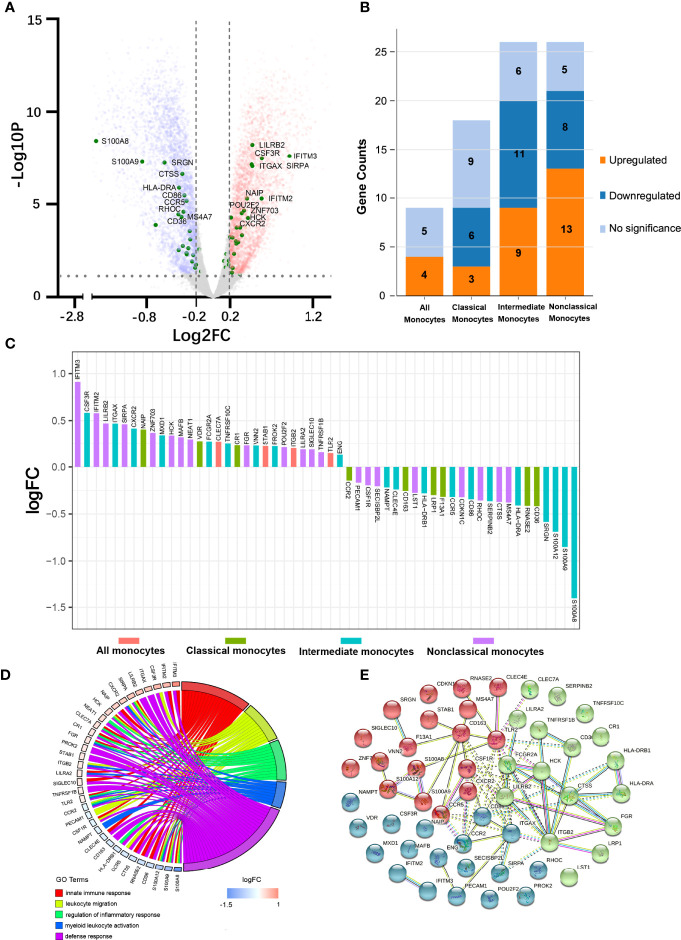
Peripheral blood RNAseq transcriptomic profiling shows differentially expressed monocytic genes between FES patients and HCs. **(A)** Volcano plot indicates the DEGs (FDR < 0.05, Log2FC > |0.2|), depicted as upregulated (red) or downregulated (blue), in FES patients compared to HCs. Fifty-four monocyte subset-specific DEGs are highlighted (green) and those with -Log10*P* > 3 are nominated. **(B, C)** These DEGs (FDR < 0.05) were especially enriched in intermediate and nonclassical monocytes. **(D)** Chord plot shows most of the 54 DEGs in association with the top nonoverlapping GOBP subontology terms, as analyzed in DAVID. Genes are ordered according to the observed Log2FC and linked to their assigned terms *via* colored ribbons. **(E)** PPI analysis shows the interactions of DEGs set at a high confidence threshold of 0.7 and clustered into 3 color-coded sets with k-means = 3. Line between nodes features the type/strength of an interaction according to annotations in String. See also **Supplementary Tables 2**, **3**.

We first studied the molecular signatures of circulating leukocytes and identified 9062 DEGs (FDR < 0.05) between FES patients and HCs, including 4479 upregulated and 4583 downregulated genes (**Figure 1A**). To get a better insight into the gene expression changes among CD14/CD16-subsets of monocytes in schizophrenia, 79 subset-specific signature genes were chosen based on recent monocytic transcriptomic profiling works (7, 22–25), among which 54 were found to have FDR < 0.05 in our RNAseq datasets (**Figures 1A-C**; **Supplementary Table 2**).

These 54 DEGs were enriched in different monocytic subtypes, respectively 4 (7.4%), 9 (16.7%), 20 (37.0%), and 21 (38.9%) belonging to all monocytes, classical, intermediate and nonclassical monocytes (**Figure 1B**). In addition, among these subclasses, the majority (6 out of 9, i.e. 66.7%) of DEGs belonging to the classical monocytes were downregulated and that (13 out 21, 61.9%) of DEGs belonging to nonclassical monocytes were upregulated, while the proportions of up- and down-regulated genes (45% *vs.* 55%) were more equal in intermediate monocytes (**Figure 1B**). Notably, among the DEGs with the most significantly changed expression (|Log2FC| > 0.5), S100 Calcium Binding Protein A family genes (*S100A8, S100A9* and *S100A12*, all downregulated) belonging to intermediate monocytes and Interferon Induced Transmembrane Protein family genes (*IFITM2* and *IFITM3*, both upregulated) belonging to nonclassical monocytes outstood (**Figure 1C**).

We next checked the functions of these 54 DEGs based on GOBP terms in DAVID. From the 18 annotated clusters with enrichment score ranged from 0.84 to 5.26 (FDR < 0.05, **Supplementary Table 3**), the most significant and nonoverlapping GOBP terms from each of the top 5 functional clusters were found to be involved in leukocyte migration, regulation of inflammatory response, myeloid leukocyte activation, innate immune response, and defense response, etc. (**Figure 1D**). Similarly, KEGG analysis revealed that the most significantly enriched pathways were infectious and autoimmune diseases (**Supplementary Table 3**). It is noteworthy that major histocompatibility complex, class II, DR alpha and beta 1 (*HLA-DRA/B1*) were observed in the majority of the KEGG enriched pathways.

PPI analysis of these monocytic DEGs further revealed a network containing 53 nodes and 75 edges, with PPI enrichment *p*-value < 1.0×10^-16^. Under the clustering criterium of k-means = 3, the network can be further grouped into the three interconnected clusters, in which genes such as the *S100A* family members, the *IFITM* family members, and the *CR1*-*ITG* family members are covered, respectively (**Figure 1E**).

### Comparisons on Abundancy of Blood Monocytic Subsets Between FES and HC

A subgroup consisted of 29 FES patients and 27 HCs, of whom 23 FES patients and 26 HCs were also included in the total cohort, was used to explore the distribution of monocytic subsets by flow cytometry. There were no significant differences in age, sex, education years, or smoking status between FES and HCs in this subgroup, and their clinical characteristics are displayed in **Supplementary Table 4**.

**Figure 2:**
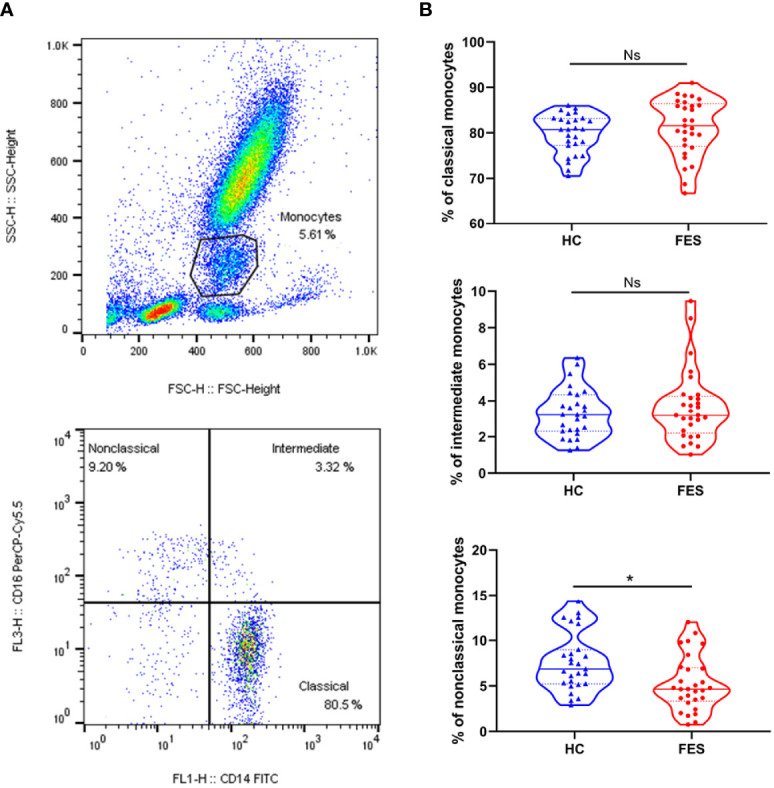
Nonclassical monocytes are decreased in the peripheral blood of FES patients. Whole blood sample was stained with monocytic markers FITC-labeled mouse anti-human CD14 and PerCP-Cy™5.5-labeled mouse anti-human CD16 and measured by flow cytometry. **(A)** Dotplots show gating strategy. Monocytes were first selected based on size (forward scatter height) and granularity (side scatter height), and then monocytic subsets were defined according to surface expressions of CD16 and CD14. The CD14++CD16- classical monocytes, CD14++CD16+ intermediate monocytes, and CD14+CD16++ nonclassical monocytes were quantitated, respectively. **(B)** Violin plots show the blood monocytic subsets in HCs and FES patients. Solid and dotted lines represent the median and upper/lower quartile values, respectively. Ns, Not significant. ^*^ FDR < 0.05. See also **Supplementary Table 5**.

Interestingly, FES patients had a decreased percentage of nonclassical monocytes compared to HCs after controlling for age and sex (5.27 ± 3.01% *vs.* 7.61 ± 3.21%, F = 8.736, FDR = 0.015) (**Figure 2B**). We found no group differences in the percentage of classical and intermediate monocytes. Besides, the two groups did not differ in neither the absolute count nor the percentage of total blood monocytes according to the routine clinical blood test. We also calculated the absolute counts of the three monocytic subsets derived from the total blood counts and their flow cytometric percentages, but the differences were insignificant, however (all *p* > 0.05, for nonclassical monocytes, *p* = 0.083, **Supplementary Table 5**).

### Comparisons on Brain Cortical Thickness and Cognition Between FES and HC

A subset of 60 FES patients and 54 HCs from the total cohort, including the participants in the above flow cytometric experiment, underwent further MRI and MCCB tests (two patients and two controls did not complete the MCCB assessment). There were no significant differences in age, sex, education years, or smoking status between FES and HCs in this subset, and their clinical characteristics are displayed in **Supplementary Table 6**.

**Figure 3:**
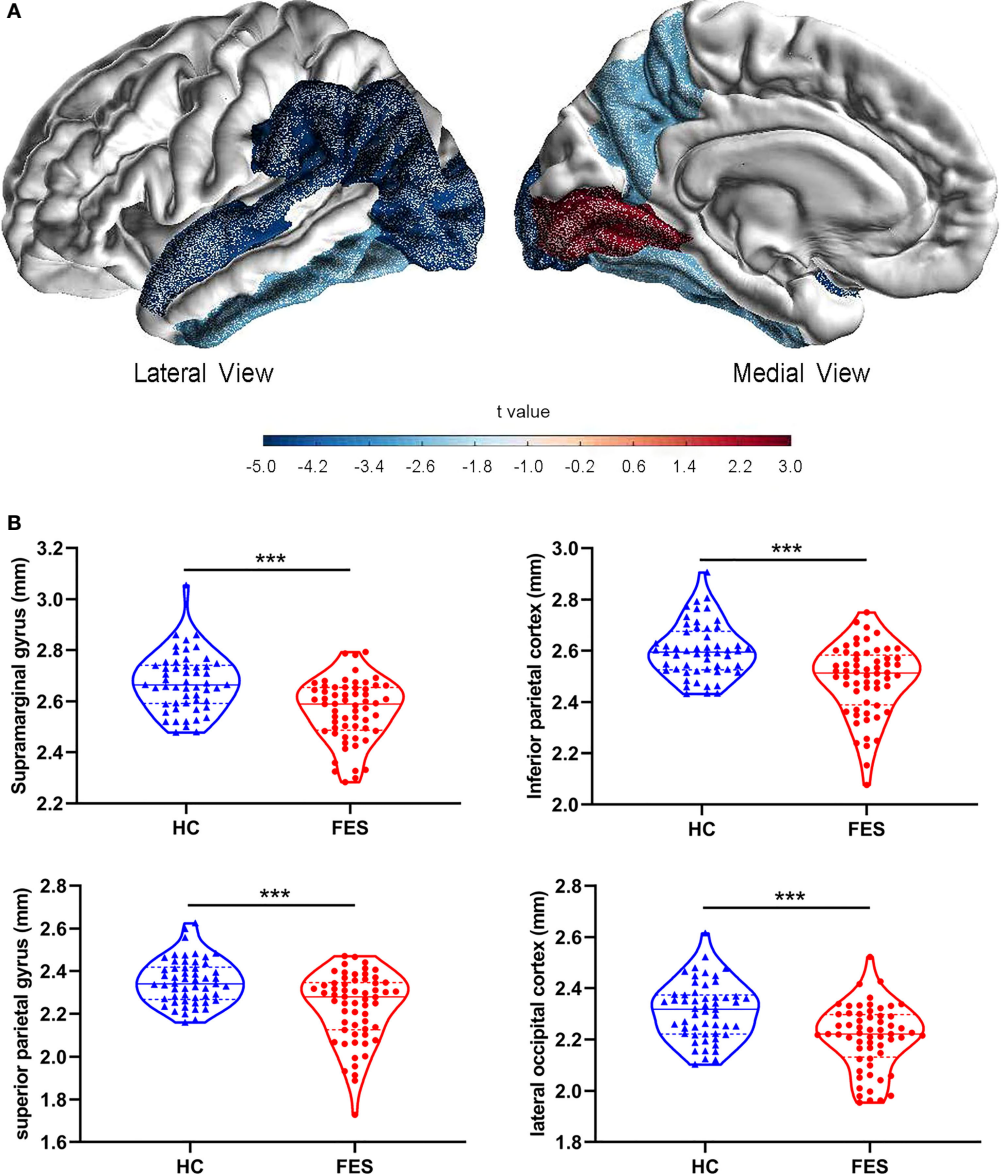
Brain cortical thicknesses are different between FES patients and HCs. **(A)** Thirty-four distinct gyrus-defined regions per hemisphere were extracted according to the Desikan-Killiany atlas and averaged for both hemispheres in HCs and FES patients. Exemplary MRI images show the cortical regions with significant group differences in thickness, with blue and red colors encoding cortical thinning and thickening in FES patients as compared to HCs, respectively, after FDR correction and controlling for age and sex. Color gradient is based on the statistical *t* values of group comparison. **(B)** Brain regions with the most significant reduction in cortical thickness of FES patients are shown, with median and upper or lower quartile drawn in violin plots, respectively. ^***^ FDR < 0.001. See also **Supplementary Table 7**.

On the structural imaging aspect, the whole-brain average cortical thickness was reduced in FES patients versus HCs after controlling for age and sex (2.54 ± 0.08 *vs*. 2.59 ± 0.09, F = 10.078, *p* = 1.946×10^-3^). As illustrated in exemplary MRI images in **Figure 3A**, among the 34 cortical regions defined by the DK atlas, 8 regions, i.e. supramarginal gyrus, inferior parietal cortex, superior parietal gyrus, lateral occipital cortex, inferior temporal gyrus, precuneus, fusiform gyrus, and superior temporal gyrus were all remarkably thinner in FES individuals, while their pericalcarine and lingual gyrus were statistically thicker than those of HCs (all FDR < 0.05) (**Figure 3B**; **Supplementary Table 7**).

As expected for MCCB test, FES patients also showed significantly lower total MCCB score and the seven domain subscores compared to HCs after controlling for age, sex, and education years (all FDR < 0.001) (**Table 2**).

**Table 2:** Comparison of the MCCB scores between first-episode schizophrenia patients and healthy controls.

MCCB domains^a^	FES (*n*=58)	HC (*n*=52)	F	*p*-value	FDR
Composite score	44.93± 9.30	58.69 ± 6.98	76.397	4.047×10^-14^	NA
Processing speed	44.32 ± 11.05	57.50 ± 8.73	42.368	2.383×10^-9^	8.341×10^-9***^
Attention/vigilance	40.50 ± 11.91	57.17 ± 10.83	49.714	1.707×10^-10^	1.195×10^-9***^
Working memory	46.00 ± 10.94	57.55 ± 7.17	37.923	1.284×10^-8^	2.996×10^-8***^
Verbal learning	48.72 ± 10.98	57.37 ± 7.78	17.237	6.572×10^-5^	7.667×10^-5***^
Visual learning	45.53 ± 9.78	55.02 ± 7.22	28.969	4.258×10^-7^	5.961×10^-7***^
Reasoning/problem solving	46.98 ± 10.33	56.63 ± 7.40	32.037	1.238×10^-7^	2.167×10^-7***^
Social cognition	46.32 ± 11.16	54.46 ± 10.08	12.409	6.253×10^-4^	6.253×10^-4***^

a Analysis of covariance with age, sex and education years as covariates. MCCB MATRICS™ Consensus Cognitive Battery. ^***^FDR < 0.001. The bold values indicate statistically significant differences. NA, Not Applicable.

### Associations Among Monocytic Subset Signature Genes, Cortical Thickness, and Cognition

**Figure 4:**
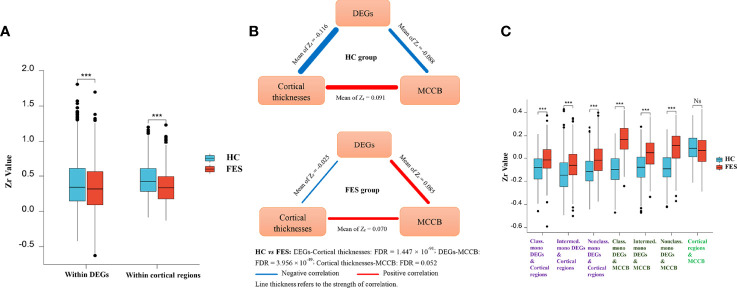
Monocytic DEG mRNAs, cortical thicknesses, and cognition show differential correlations among each other in FES patients and HCs. Partial correlational analyses of the 54 monocytic DEGs normalized RNAseq counts, the averaged thicknesses of the bi-hemispheric 34 brain cortical regions, and the MCCB subscores, controlled for age, sex, and education years in HCs and FES patients, respectively. All partial correlation coefficients were transformed into Fisher Z_r_. The intra-associations within the monocytic DEGs module and the brain regions module **(A,** Mean ± SD shown, ^***^ *p* < 0.001**)**, as well as inter-relationships among the three modules **(B)**, including DEGs refined to different monocytic subsets **(C,** Mean ± SD shown, ^***^ FDR < 0.001, Ns: Not significant**)**, were compared between the two groups. HVLT: Hopkins verbal learning test; BVMT: brief visual-spatial memory test; CPT: continuous performance test; SOPT: speed of processing test; WMT: working memory test; EIT: emotion intelligence test; MAZET: mazes test. See also **Supplementary Figures 1A, B**.

To explore the relationships of monocytic DEGs with brain structure and cognition, we performed partial correlational analyses of the three modules, e.g., the 54 DEGs RNAseq counts, the thicknesses of the 34 brain cortical regions, and the MCCB subscores, controlled for age, sex, and education years in the FES and HC groups, respectively (**Figure 4**; **Supplementary Figure 1**). Correlational matrices of these three modules showed that the main differences between the two groups included two aspects.

Firstly, the internal positive correlations among the 54 DEGs inside the gene module and among the 34 brain regions inside the cortex module were both significantly attenuated in the FES group as compared to those in the HCs (Z_r_-mean within DEGs: 0.388 *vs* 0.335, *t* = -8.319, *p* = 2.052 × 10^-16^; Z_r_-mean within brain regions: 0.449 *vs* 0.346, *t* = -10.576, *p* = 5.886 × 10^-24^) (**Figure 4A**; **Supplementary Figures 1A, B**). As a whole profile of the inter-relationships among the three modules, in the HC group, mono-DEGs counts were in strikingly inverse correlations with both cortical thicknesses and MCCB subscores, which were positively associated with each other (**Figure 4B**). By contrast, in the FES group, such negative correlation of the mono-DEGs with the cortical thicknesses was markedly weakened (Z_r_-mean: -0.116 *vs* -0.025, *t* = 21.555, FDR = 1.447 × 10^-91^), and even reversed to a positive correlation with the cognitive scores (Z_r_-mean: -0.088 *vs* 0.085, *t* = 17.188, FDR = 3.956 × 10^-49^), respectively (**Figure 4B**). The positive correlation between the cortical thicknesses and the cognitive scores was also trendily weakened in FES patients (Z_r_-mean: 0.091 *vs* 0.070, *t* = -1.955, FDR = 0.052) (**Figure 4B**, **Supplementary Figures 1A, B**).

Secondly, when comparing the subset-specific mono-DEGs, we found that the negative correlations between them and the cortical regions were also remarkably weakened in the FES group, especially for the nonclassical mono-DEGs, showing the greatest statistical significance (Classical mono-DEGs: Z_r_-mean: -0.089 *vs* -0.005, *t* = 8.383, FDR = 2.697 × 10^-15^; intermediate mono-DEGs: Z_r_-mean: -0.134 *vs* -0.054, *t* = 11.737, FDR = 1.511 × 10^-28^; nonclassical mono-DEGs: Z_r_-mean: -0.111 *vs* -0.010, *t* = 15.236, FDR = 9.638 × 10^-45^) (**Figure 4C**).

Furthermore, compared to the HC group, correlations between the subset-specific mono-DEGs and the MCCB subscores were reversed in the FES group, and likewise, with the nonclassical mono-DEGs showing the greatest statistical significance (Classical mono-DEGs: Z_r_-mean: -0.095 *vs* 0.161, *t* = 10.949, FDR = 6.997 × 10^-16^; intermediate mono-DEGs: Z_r_-mean: -0.081 *vs* 0.042, *t* = 7.939, FDR = 7.195 × 10^-13^; nonclassical mono-DEGs: Z_r_-mean: -0.081 *vs* 0.086, *t* = 10.114, FDR = 3.482 × 10^-18^) (**Figure 4C**).

**Figure 5:**
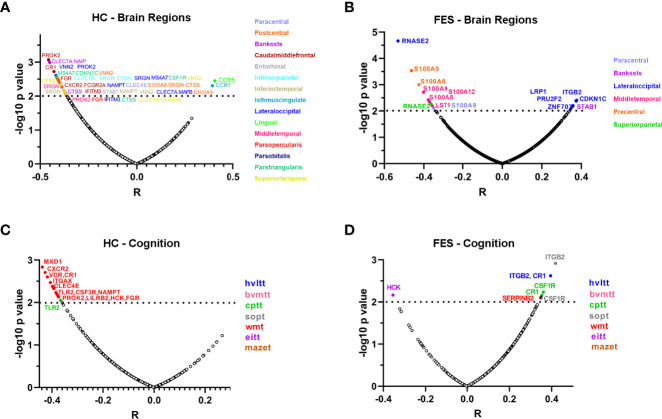
Correlations of individual monocytic DEG mRNAs with cortical thicknesses and MCCB scores highlight more extensive negative features in HCs compared to FES patients. **(A-D)** Partial correlational analyses of the mRNA levels of 54 monocytic DEGs with the averaged thicknesses of bi-hemispheric 34 cortical regions, after controlling age, sex, and education years, in HC **(A)** and FES **(B)** groups, respectively; and with the 7 MCCB subscale scores in HC **(C)** and FES **(D)** groups, respectively. The abscissa represents partial correlation coefficient. The significant level was set at *p* < 0.01. Among all the correlations, only the inverse relationship between the classical monocytic gene *RNASE2* and lateral occipital cortical thickness in FES patients passed the FDR (54×34) correction. HVLT, Hopkins verbal learning test; BVMT, brief visual-spatial memory test; CPT, continuous performance test; SOPT, speed of processing test; WMT, working memory test; EIT, emotion intelligence test; MAZET, mazes test.

Next, we checked the detailed correlations between DEGs and each cortical region, controlling for age, sex, and education years and setting the significant level at *p* < 0.01, as visualized in volcano plots (**Figure 5**). In the HC group, numerous negative correlations appeared, with the top outstanding ones coming from genes that were all upregulated in FES patients compared to HCs (**Figure 1C**), namely a pan-monocytic gene C-Type Lectin Domain Containing 7A (*CLEC7A*), a classical monocytic gene NLR family Apoptosis Inhibitory Protein (*NAIP*) and an intermediate monocytic gene Prokineticin 2 (*PROK2*) (**Figure 5A**).

In the FES group, the most highly significant correlation came from a classical monocytic gene Ribonuclease A Family Member 2 (*RNASE2*), which was downregulated in FES patients compared to HCs (Log2FC = -0.41, FDR = 0.003, **Supplementary Table 2**) and was inversely correlated with the lateral occipital cortical thickness (**Figure 5B**). Besides, the intermediate monocytic genes of the S100A family members were inversely associated with the thicknesses of the precentral, middle temporal and paracentral cortices in the FES group (**Figure 5B**). Among all the correlations in both groups, only the negative relationship between the *RNASE2* and the thickness of the lateral occipital cortex in the patient group survived multiple comparison correction for 54×34 tests (partial *r* = -0.531, FDR = 0.040).

We also explored whether the percentage of nonclassical monocytes was associated with the cortical thickness. The results showed that among FES patients, after adjustment for age, sex, and education years, the percentage of nonclassical monocytes was inversely correlated with the cortical thicknesses of seven anatomic regions, including the paracentral lobule, entorhinal cortex, fusiform gyrus, middle temporal gyrus, pars opercularis of inferior frontal gyrus, superior temporal gyrus, and temporal pole (partial *r* within the range of -0.5 ~ -0.7, all FDR < 0.05) (**Supplementary Figure 2A**). No significant correlations between the percentage of nonclassical monocytes and the thicknesses of cortical regions in the HC group (**Supplementary Figure 2B**) and between classical or intermediate monocytes and cortical regions in both groups were detected (all FDR > 0.05).

Additionally, negative associations between expressions of a variety of monocytic genes and MCCB subscores in the HC group existed (**Figure 5C**), among which some were reverted to positive relationships in the FES group, especially the complement C3b/C4b receptor 1 (*CR1*), integrin family (*ITG*), and colony stimulating factor receptor family (*CSFR*) genes that belong to classical-intermediate monocytes (**Figure 5D**), but none of these correlations passed the FDR correction. However, there were no significant correlations between the percentage of nonclassical monocytes and all the MCCB domains in both groups even at nominal levels (all *p* > 0.05).

### Mediation Effect of the Cortical Thickness on the Association of Monocytic Gene and Cognition

**Figure 6:**
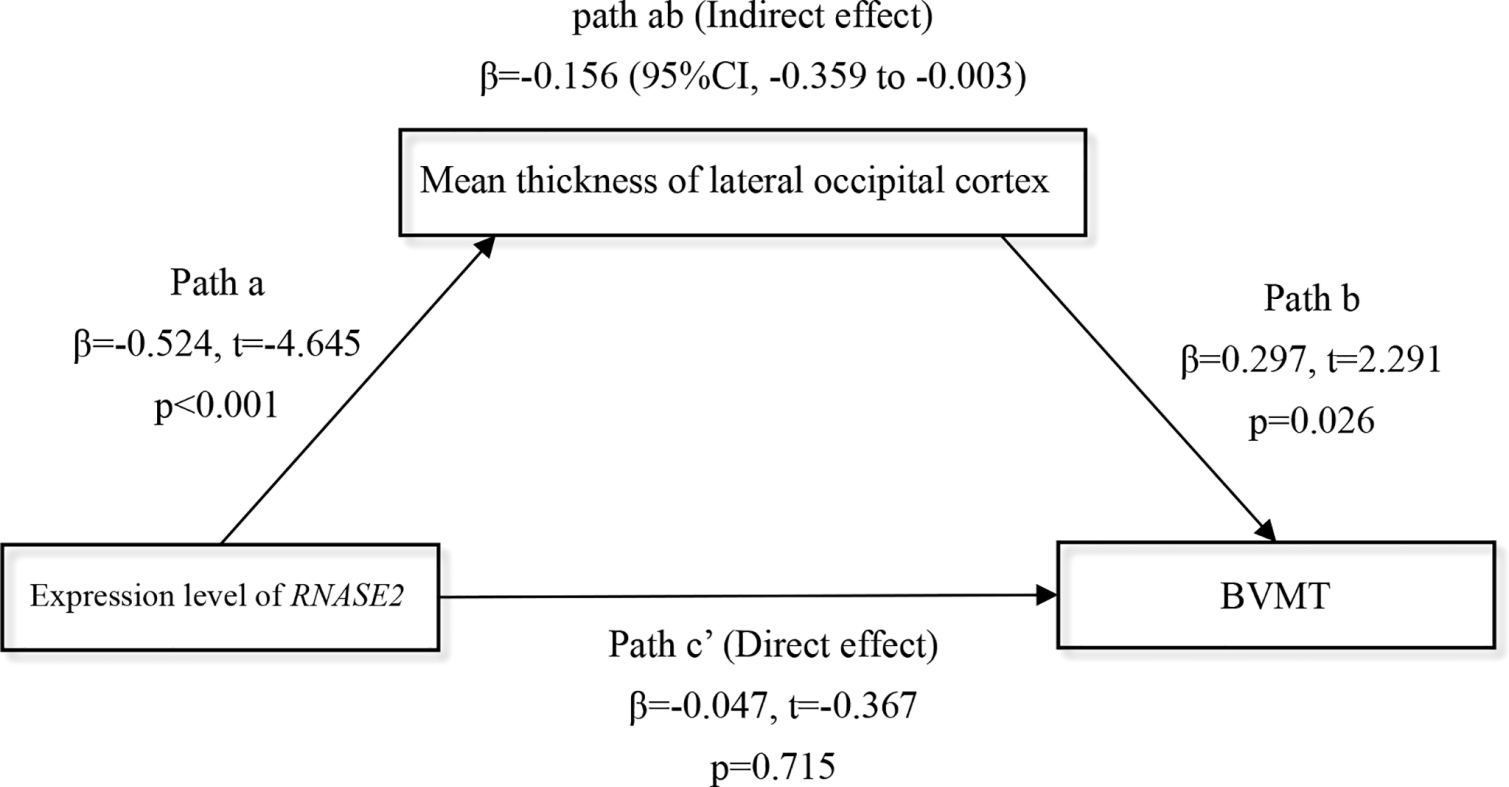
Full mediation effect of the lateral occipital cortex between the gene *RNASE2* and visual memory. Independent variable = *RNASE2* expression level; Dependent variable = brief visual-spatial memory test (BVMT) score; Mediator = lateral occipital cortex. Age, sex, and education years were included as covariates.

Finally, considering the overt relationship between the gene *RNASE2* and the lateral occipital cortical thickness in the FES group (**Figure 5B**), we further explored whether the lateral occipital cortex may mediate the *RNASE2*-cognition relationship in patients. Because the thickness of the lateral occipital cortex was only associated with the brief visual-spatial memory test (BVMT) of MCCB at nominal level (partial *r* = 0.370, nominal *p* = 0.005), BVMT score was used as the dependent variable in the mediation analysis. Introducing the lateral occipital cortical thickness as a mediator, the indirect path (path ab) from expression level of *RNASE2* to BVMT was significant (β = -0.156, 95% CI, -0.359 to -0.003), while the direct effect (path c’) between them was insignificant (β = -0.047, t = -0.367, *p* = 0.715), which implied that the effect of *RNASE2* on BVMT score was fully mediated by the lateral occipital cortex (**Figure 6**).

## Discussion

Previous studies have not considered monocytes as a highly heterogeneous population that may have complicated roles in schizophrenia. Our major findings are : (1) Nonclassical monocytes were decreased and monocyte-related transcriptomic profiles showed significant change in FES, especially for intermediate and nonclassical monocytic subsets, with the most outstanding alterations being downregulated *S100A* and upregulated *IFITM* family members belonging to the intermediate and nonclassical monocytes, respectively; (2) Inverse inter-relationships of monocytic DEGs with cortical thicknesses and cognition existed in HCs, which was ameliorated in FES patients; meanwhile, the percentage of nonclassical monocytes was negatively associated with the thicknesses of multiple brain cortical regions in FES patients; (3) The lateral occipital cortex fully mediated the negative effect of a classical monocytic gene *RNASE2*, encoding an eosinophil-derived neurotoxin, on visual learning and memory in FES patients. An overall impression on these results is that whereas monocytic subsets and their genes may be detrimental for brain and cognition in healthy people, they may develop an (mal)adaptive inflammaging-related mechanism while trying to alleviate brain and cognitive deficits in FES, as elaborated below. Our results also do not fully support the stereotypical view that monocytes are all detrimental for brain and cognitive deficits in schizophrenia, especially in the early stage of the disease.

### Decreased Proportion of Nonclassical Monocytes and Upregulated Nonclassical Monocytic DEGs in Schizophrenia

We studied peripheral blood by flow cytometry and RNAseq to profile monocytic functions in FES, providing the first evidence to our knowledge that altered monocytic functions may be implicated in the pathogenesis at early stage of schizophrenia. Recent transcriptomic studies using blood, postmortem brains, and patient-derived cerebral organoids generated from induced pluripotent stem cells have also demonstrated dysregulation of inflammation and immune function in FES (29–31). To scrutinize specific monocytic functions with higher detail, we categorized the DEGs into different monocytic subtypes.

Decreased proportion of nonclassical monocytes along with upregulated signature genes in FES patients are the most interesting findings in the present study, although the exact underlying mechanism awaits to be revealed. We found that more DEGs fell into the intermediate and nonclassical monocytes compared to the classical subset in patients, suggesting that biological functions of these two subpopulations may be more affected. We speculate that the aberrant expressions of the candidate intermediate and nonclassical mono-DEGs and the looser intra-correlations among these monocytic DEGs in patients may be due to maladapted monocytic functions in FES patients, as discussed below.

Nonclassical monocytes play an important role in maintaining vascular homeostasis (8) and studies have demonstrated that patients with schizophrenia had vascular endothelial pathologies due to chronic low-grade systemic inflammation (32, 33). This possibly leads to nonclassical monocytes recruitment into the brain to repair parenchymal vascular endothelium, resulting in nonclassical monocytopenia in the peripheral blood along with altered mono-DEGs involved in leukocyte migration, as we found in FES patients. Similar observation was reported in patients with severe forms of lupus nephritis, showing that lower levels of nonclassical monocytes in the peripheral blood was accompanied by a higher degree of infiltrates of CD16+ cells in the glomerulus (33). Alternatively, it may be so that a progressive transition from classical to nonclassical monocytes due to immune senescence or inflammaging may be disturbed in patients, similarly as the coexistence of myeloid functional deficiencies and increased myelopoiesis during aging (34). Indeed, nonclassical monocytes were found in a pro-inflammatory state of senescence after stimulation by a recent study (9). This postulates a provocative concept that schizophrenia may be an inflammaging disorder, as corroborated by our finding that proinflammatory nonclassical monocytic genes such as the *IFITM* family were upregulated in FES patients.

We also found inverse correlations of the percentage of blood nonclassical monocytes with the cortical thickness especially in the temporal lobe of patients but not healthy participants, implying again that patients’ cortical dystrophy may require more infiltration of nonclassical monocytes for tissue repair. However, since the temporal lobe is a significant part of the limbic system that is known to regulate genesis and mobilization of myeloid cells (35), temporal cortical dystrophy may cause disturbance in this process in patients, causing nonclassical monocytes lingered in the blood of those patients with severe cortical dystrophy. It may also be possible that these negative correlations are attributed to the pro-inflammatory nature of the nonclassical monocytes, as mentioned above. These speculative accounts deserve further investigation.

Recent genetic research has highlighted CpG epigenetic changes in inflammatory genes in contribution to psychotic conversion in young adults (36). Interestingly, among the DEGs belonging to classical monocytes, most were downregulated in our FES patients. Likewise, in intermediate monocytes, downregulations of the myeloid alarmin-related *S100A* genes (*S100A8*, *S100A9* and *S100A12*) in patients were the most notable changes. *S100A* family acts as damage-associated molecular patterns (DAMPs) on pattern recognition receptors, such as RAGE and TLR4 receptors, which are important for phagocytosis, cytokine release, cell adhesion and migration (37). Previous studies have reported increased *S100A8*/*9*/*12* gene expression in peripheral blood cells, prefrontal cortex, and hippocampus of individuals with schizophrenia (30, 38, 39). The inconsistency with our results is possibly due to chronicity of the disease and antipsychotic medication in those studies.

As mentioned above, *IFITM* family members belonging to nonclassical monocytes were upregulated in FES patients, which is corroborated by earlier studies of the blood and postmortem brains of patients with schizophrenia, especially in the prefrontal cortex, hippocampus and cortical blood vessels, independent of antipsychotic use (1, 30, 40–42). IFITM2/3 proteins, which are mainly localized to late endosomal/lysosomal membranes, have been shown to restrict a broad range of viral entry and replication, such as influenza A virus and cytomegalovirus (43, 44), while Uranova et al. (15) found that the area and number of lysosomes of monocytes were significantly increased in schizophrenia patients as compared to HCs. Together with our findings on *IFITM2/3* genes, it is plausible that dormant or reminiscent viral infection may be associated with pathogenesis in FES, a hypothesis that has been persisted for decades and supported by epidemiological evidence on viral infection pandemics or perinatal immune activation models (45–47).

Taken together, our results highlight that not only global monocytic functions but also their subtypes may be more specifically affected in FES patients, which should not be neglected in this field of research.

### Associations Among Monocytic Subset DEGs With Cortical Thickness and Cognition

Our further finding was that FES patients exhibited widespread cortical thinning, primarily in the parietal, occipital and temporal cortices, in agreement with previous studies in FES patients, suggesting that extensive cortical gray matter loss has taken place in the early phase of illness. Cortical thickness reduction, which reflects neuronal/glial dystrophy and/or loss of synapses, is a consistent structural MRI finding in schizophrenia by others and us (2, 48, 49). However, unlike prior studies, we did not detect differences in the frontal and cingulate regions, possibly because we averaged the left and right hemispheres in our study.

Little is known about monocytic effect on the schizophrenic brain currently, but research findings in other fields may be enlightening. A recent study reported that higher plasma monocyte activation markers sCD14 and sCD163 were associated with smaller frontal and temporal cortical volumes among women with HIV (50). Moreover, chemokine receptor CCR2 on intermediate monocytes was correlated with disturbances of neuro-metabolites in the right and left caudate nucleus, contributing to HIV-associated neurocognitive disorders (51). Another related important research field is Alzheimer’s disease. Recently, disease-associated microglial subset implied in amyloidosis and cognitive impairment have been depicted in Alzheimer’s disease patients through single-cell RNAseq (52, 53). Several highly significant monocytic DEGs found in our current study, such as *CCR5*, *CLEC7A*, *CTSS*, *IFITM3*, and *ITGAX*, are associated with such microglial subtype. Also, a relationship between monocyte-derived inflammation and hippocampal atrophy as well as cognitive decline in Alzheimer’s disease had been reported (54).

Intriguingly, with a data-driven approach here, we detected that the negative inter-relationships between monocytic DEGs and cortical thicknesses as well as cognition were weakened in schizophrenia patients as compared to HCs. These indicate that firstly, monocytic subsets in the peripheral blood are negatively associated with brain and cognition in HCs generally; secondly, disruption of this detrimental role in FES patients may imply a functional alteration of some subsets of monocytes (e.g., inflammaging of nonclassical monocytes) with a compensatory mechanism of some other subsets of monocytes (e.g., down-regulation of genes in classical and intermediate monocytes) in the early stage of the disease.

Notably, among the DEGs associated with cortical thinning in both study groups, the classical monocytic gene *RNASE2* was the most striking one. The protein encoded by *RNASE2* is a cytotoxic protein that can induce proinflammatory cytokine production in monocytes/macrophages by acting as a DAMP (55). It causes severe damage to myelin and loss of neurons in the rabbit brain, an event known as the Gordon phenomenon (56). Possibly, a highly inverse association between *RNASE2* and lateral occipital cortical thickness in our FES patients may suggest that their thinner lateral occipital cortex may be particularly vulnerable to the neurotoxic effects of *RNASE2*, while the downregulation of *RNASE2* in patient group hints a self-protective response of host.

The current study has several limitations. Firstly, the cross-sectional design of this study limits our causal interpretations of results. Whether the observed alterations are a nature of compensatory effect in monocytes of FES patients remains unclear. Secondly, we performed RNAseq by using the peripheral whole blood samples, hence, the transcriptomic changes in our study are not solely restricted to monocyte subsets, which should be examined by other advanced approaches later. Moreover, the possible anti-inflammatory role of antipsychotic medication should be kept in mind, since the majority of patients had short-term (1-14 days, median 4 days) exposure to antipsychotics, with risperidone accounting for around 44% of them. Peripheral inflammatory cytokines were found to be affected by 11 weeks of risperidone treatment (57), highlighting the necessity to study drug-naïve patients. Thirdly, we did not validate the critical DEGs by quantitative-PCR, even though the current results could still offer a valuable preliminary transcriptional landscape of genes associated with monocyte subsets in the early course of schizophrenia. Finally, results from monocyte subtyping by flow cytometry should be interpreted with caution due to the small sample size in this research. A larger cohort of longitudinal design with information about early life exposure to immune challenge relevant to trained innate immunity (58), would be ideal.

## Conclusions

In summary, our findings demonstrate altered transcriptomic profiles and disproportionality of monocyte subpopulations at early stage of schizophrenia, with intermediate and nonclassical subsets coupled with *S100A* and *IFITM* family members worthy of special attention. Overall, monocytic subsets may negatively impact cortical thickness and cognition in HCs and less so in schizophrenia patients. Our results revealed a possible detrimental effect of monocytes on the brain and cognition, which was weakened by yet intriguing mechanism in schizophrenia. Given the high heterogeneity of monocytes, a more spectral than monotonic view on them should be kept in mind in the future.

## Data Availability Statement

The datasets presented in this study can be found in the European Nucleotide Archive (ENA) repository with the accession number: PRJEB53454.

## Ethics Statement

The studies involving human participants were reviewed and approved by medical ethical committee of Beijing Huilongguan Hospital. The patients/participants provided their written informed consent to participate in this study.

## Author Contributions

The study was initiated and directed by LT and YT. SC, MX, PZ, and TY were responsible of recruitment of study subjects and clinical assessments. FF performed the imaging data analysis. LT, YT, SC, FX, and LY performed statistical analysis and interpreted results. SC and LT wrote the first draft of the manuscript in consultation with YT. FX, LY, HF, YC, FY, BT, and LH were invited in evolving the ideas, analyzing data and editing the manuscript. All authors contributed to the article and approved the submitted version.

## Funding

This work was supported by the Academy of Finland research grant No. 273108 (LT), the Estonian Research Council-European Union Regional Developmental Fund Mobilitas Pluss Program No. MOBTT77 (LT), and the National Natural Science Foundation of China (81761128021, YT and 82001415, SC).

## Conflict of Interest

The authors declare that the research was conducted in the absence of any commercial or financial relationships that could be construed as a potential conflict of interest.

## Publisher’s Note

All claims expressed in this article are solely those of the authors and do not necessarily represent those of their affiliated organizations, or those of the publisher, the editors and the reviewers. Any product that may be evaluated in this article, or claim that may be made by its manufacturer, is not guaranteed or endorsed by the publisher.
