# Supplementary material for: Monocytic Subsets Impact Cerebral Cortex and Cognition: Differences Between Healthy Subjects and Patients With First-Episode Schizophrenia
Source: Front Immunol. 2022 Jul 11;13:900284. doi: 10.3389/fimmu.2022.900284 (PMC9309358; doi:10.3389/fimmu.2022.900284)
Supplement: Supplementary file 1 [file DataSheet_1.docx]

# Supplementary tables and table legends

## Supplementary Table 1. Distribution of antipsychotics at the time of enrollment in the total cohort

| **Antipsychotics** | **Number of patients (n=128)** |
| --- | --- |
| Drug-naïve | 18 |
| Aripiprazole | 16 |
| Olanzapine | 14 |
| Olanzapine + Aripiprazole | 1 |
| Olanzapine + Haloperidol injection | 4 |
| Olanzapine + Risperidone | 1 |
| Haloperidol injection | 2 |
| Quetiapine | 1 |
| Risperidone | 48 |
| Risperidone + Haloperidol injection | 15 |
| Risperidone + Quetiapine | 1 |
| Paliperidone | 6 |
| Ziprasidone | 1 |
| Chlorpromazine equivalents were 281.18±163.50 mg/day. | |

## Supplementary Table 2. Fifty-four differentially expressed genes (DEGs) associated with monocytes

| **EntrezID** | **logFC** | **adj.P.Val** | **B** | **Symbols** | **Name** | **Monocyte subtype** | **Ref** |
| --- | --- | --- | --- | --- | --- | --- | --- |
| 3689 | 0,2009 | 0,000592 | -0,15164 | ITGB2 | integrin subunit beta 2 | all monocytes | [23](https://science.sciencemag.org/content/356/6335/eaah4573) |
| 64581 | 0,27028 | 0,000999 | -0,67474 | CLEC7A | C-type lectin domain containing 7A | all monocytes | [23](https://science.sciencemag.org/content/356/6335/eaah4573) |
| 23166 | 0,2237 | 0,003023 | -1,7737 | STAB1 | stabilin 1 | all monocytes | [23](https://science.sciencemag.org/content/356/6335/eaah4573) |
| 7097 | 0,14749 | 0,025824 | -3,8399 | TLR2 | toll like receptor 2 | all monocytes | [23](https://science.sciencemag.org/content/356/6335/eaah4573) |
| 4671 | 0,4043 | 4,91E-06 | 4,7321 | NAIP | NLR family apoptosis inhibitory protein | classical | [23](https://science.sciencemag.org/content/356/6335/eaah4573) |
| 948 | -0,4177 | 3,48E-05 | 2,7153 | CD36 | CD36 molecule | classical | [7](https://science.sciencemag.org/content/356/6335/eaah4573) |
| 7421 | 0,27822 | 0,000183 | 1,0295 | VDR | vitamin D receptor | classical | [24](https://science.sciencemag.org/content/356/6335/eaah4573) |
| 4035 | -0,2998 | 0,002357 | -1,5272 | LRP1 | LDL receptor related protein 1 | classical | [22](https://science.sciencemag.org/content/356/6335/eaah4573) |
| 6036 | -0,41415 | 0,002966 | -1,7544 | RNASE2 | ribonuclease A family member 2 | classical | [23](https://science.sciencemag.org/content/356/6335/eaah4573) |
| 2162 | -0,31858 | 0,004952 | -2,2558 | F13A1 | coagulation factor XIII A chain | classical | [23](https://science.sciencemag.org/content/356/6335/eaah4573) |
| 9332 | -0,25506 | 0,01195 | -3,1068 | CD163 | CD163 molecule | classical | [23](https://science.sciencemag.org/content/356/6335/eaah4573) |
| 1378 | 0,23104 | 0,027237 | -3,8917 | CR1 | complement C3b/C4b receptor 1 (Knops blood group) | classical | [22](https://science.sciencemag.org/content/356/6335/eaah4573) |
| 729230 | -0,14532 | 0,041602 | -4,2928 | CCR2 | C-C motif chemokine receptor 2 | classical | [7](https://science.sciencemag.org/content/356/6335/eaah4573) |
| 6279 | -1,4033 | 3,70E-09 | 12,504 | S100A8 | S100 calcium binding protein A8 | intermediate | [24](https://science.sciencemag.org/content/356/6335/eaah4573) |
| 6280 | -0,85023 | 5,13E-08 | 9,5721 | S100A9 | S100 calcium binding protein A9 | intermediate | [24](https://science.sciencemag.org/content/356/6335/eaah4573) |
| 5552 | -0,58497 | 5,63E-08 | 9,4665 | SRGN | serglycin | intermediate | [23](https://science.sciencemag.org/content/356/6335/eaah4573) |
| 3687 | 0,46688 | 8,71E-08 | 8,9899 | ITGAX | integrin subunit alpha X | intermediate | [7](https://science.sciencemag.org/content/356/6335/eaah4573) |
| 3122 | -0,41035 | 1,26E-06 | 6,1415 | HLA-DRA | "major histocompatibility complex, class II, DR alpha" | intermediate | 22; 7 |
| 942 | -0,34474 | 3,30E-06 | 5,1376 | CD86 | CD86 molecule | intermediate | [7](https://science.sciencemag.org/content/356/6335/eaah4573) |
| 1234 | -0,3195 | 6,63E-06 | 4,419 | CCR5 | C-C motif chemokine receptor 5 (gene/pseudogene) | intermediate | [7](https://science.sciencemag.org/content/356/6335/eaah4573) |
| 3579 | 0,41402 | 5,49E-05 | 2,252 | CXCR2 | C-X-C motif chemokine receptor 2 | intermediate | [23](https://science.sciencemag.org/content/356/6335/eaah4573) |
| 3123 | -0,281 | 0,000278 | 0,60828 | HLA-DRB1 | "major histocompatibility complex, class II, DR beta 1" | intermediate | 22; 7 |
| 4084 | 0,34228 | 0,000467 | 0,085827 | MXD1 | MAX dimerization protein 1 | intermediate | [23](https://science.sciencemag.org/content/356/6335/eaah4573) |
| 2212 | 0,27557 | 0,001305 | -0,94105 | FCGR2A | Fc fragment of IgG receptor IIa | intermediate | [23](https://science.sciencemag.org/content/356/6335/eaah4573) |
| 5055 | -0,36468 | 0,00176 | -1,2374 | SERPINB2 | serpin family B member 2 | intermediate | [24](https://science.sciencemag.org/content/356/6335/eaah4573) |
| 8794 | 0,24893 | 0,004726 | -2,2103 | TNFRSF10C | TNF receptor superfamily member 10c | intermediate | [23](https://science.sciencemag.org/content/356/6335/eaah4573) |
| 26253 | -0,23971 | 0,005349 | -2,332 | CLEC4E | C-type lectin domain family 4 member E | intermediate | [23](https://science.sciencemag.org/content/356/6335/eaah4573) |
| 2022 | 0,13001 | 0,011538 | -3,0718 | ENG | endoglin | intermediate | [22](https://science.sciencemag.org/content/356/6335/eaah4573) |
| 8875 | 0,22814 | 0,026246 | -3,8564 | VNN2 | vanin 2 | intermediate | [23](https://science.sciencemag.org/content/356/6335/eaah4573) |
| 10135 | -0,21804 | 0,026301 | -3,8583 | NAMPT | nicotinamide phosphoribosyltransferase | intermediate | [23](https://science.sciencemag.org/content/356/6335/eaah4573) |
| 60675 | 0,22245 | 0,047389 | -4,4152 | PROK2 | prokineticin 2 | intermediate | [24](https://science.sciencemag.org/content/356/6335/eaah4573) |
| 1441 | 0,58175 | 3,28E-08 | 10,059 | CSF3R | colony stimulating factor 3 receptor | intermediate/classical | [23; 24](https://science.sciencemag.org/content/356/6335/eaah4573) |
| 6283 | -0,68942 | 0,000132 | 1,3582 | S100A12 | S100 calcium binding protein A12 | intermediate/classical | [23; 24](https://science.sciencemag.org/content/356/6335/eaah4573;) |
| 10288 | 0,4683 | 6,08E-09 | 11,947 | LILRB2 | leukocyte immunoglobulin like receptor B2 | nonclassical | [23](https://science.sciencemag.org/content/356/6335/eaah4573) |
| 10410 | 0,91214 | 2,44E-08 | 10,385 | IFITM3 | interferon induced transmembrane protein 3 | nonclassical | [23](https://science.sciencemag.org/content/356/6335/eaah4573) |
| 140885 | 0,46071 | 7,24E-08 | 9,1884 | SIRPA | signal regulatory protein alpha | nonclassical | [22](https://science.sciencemag.org/content/356/6335/eaah4573) |
| 1520 | -0,3716 | 2,32E-07 | 7,936 | CTSS | cathepsin S | nonclassical | [23](https://science.sciencemag.org/content/356/6335/eaah4573) |
| 10581 | 0,57846 | 4,92E-06 | 4,7288 | IFITM2 | interferon induced transmembrane protein 2 | nonclassical | [23](https://science.sciencemag.org/content/356/6335/eaah4573) |
| 80139 | 0,36842 | 2,20E-05 | 3,1855 | ZNF703 | zinc finger protein 703 | nonclassical | [24](https://science.sciencemag.org/content/356/6335/eaah4573) |
| 389 | -0,35686 | 2,57E-05 | 3,0248 | RHOC | ras homolog family member C | nonclassical | [23](https://science.sciencemag.org/content/356/6335/eaah4573) |
| 3055 | 0,34019 | 3,01E-05 | 2,8624 | HCK | "HCK proto-oncogene, Src family tyrosine kinase" | nonclassical | [23](https://science.sciencemag.org/content/356/6335/eaah4573) |
| 58475 | -0,37902 | 4,78E-05 | 2,3936 | MS4A7 | membrane spanning 4-domains A7 | nonclassical | [23](https://science.sciencemag.org/content/356/6335/eaah4573) |
| 5452 | 0,21121 | 5,22E-05 | 2,3022 | POU2F2 | POU class 2 homeobox 2 | nonclassical | [24](https://science.sciencemag.org/content/356/6335/eaah4573) |
| 9935 | 0,32123 | 0,000182 | 1,0352 | MAFB | MAF bZIP transcription factor B | nonclassical | [23](https://science.sciencemag.org/content/356/6335/eaah4573) |
| 2268 | 0,22931 | 0,000624 | -0,20576 | FGR | "FGR proto-oncogene, Src family tyrosine kinase" | nonclassical | [23](https://science.sciencemag.org/content/356/6335/eaah4573) |
| 7940 | -0,27575 | 0,000774 | -0,42147 | LST1 | leukocyte specific transcript 1 | nonclassical | [23](https://science.sciencemag.org/content/356/6335/eaah4573) |
| 283131 | 0,29852 | 0,001106 | -0,77496 | NEAT1 | nuclear paraspeckle assembly transcript 1 | nonclassical | [23](https://science.sciencemag.org/content/356/6335/eaah4573) |
| 5175 | -0,16705 | 0,002674 | -1,6531 | PECAM1 | platelet and endothelial cell adhesion molecule 1 | nonclassical | [22](https://science.sciencemag.org/content/356/6335/eaah4573) |
| 1436 | -0,19494 | 0,003335 | -1,8696 | CSF1R | colony stimulating factor 1 receptor | nonclassical | [23](https://science.sciencemag.org/content/356/6335/eaah4573) |
| 1028 | -0,32162 | 0,004222 | -2,0996 | CDKN1C | cyclin dependent kinase inhibitor 1C | nonclassical | [24](https://science.sciencemag.org/content/356/6335/eaah4573) |
| 7133 | 0,1577 | 0,004736 | -2,2126 | TNFRSF1B | TNF receptor superfamily member 1B | nonclassical | [23](https://science.sciencemag.org/content/356/6335/eaah4573) |
| 11027 | 0,18839 | 0,011014 | -3,0268 | LILRA2 | leukocyte immunoglobulin like receptor A2 | nonclassical | [23](https://science.sciencemag.org/content/356/6335/eaah4573) |
| 9728 | -0,20608 | 0,018111 | -3,5047 | SECISBP2L | SECIS binding protein 2 like | nonclassical | [7](https://science.sciencemag.org/content/356/6335/eaah4573) |
| 89790 | 0,18799 | 0,018235 | -3,5117 | SIGLEC10 | sialic acid binding Ig like lectin 10 | nonclassical | [23](https://science.sciencemag.org/content/356/6335/eaah4573) |

## Supplementary Table 3. DAVID GOBP and KEGG pathway annotations of 54 DEGs

| Annotation Cluster 1 | Enrichment Score: 5.26164045956125 |  |  |  |
| --- | --- | --- | --- | --- |
| Category | Term | Count | Genes | FDR |
| GOTERM_BP_ALL | GO:0050900~leukocyte migration | 12 | CSF1R, HCK, CSF3R, ITGB2, CXCR2, PECAM1, SIRPA, S100A12, CCR5, S100A9, S100A8, CCR2 | 4.87E-7 |
| GOTERM_BP_ALL | GO:0097529~myeloid leukocyte migration | 10 | CSF1R, CSF3R, ITGB2, CXCR2, PECAM1, SIRPA, S100A12, S100A9, S100A8, CCR2 | 6.50E-7 |
| GOTERM_BP_ALL | GO:0071674~mononuclear cell migration | 5 | CSF1R, PECAM1, SIRPA, S100A12, CCR2 | 0.002 |
| GOTERM_BP_ALL | GO:0035696~monocyte extravasation | 3 | PECAM1, SIRPA, CCR2 | 0.002 |
| GOTERM_BP_ALL | GO:0045123~cellular extravasation | 4 | ITGB2, PECAM1, SIRPA, CCR2 | 0.008 |
|  |  |  |  |  |
| Annotation Cluster 2 | Enrichment Score: 3.941132414211531 |  |  |  |
| Category | Term | Count | Genes | FDR |
| GOTERM_BP_ALL | GO:0050900~leukocyte migration | 12 | CSF1R, HCK, CSF3R, ITGB2, CXCR2, PECAM1, SIRPA, S100A12, CCR5, S100A9, S100A8, CCR2 | 4.87E-7 |
| GOTERM_BP_ALL | GO:0097529~myeloid leukocyte migration | 10 | CSF1R, CSF3R, ITGB2, CXCR2, PECAM1, SIRPA, S100A12, S100A9, S100A8, CCR2 | 6.50E-7 |
| GOTERM_BP_ALL | GO:0050727~regulation of inflammatory response | 12 | FGR, HCK, CR1, CLEC7A, SIRPA, S100A12, NEAT1, S100A9, HLA-DRB1, S100A8, CCR2, TLR2 | 1.50E-6 |
| GOTERM_BP_ALL | GO:0032103~positive regulation of response to external stimulus | 11 | CSF1R, LRP1, CLEC7A, VDR, CXCR2, S100A12, NEAT1, S100A9, S100A8, CCR2, TLR2 | 2.16E-6 |
| GOTERM_BP_ALL | GO:0016477~cell migration | 19 | CSF1R, CSF3R, LRP1, ITGB2, RHOC, FGR, HCK, CLEC7A, ZNF703, CXCR2, ITGAX, PECAM1, S100A12, SIRPA, CCR5, S100A9, S100A8, CCR2, ENG | 3.01E-6 |
| GOTERM_BP_ALL | GO:0006935~chemotaxis | 13 | CSF1R, CSF3R, LRP1, ITGB2, RNASE2, CXCR2, PROK2, S100A12, CCR5, S100A9, S100A8, CCR2, ENG | 4.69E-6 |
| GOTERM_BP_ALL | GO:0042330~taxis | 13 | CSF1R, CSF3R, LRP1, ITGB2, RNASE2, CXCR2, PROK2, S100A12, CCR5, S100A9, S100A8, CCR2, ENG | 4.85E-6 |
| GOTERM_BP_ALL | GO:0040011~locomotion | 21 | CSF1R, CSF3R, LRP1, ITGB2, RHOC, RNASE2, FGR, HCK, CLEC7A, ZNF703, CXCR2, ITGAX, PECAM1, PROK2, S100A12, SIRPA, CCR5, S100A9, S100A8, CCR2, ENG | 5.43E-6 |
| GOTERM_BP_ALL | GO:0097530~granulocyte migration | 8 | CSF1R, CSF3R, ITGB2, CXCR2, PECAM1, S100A12, S100A9, S100A8 | 7.50E-6 |
| GOTERM_BP_ALL | GO:0060326~cell chemotaxis | 10 | CSF1R, CSF3R, ITGB2, CXCR2, S100A12, CCR5, S100A9, S100A8, CCR2, ENG | 7.90E-6 |
| GOTERM_BP_ALL | GO:0030595~leukocyte chemotaxis | 9 | CSF1R, CSF3R, ITGB2, CXCR2, S100A12, CCR5, S100A9, S100A8, CCR2 | 1.06E-5 |
| GOTERM_BP_ALL | GO:0051674~localization of cell | 19 | CSF1R, CSF3R, LRP1, ITGB2, RHOC, FGR, HCK, CLEC7A, ZNF703, CXCR2, ITGAX, PECAM1, S100A12, SIRPA, CCR5, S100A9, S100A8, CCR2, ENG | 1.72E-5 |
| GOTERM_BP_ALL | GO:0048870~cell motility | 19 | CSF1R, CSF3R, LRP1, ITGB2, RHOC, FGR, HCK, CLEC7A, ZNF703, CXCR2, ITGAX, PECAM1, S100A12, SIRPA, CCR5, S100A9, S100A8, CCR2, ENG | 1.72E-5 |
| GOTERM_BP_ALL | GO:1990266~neutrophil migration | 7 | CSF3R, ITGB2, CXCR2, PECAM1, S100A12, S100A9, S100A8 | 3.37E-5 |
| GOTERM_BP_ALL | GO:0071621~granulocyte chemotaxis | 7 | CSF1R, CSF3R, ITGB2, CXCR2, S100A12, S100A9, S100A8 | 3.75E-5 |
| GOTERM_BP_ALL | GO:0032879~regulation of localization | 23 | CSF1R, LRP1, ITGB2, LILRB2, RHOC, LILRA2, TNFRSF1B, CTSS, FGR, HCK, CLEC7A, ZNF703, CXCR2, ITGAX, PECAM1, SIRPA, CD36, CCR5, S100A8, HLA-DRB1, CCR2, ENG, TLR2 | 8.51E-5 |
| GOTERM_BP_ALL | GO:0065009~regulation of molecular function | 24 | CDKN1C, CD86, CSF1R, CR1, SERPINB2, LRP1, VDR, ITGB2, RHOC, CTSS, FGR, HCK, CLEC7A, PROK2, S100A12, SIRPA, CD36, S100A9, S100A8, HLA-DRB1, NAIP, CCR2, ENG, TLR2 | 9.26E-5 |
| GOTERM_BP_ALL | GO:0051092~positive regulation of NF-kappaB transcription factor activity | 7 | CLEC7A, ITGB2, S100A12, CD36, S100A9, S100A8, TLR2 | 9.62E-5 |
| GOTERM_BP_ALL | GO:0050729~positive regulation of inflammatory response | 7 | CLEC7A, S100A12, NEAT1, S100A9, S100A8, CCR2, TLR2 | 1.02E-4 |
| GOTERM_BP_ALL | GO:0030593~neutrophil chemotaxis | 6 | CSF3R, ITGB2, CXCR2, S100A12, S100A9, S100A8 | 1.56E-4 |
| GOTERM_BP_ALL | GO:0006928~movement of cell or subcellular component | 19 | CSF1R, CSF3R, LRP1, ITGB2, RHOC, FGR, HCK, CLEC7A, ZNF703, CXCR2, ITGAX, PECAM1, S100A12, SIRPA, CCR5, S100A9, S100A8, CCR2, ENG | 2.30E-4 |
| GOTERM_BP_ALL | GO:0050832~defense response to fungus | 5 | CLEC7A, S100A12, CLEC4E, S100A9, S100A8 | 3.74E-4 |
| GOTERM_BP_ALL | GO:0009620~response to fungus | 5 | CLEC7A, S100A12, CLEC4E, S100A9, S100A8 | 6.12E-4 |
| GOTERM_BP_ALL | GO:0035556~intracellular signal transduction | 20 | CD86, CSF1R, LRP1, RHOC, TNFRSF1B, FGR, CLEC7A, CXCR2, PROK2, S100A12, SIRPA, CD36, CCR5, S100A9, S100A8, HLA-DRB1, NAIP, CCR2, ENG, TLR2 | 8.72E-4 |
| GOTERM_BP_ALL | GO:0051091~positive regulation of sequence-specific DNA binding transcription factor activity | 7 | CLEC7A, ITGB2, S100A12, CD36, S100A9, S100A8, TLR2 | 0.001 |
| GOTERM_BP_ALL | GO:0010942~positive regulation of cell death | 10 | LRP1, CLEC7A, VDR, ITGB2, CXCR2, TNFRSF10C, CD36, TNFRSF1B, S100A9, S100A8 | 0.001 |
| GOTERM_BP_ALL | GO:0043085~positive regulation of catalytic activity | 13 | CD86, CSF1R, CR1, VDR, ITGB2, RHOC, FGR, CLEC7A, PROK2, S100A12, S100A9, S100A8, HLA-DRB1 | 0.002 |
| GOTERM_BP_ALL | GO:0051090~regulation of sequence-specific DNA binding transcription factor activity | 8 | HCK, CLEC7A, ITGB2, S100A12, CD36, S100A9, S100A8, TLR2 | 0.002 |
| GOTERM_BP_ALL | GO:0016192~vesicle-mediated transport | 13 | CD163, LRP1, ITGB2, FGR, HCK, CLEC7A, CXCR2, STAB1, PECAM1, SIRPA, CD36, CCR2, TLR2 | 0.008 |
| GOTERM_BP_ALL | GO:0044765~single-organism transport | 18 | CR1, LRP1, VDR, ITGB2, TNFRSF1B, FGR, HCK, CLEC7A, CXCR2, PECAM1, SIRPA, CD36, CCR5, S100A8, HLA-DRB1, CCR2, ENG, TLR2 | 0.01 |
| GOTERM_BP_ALL | GO:0002523~leukocyte migration involved in inflammatory response | 3 | ITGB2, S100A9, S100A8 | 0.01 |
| GOTERM_BP_ALL | GO:0052547~regulation of peptidase activity | 7 | CR1, SERPINB2, LRP1, CLEC7A, S100A9, S100A8, NAIP | 0.01 |
| GOTERM_BP_ALL | GO:0030162~regulation of proteolysis | 8 | CR1, SERPINB2, LRP1, CLEC7A, TNFRSF1B, S100A9, S100A8, NAIP | 0.03 |
| GOTERM_BP_ALL | GO:0044710~single-organism metabolic process | 21 | CSF1R, LRP1, VDR, ITGB2, CTSS, FGR, HCK, VNN2, CLEC7A, NAMPT, CXCR2, PROK2, S100A12, SIRPA, CD36, CCR5, HLA-DRB1, NAIP, CCR2, ENG, TLR2 | 0.03 |
| GOTERM_BP_ALL | GO:0052548~regulation of endopeptidase activity | 6 | CR1, SERPINB2, CLEC7A, S100A9, S100A8, NAIP | 0.03 |
| GOTERM_BP_ALL | GO:0043065~positive regulation of apoptotic process | 7 | CLEC7A, VDR, CXCR2, TNFRSF10C, TNFRSF1B, S100A9, S100A8 | 0.04 |
| GOTERM_BP_ALL | GO:0043068~positive regulation of programmed cell death | 7 | CLEC7A, VDR, CXCR2, TNFRSF10C, TNFRSF1B, S100A9, S100A8 | 0.04 |
|  |  |  |  |  |
| Annotation Cluster 3 | Enrichment Score: 3.7281587800774085 |  |  |  |
| Category | Term | Count | Genes | FDR |
| GOTERM_BP_ALL | GO:0002274~myeloid leukocyte activation | 8 | FGR, NAMPT, ITGB2, CXCR2, S100A12, LILRA2, CCR2, TLR2 | 6.08E-5 |
| GOTERM_BP_ALL | GO:0036230~granulocyte activation | 4 | ITGB2, CXCR2, LILRA2, CCR2 | 0.003 |
| GOTERM_BP_ALL | GO:0042119~neutrophil activation | 3 | ITGB2, CXCR2, LILRA2 | 0.03 |
|  |  |  |  |  |
| Annotation Cluster 4 | Enrichment Score: 3.7117318007345625 |  |  |  |
| Category | Term | Count | Genes | FDR |
| GOTERM_BP_ALL | GO:0009605~response to external stimulus | 36 | IFITM3, CD86, CSF1R, IFITM2, CSF3R, LRP1, ITGB2, LILRA2, CLEC7A, NAMPT, CXCR2, STAB1, ITGAX, PROK2, S100A12, SIRPA, CD36, CCR5, CCR2, CR1, SERPINB2, VDR, LILRB2, POU2F2, RNASE2, TNFRSF1B, NEAT1, FGR, HCK, CLEC4E, S100A9, S100A8, HLA-DRB1, NAIP, ENG, TLR2 | 3.48E-15 |
| GOTERM_BP_ALL | GO:0006955~immune response | 31 | IFITM3, CD86, CSF1R, IFITM2, LRP1, LST1, ITGB2, LILRA2, CTSS, CLEC7A, CXCR2, S100A12, SIRPA, CD36, CCR5, CCR2, CR1, SIGLEC10, LILRB2, POU2F2, RNASE2, TNFRSF1B, FGR, HCK, HLA-DRA, CLEC4E, S100A9, S100A8, HLA-DRB1, NAIP, TLR2 | 5.27E-14 |
| GOTERM_BP_ALL | GO:0006954~inflammatory response | 22 | CSF1R, CD163, CR1, ITGB2, TNFRSF1B, NEAT1, FGR, HCK, CLEC7A, CXCR2, STAB1, PROK2, S100A12, SIRPA, CD36, CCR5, S100A9, S100A8, HLA-DRB1, NAIP, CCR2, TLR2 | 1.96E-13 |
| GOTERM_BP_ALL | GO:0045321~leukocyte activation | 20 | CD86, CR1, LST1, ITGB2, LILRB2, POU2F2, LILRA2, TNFRSF1B, FGR, MAFB, CLEC7A, NAMPT, CXCR2, S100A12, HLA-DRA, SIRPA, CLEC4E, HLA-DRB1, CCR2, TLR2 | 1.48E-9 |
| GOTERM_BP_ALL | GO:0001775~cell activation | 21 | CD86, CR1, LRP1, LST1, ITGB2, LILRB2, POU2F2, LILRA2, TNFRSF1B, FGR, MAFB, CLEC7A, NAMPT, CXCR2, S100A12, HLA-DRA, SIRPA, CLEC4E, HLA-DRB1, CCR2, TLR2 | 1.48E-9 |
| GOTERM_BP_ALL | GO:0002252~immune effector process | 19 | IFITM3, CD86, CR1, IFITM2, LRP1, ITGB2, LILRA2, RNASE2, TNFRSF1B, FGR, HCK, CLEC7A, ITGAX, HLA-DRA, CD36, CLEC4E, HLA-DRB1, CCR2, TLR2 | 2.70E-9 |
| GOTERM_BP_ALL | GO:0032101~regulation of response to external stimulus | 19 | CSF1R, CR1, SERPINB2, LRP1, VDR, LILRA2, NEAT1, FGR, HCK, CLEC7A, CXCR2, S100A12, SIRPA, CD36, S100A9, S100A8, HLA-DRB1, CCR2, TLR2 | 3.48E-9 |
| GOTERM_BP_ALL | GO:0002682~regulation of immune system process | 23 | CD86, CSF1R, CSF3R, CR1, LST1, ITGB2, LILRB2, LILRA2, TNFRSF1B, CTSS, FGR, HCK, FCGR2A, MAFB, CLEC7A, CXCR2, HLA-DRA, SIRPA, CD36, CLEC4E, HLA-DRB1, CCR2, TLR2 | 4.60E-9 |
| GOTERM_BP_ALL | GO:0045087~innate immune response | 19 | IFITM3, CSF1R, CR1, IFITM2, SIGLEC10, LILRA2, RNASE2, CTSS, FGR, HCK, CLEC7A, S100A12, SIRPA, CD36, CLEC4E, S100A9, S100A8, NAIP, TLR2 | 6.04E-9 |
| GOTERM_BP_ALL | GO:0051240~positive regulation of multicellular organismal process | 24 | CD86, CSF1R, CR1, LRP1, VDR, ITGB2, LILRB2, POU2F2, LILRA2, TNFRSF1B, FGR, CLEC7A, ZNF703, CXCR2, ITGAX, PROK2, HLA-DRA, CD36, CLEC4E, S100A9, HLA-DRB1, CCR2, ENG, TLR2 | 1.90E-8 |
| GOTERM_BP_ALL | GO:0007155~cell adhesion | 22 | CD86, CSF3R, CR1, LRP1, ITGB2, SIGLEC10, LILRB2, TNFRSF1B, HCK, CLEC7A, ZNF703, STAB1, ITGAX, PECAM1, HLA-DRA, SIRPA, CD36, S100A9, S100A8, HLA-DRB1, CCR2, ENG | 2.43E-8 |
| GOTERM_BP_ALL | GO:0022610~biological adhesion | 22 | CD86, CSF3R, CR1, LRP1, ITGB2, SIGLEC10, LILRB2, TNFRSF1B, HCK, CLEC7A, ZNF703, STAB1, ITGAX, PECAM1, HLA-DRA, SIRPA, CD36, S100A9, S100A8, HLA-DRB1, CCR2, ENG | 2.53E-8 |
| GOTERM_BP_ALL | GO:0009617~response to bacterium | 17 | CD86, LILRB2, LILRA2, TNFRSF1B, FGR, HCK, STAB1, S100A12, SIRPA, CD36, CLEC4E, CCR5, S100A9, S100A8, HLA-DRB1, NAIP, TLR2 | 2.81E-8 |
| GOTERM_BP_ALL | GO:0001816~cytokine production | 17 | CD86, SRGN, CSF1R, CR1, LRP1, LILRB2, POU2F2, LILRA2, TNFRSF1B, FGR, CLEC7A, SIRPA, CD36, CLEC4E, HLA-DRB1, CCR2, TLR2 | 3.46E-8 |
| GOTERM_BP_ALL | GO:0001817~regulation of cytokine production | 17 | CD86, SRGN, CSF1R, CR1, LRP1, LILRB2, POU2F2, LILRA2, TNFRSF1B, FGR, CLEC7A, SIRPA, CD36, CLEC4E, HLA-DRB1, CCR2, TLR2 | 4.56E-8 |
| GOTERM_BP_ALL | GO:0070887~cellular response to chemical stimulus | 29 | CDKN1C, IFITM3, CD86, CSF1R, IFITM2, CSF3R, LRP1, ITGB2, LILRA2, CTSS, CLEC7A, ZNF703, NAMPT, CXCR2, S100A12, SIRPA, CD36, CCR5, CCR2, VDR, LILRB2, TNFRSF1B, HCK, S100A9, S100A8, HLA-DRB1, NAIP, ENG, TLR2 | 5.90E-8 |
| GOTERM_BP_ALL | GO:0071310~cellular response to organic substance | 26 | CDKN1C, IFITM3, CD86, CSF1R, IFITM2, CSF3R, LRP1, ITGB2, LILRA2, CTSS, CLEC7A, ZNF703, NAMPT, CXCR2, SIRPA, CD36, CCR5, CCR2, VDR, LILRB2, TNFRSF1B, HCK, HLA-DRB1, NAIP, ENG, TLR2 | 1.06E-7 |
| GOTERM_BP_ALL | GO:0031347~regulation of defense response | 16 | CR1, LILRA2, NEAT1, CTSS, FGR, HCK, CLEC7A, S100A12, SIRPA, CD36, CLEC4E, S100A9, S100A8, HLA-DRB1, CCR2, TLR2 | 1.18E-7 |
| GOTERM_BP_ALL | GO:0050776~regulation of immune response | 17 | CD86, CR1, ITGB2, LILRB2, LILRA2, TNFRSF1B, CTSS, FGR, HCK, FCGR2A, CLEC7A, HLA-DRA, CD36, CLEC4E, HLA-DRB1, CCR2, TLR2 | 1.88E-7 |
| GOTERM_BP_ALL | GO:0002684~positive regulation of immune system process | 18 | CD86, CSF1R, CR1, ITGB2, LILRB2, LILRA2, CTSS, FGR, HCK, CLEC7A, CXCR2, HLA-DRA, SIRPA, CD36, CLEC4E, HLA-DRB1, CCR2, TLR2 | 2.16E-7 |
| GOTERM_BP_ALL | GO:0002263~cell activation involved in immune response | 11 | FGR, CD86, CR1, LRP1, CLEC7A, ITGB2, HLA-DRA, LILRA2, CLEC4E, HLA-DRB1, CCR2 | 3.31E-7 |
| GOTERM_BP_ALL | GO:0010033~response to organic substance | 28 | CDKN1C, IFITM3, CD86, CSF1R, IFITM2, CSF3R, LRP1, ITGB2, LILRA2, CTSS, CLEC7A, ZNF703, NAMPT, CXCR2, SIRPA, CD36, CCR5, CCR2, VDR, LILRB2, TNFRSF1B, HCK, S100A9, S100A8, HLA-DRB1, NAIP, ENG, TLR2 | 6.04E-7 |
| GOTERM_BP_ALL | GO:0016337~single organismal cell-cell adhesion | 14 | CD86, CR1, ITGB2, LILRB2, TNFRSF1B, ZNF703, ITGAX, PECAM1, HLA-DRA, SIRPA, S100A9, S100A8, HLA-DRB1, CCR2 | 6.38E-7 |
| GOTERM_BP_ALL | GO:0080134~regulation of response to stress | 20 | CR1, SERPINB2, LRP1, LILRA2, NEAT1, CTSS, FGR, HCK, CLEC7A, NAMPT, S100A12, SIRPA, CD36, CLEC4E, S100A9, S100A8, HLA-DRB1, NAIP, CCR2, TLR2 | 8.34E-7 |
| GOTERM_BP_ALL | GO:0007159~leukocyte cell-cell adhesion | 12 | CD86, CR1, ITGB2, PECAM1, HLA-DRA, SIRPA, LILRB2, TNFRSF1B, S100A9, HLA-DRB1, S100A8, CCR2 | 1.01E-6 |
| GOTERM_BP_ALL | GO:0098542~defense response to other organism | 14 | IFITM3, IFITM2, RNASE2, FGR, CLEC7A, STAB1, ITGAX, S100A12, CD36, CLEC4E, S100A9, S100A8, NAIP, TLR2 | 1.33E-6 |
| GOTERM_BP_ALL | GO:0050727~regulation of inflammatory response | 12 | FGR, HCK, CR1, CLEC7A, SIRPA, S100A12, NEAT1, S100A9, HLA-DRB1, S100A8, CCR2, TLR2 | 1.50E-6 |
| GOTERM_BP_ALL | GO:0046649~lymphocyte activation | 15 | CD86, CR1, LST1, ITGB2, LILRB2, POU2F2, TNFRSF1B, FGR, MAFB, CLEC7A, HLA-DRA, SIRPA, CLEC4E, HLA-DRB1, CCR2 | 1.68E-6 |
| GOTERM_BP_ALL | GO:0071216~cellular response to biotic stimulus | 10 | CD86, HCK, CLEC7A, SIRPA, LILRB2, CD36, CCR5, LILRA2, TNFRSF1B, TLR2 | 2.05E-6 |
| GOTERM_BP_ALL | GO:0032103~positive regulation of response to external stimulus | 11 | CSF1R, LRP1, CLEC7A, VDR, CXCR2, S100A12, NEAT1, S100A9, S100A8, CCR2, TLR2 | 2.16E-6 |
| GOTERM_BP_ALL | GO:0098602~single organism cell adhesion | 14 | CD86, CR1, ITGB2, LILRB2, TNFRSF1B, ZNF703, ITGAX, PECAM1, HLA-DRA, SIRPA, S100A9, S100A8, HLA-DRB1, CCR2 | 2.20E-6 |
| GOTERM_BP_ALL | GO:0032496~response to lipopolysaccharide | 11 | CD86, HCK, SIRPA, LILRB2, CD36, CCR5, LILRA2, TNFRSF1B, S100A9, S100A8, TLR2 | 2.48E-6 |
| GOTERM_BP_ALL | GO:0002366~leukocyte activation involved in immune response | 10 | FGR, CD86, CR1, CLEC7A, ITGB2, HLA-DRA, LILRA2, CLEC4E, HLA-DRB1, CCR2 | 3.24E-6 |
| GOTERM_BP_ALL | GO:0019221~cytokine-mediated signaling pathway | 12 | IFITM3, CSF1R, HCK, CSF3R, IFITM2, CXCR2, LILRB2, CCR5, LILRA2, TNFRSF1B, NAIP, CCR2 | 3.37E-6 |
| GOTERM_BP_ALL | GO:0002237~response to molecule of bacterial origin | 11 | CD86, HCK, SIRPA, LILRB2, CD36, CCR5, LILRA2, TNFRSF1B, S100A9, S100A8, TLR2 | 3.78E-6 |
| GOTERM_BP_ALL | GO:0050778~positive regulation of immune response | 14 | CD86, CR1, ITGB2, LILRA2, CTSS, FGR, HCK, CLEC7A, HLA-DRA, CD36, CLEC4E, HLA-DRB1, CCR2, TLR2 | 3.86E-6 |
| GOTERM_BP_ALL | GO:0001819~positive regulation of cytokine production | 12 | FGR, CD86, CSF1R, LRP1, CLEC7A, LILRB2, CD36, LILRA2, POU2F2, CLEC4E, CCR2, TLR2 | 4.71E-6 |
| GOTERM_BP_ALL | GO:0071222~cellular response to lipopolysaccharide | 9 | CD86, HCK, SIRPA, LILRB2, CD36, CCR5, LILRA2, TNFRSF1B, TLR2 | 6.27E-6 |
| GOTERM_BP_ALL | GO:0098609~cell-cell adhesion | 15 | CD86, CR1, ITGB2, LILRB2, TNFRSF1B, CLEC7A, ZNF703, ITGAX, PECAM1, HLA-DRA, SIRPA, S100A9, S100A8, HLA-DRB1, CCR2 | 7.90E-6 |
| GOTERM_BP_ALL | GO:0031349~positive regulation of defense response | 11 | HCK, CLEC7A, S100A12, LILRA2, CLEC4E, NEAT1, S100A9, CTSS, S100A8, CCR2, TLR2 | 8.40E-6 |
| GOTERM_BP_ALL | GO:0071219~cellular response to molecule of bacterial origin | 9 | CD86, HCK, SIRPA, LILRB2, CD36, CCR5, LILRA2, TNFRSF1B, TLR2 | 8.91E-6 |
| GOTERM_BP_ALL | GO:0051094~positive regulation of developmental process | 18 | CD86, CR1, LRP1, VDR, ITGB2, LILRB2, TNFRSF1B, CLEC7A, ZNF703, CXCR2, ITGAX, HLA-DRA, CD36, S100A9, HLA-DRB1, CCR2, ENG, TLR2 | 1.02E-5 |
| GOTERM_BP_ALL | GO:0050865~regulation of cell activation | 13 | CD86, CR1, LST1, ITGB2, LILRB2, LILRA2, TNFRSF1B, FGR, CLEC7A, HLA-DRA, SIRPA, HLA-DRB1, CCR2 | 1.12E-5 |
| GOTERM_BP_ALL | GO:0071345~cellular response to cytokine stimulus | 14 | IFITM3, CSF1R, CSF3R, IFITM2, LILRB2, LILRA2, TNFRSF1B, HCK, CXCR2, SIRPA, CCR5, NAIP, CCR2, TLR2 | 1.17E-5 |
| GOTERM_BP_ALL | GO:0042221~response to chemical | 31 | CDKN1C, IFITM3, CD86, CSF1R, IFITM2, CSF3R, LRP1, ITGB2, LILRA2, CTSS, CLEC7A, ZNF703, NAMPT, CXCR2, PROK2, S100A12, SIRPA, CD36, CCR5, CCR2, VDR, LILRB2, RNASE2, TNFRSF1B, HCK, S100A9, S100A8, HLA-DRB1, NAIP, ENG, TLR2 | 1.19E-5 |
| GOTERM_BP_ALL | GO:0050764~regulation of phagocytosis | 7 | FGR, HCK, LRP1, CLEC7A, SIRPA, CD36, TLR2 | 1.24E-5 |
| GOTERM_BP_ALL | GO:0002699~positive regulation of immune effector process | 9 | FGR, CD86, CR1, CLEC7A, ITGB2, HLA-DRA, CD36, HLA-DRB1, CCR2 | 1.31E-5 |
| GOTERM_BP_ALL | GO:0050867~positive regulation of cell activation | 11 | FGR, CD86, CR1, CLEC7A, ITGB2, HLA-DRA, SIRPA, LILRB2, LILRA2, HLA-DRB1, CCR2 | 1.37E-5 |
| GOTERM_BP_ALL | GO:0045597~positive regulation of cell differentiation | 15 | CD86, CR1, LRP1, VDR, LILRB2, TNFRSF1B, CLEC7A, ZNF703, HLA-DRA, CD36, S100A9, HLA-DRB1, CCR2, ENG, TLR2 | 1.91E-5 |
| GOTERM_BP_ALL | GO:0042110~T cell activation | 11 | CD86, CR1, MAFB, CLEC7A, HLA-DRA, SIRPA, LILRB2, CLEC4E, TNFRSF1B, HLA-DRB1, CCR2 | 3.13E-5 |
| GOTERM_BP_ALL | GO:0002694~regulation of leukocyte activation | 12 | FGR, CD86, CR1, CLEC7A, LST1, ITGB2, HLA-DRA, SIRPA, LILRB2, TNFRSF1B, HLA-DRB1, CCR2 | 3.37E-5 |
| GOTERM_BP_ALL | GO:1902533~positive regulation of intracellular signal transduction | 15 | CD86, CSF1R, LRP1, RHOC, FGR, CLEC7A, PROK2, S100A12, CD36, S100A9, S100A8, HLA-DRB1, NAIP, ENG, TLR2 | 3.40E-5 |
| GOTERM_BP_ALL | GO:0034097~response to cytokine | 14 | IFITM3, CSF1R, CSF3R, IFITM2, LILRB2, LILRA2, TNFRSF1B, HCK, CXCR2, SIRPA, CCR5, NAIP, CCR2, TLR2 | 3.75E-5 |
| GOTERM_BP_ALL | GO:0030155~regulation of cell adhesion | 13 | CD86, CR1, LRP1, ITGB2, LILRB2, TNFRSF1B, ZNF703, HLA-DRA, SIRPA, CD36, HLA-DRB1, CCR2, ENG | 3.75E-5 |
| GOTERM_BP_ALL | GO:0044419~interspecies interaction between organisms | 13 | IFITM3, CD86, CSF1R, CR1, IFITM2, POU2F2, RNASE2, FGR, ITGAX, CLEC4E, CCR5, HLA-DRB1, TLR2 | 3.75E-5 |
| GOTERM_BP_ALL | GO:0002683~negative regulation of immune system process | 11 | FGR, CD86, HCK, CR1, MAFB, LST1, CXCR2, LILRB2, LILRA2, HLA-DRB1, CCR2 | 4.36E-5 |
| GOTERM_BP_ALL | GO:0002697~regulation of immune effector process | 10 | FGR, CD86, CR1, CLEC7A, ITGB2, HLA-DRA, CD36, TNFRSF1B, HLA-DRB1, CCR2 | 5.07E-5 |
| GOTERM_BP_ALL | GO:0033993~response to lipid | 14 | CD86, VDR, LILRB2, LILRA2, TNFRSF1B, HCK, ZNF703, SIRPA, CD36, CCR5, S100A9, S100A8, ENG, TLR2 | 5.12E-5 |
| GOTERM_BP_ALL | GO:0051249~regulation of lymphocyte activation | 11 | FGR, CD86, CR1, CLEC7A, LST1, HLA-DRA, SIRPA, LILRB2, TNFRSF1B, HLA-DRB1, CCR2 | 5.48E-5 |
| GOTERM_BP_ALL | GO:0002274~myeloid leukocyte activation | 8 | FGR, NAMPT, ITGB2, CXCR2, S100A12, LILRA2, CCR2, TLR2 | 6.08E-5 |
| GOTERM_BP_ALL | GO:0006909~phagocytosis | 9 | FGR, HCK, LRP1, CLEC7A, ITGB2, PECAM1, SIRPA, CD36, TLR2 | 6.16E-5 |
| GOTERM_BP_ALL | GO:1901701~cellular response to oxygen-containing compound | 15 | CD86, LRP1, VDR, LILRB2, LILRA2, TNFRSF1B, HCK, CLEC7A, ZNF703, NAMPT, SIRPA, CD36, CCR5, HLA-DRB1, TLR2 | 6.16E-5 |
| GOTERM_BP_ALL | GO:0002521~leukocyte differentiation | 11 | CD86, CSF1R, CR1, MAFB, HLA-DRA, LILRB2, POU2F2, CLEC4E, HLA-DRB1, CCR2, TLR2 | 6.32E-5 |
| GOTERM_BP_ALL | GO:0002696~positive regulation of leukocyte activation | 10 | FGR, CD86, CR1, CLEC7A, ITGB2, HLA-DRA, SIRPA, LILRB2, HLA-DRB1, CCR2 | 6.32E-5 |
| GOTERM_BP_ALL | GO:0002703~regulation of leukocyte mediated immunity | 8 | FGR, CR1, CLEC7A, ITGB2, HLA-DRA, TNFRSF1B, HLA-DRB1, CCR2 | 6.95E-5 |
| GOTERM_BP_ALL | GO:0030097~hemopoiesis | 13 | CDKN1C, CD86, CSF1R, CSF3R, CR1, LILRB2, POU2F2, MAFB, HLA-DRA, CLEC4E, HLA-DRB1, CCR2, TLR2 | 7.62E-5 |
| GOTERM_BP_ALL | GO:0051092~positive regulation of NF-kappaB transcription factor activity | 7 | CLEC7A, ITGB2, S100A12, CD36, S100A9, S100A8, TLR2 | 9.62E-5 |
| GOTERM_BP_ALL | GO:0016032~viral process | 12 | IFITM3, FGR, CD86, CSF1R, CR1, IFITM2, ITGAX, CCR5, RNASE2, POU2F2, HLA-DRB1, TLR2 | 9.95E-5 |
| GOTERM_BP_ALL | GO:0050729~positive regulation of inflammatory response | 7 | CLEC7A, S100A12, NEAT1, S100A9, S100A8, CCR2, TLR2 | 1.021E-4 |
| GOTERM_BP_ALL | GO:1903039~positive regulation of leukocyte cell-cell adhesion | 8 | CD86, CR1, ITGB2, HLA-DRA, SIRPA, LILRB2, HLA-DRB1, CCR2 | 1.02E-4 |
| GOTERM_BP_ALL | GO:0048534~hematopoietic or lymphoid organ development | 13 | CDKN1C, CD86, CSF1R, CSF3R, CR1, LILRB2, POU2F2, MAFB, HLA-DRA, CLEC4E, HLA-DRB1, CCR2, TLR2 | 1.10E-4 |
| GOTERM_BP_ALL | GO:0032635~interleukin-6 production | 7 | CLEC7A, SIRPA, LILRB2, CD36, LILRA2, POU2F2, TLR2 | 1.16E-4 |
| GOTERM_BP_ALL | GO:0045088~regulation of innate immune response | 9 | FGR, HCK, CR1, CLEC7A, CD36, LILRA2, CLEC4E, CTSS, TLR2 | 1.17E-4 |
| GOTERM_BP_ALL | GO:0044764~multi-organism cellular process | 12 | IFITM3, FGR, CD86, CSF1R, CR1, IFITM2, ITGAX, CCR5, RNASE2, POU2F2, HLA-DRB1, TLR2 | 1.18E-4 |
| GOTERM_BP_ALL | GO:0006897~endocytosis | 12 | FGR, HCK, CD163, LRP1, CLEC7A, ITGB2, CXCR2, STAB1, PECAM1, SIRPA, CD36, TLR2 | 1.28E-4 |
| GOTERM_BP_ALL | GO:0022407~regulation of cell-cell adhesion | 10 | CD86, CR1, ITGB2, ZNF703, HLA-DRA, SIRPA, LILRB2, TNFRSF1B, HLA-DRB1, CCR2 | 1.29E-4 |
| GOTERM_BP_ALL | GO:0071396~cellular response to lipid | 11 | CD86, HCK, VDR, ZNF703, SIRPA, LILRB2, CD36, CCR5, LILRA2, TNFRSF1B, TLR2 | 1.39E-4 |
| GOTERM_BP_ALL | GO:0045595~regulation of cell differentiation | 18 | CDKN1C, CD86, CSF3R, CR1, LRP1, VDR, LILRB2, TNFRSF1B, MAFB, CLEC7A, ZNF703, HLA-DRA, CD36, S100A9, HLA-DRB1, CCR2, ENG, TLR2 | 1.39E-4 |
| GOTERM_BP_ALL | GO:0032755~positive regulation of interleukin-6 production | 6 | CLEC7A, LILRB2, CD36, LILRA2, POU2F2, TLR2 | 1.40E-4 |
| GOTERM_BP_ALL | GO:0002764~immune response-regulating signaling pathway | 10 | FGR, HCK, CR1, CLEC7A, LILRB2, LILRA2, CLEC4E, CTSS, HLA-DRB1, TLR2 | 1.40E-4 |
| GOTERM_BP_ALL | GO:0032675~regulation of interleukin-6 production | 7 | CLEC7A, SIRPA, LILRB2, CD36, LILRA2, POU2F2, TLR2 | 1.40E-4 |
| GOTERM_BP_ALL | GO:0044403~symbiosis, encompassing mutualism through parasitism | 12 | IFITM3, FGR, CD86, CSF1R, CR1, IFITM2, ITGAX, CCR5, RNASE2, POU2F2, HLA-DRB1, TLR2 | 1.51E-4 |
| GOTERM_BP_ALL | GO:0042742~defense response to bacterium | 9 | FGR, STAB1, S100A12, CD36, CLEC4E, S100A9, S100A8, NAIP, TLR2 | 1.69E-4 |
| GOTERM_BP_ALL | GO:0002520~immune system development | 13 | CDKN1C, CD86, CSF1R, CSF3R, CR1, LILRB2, POU2F2, MAFB, HLA-DRA, CLEC4E, HLA-DRB1, CCR2, TLR2 | 1.75E-4 |
| GOTERM_BP_ALL | GO:0030098~lymphocyte differentiation | 9 | CD86, CR1, MAFB, HLA-DRA, LILRB2, POU2F2, CLEC4E, HLA-DRB1, CCR2 | 1.75E-4 |
| GOTERM_BP_ALL | GO:1903037~regulation of leukocyte cell-cell adhesion | 9 | CD86, CR1, ITGB2, HLA-DRA, SIRPA, LILRB2, TNFRSF1B, HLA-DRB1, CCR2 | 1.76E-4 |
| GOTERM_BP_ALL | GO:1903557~positive regulation of tumor necrosis factor superfamily cytokine production | 6 | CD86, CLEC7A, CD36, LILRA2, CCR2, TLR2 | 1.95E-4 |
| GOTERM_BP_ALL | GO:1901700~response to oxygen-containing compound | 17 | CD86, LRP1, VDR, LILRB2, LILRA2, TNFRSF1B, HCK, CLEC7A, ZNF703, NAMPT, SIRPA, CD36, CCR5, S100A9, S100A8, HLA-DRB1, TLR2 | 1.98E-4 |
| GOTERM_BP_ALL | GO:0071706~tumor necrosis factor superfamily cytokine production | 7 | CD86, CLEC7A, SIRPA, CD36, LILRA2, CCR2, TLR2 | 1.98E-4 |
| GOTERM_BP_ALL | GO:1903555~regulation of tumor necrosis factor superfamily cytokine production | 7 | CD86, CLEC7A, SIRPA, CD36, LILRA2, CCR2, TLR2 | 1.98E-4 |
| GOTERM_BP_ALL | GO:0022409~positive regulation of cell-cell adhesion | 8 | CD86, CR1, ITGB2, HLA-DRA, SIRPA, LILRB2, HLA-DRB1, CCR2 | 2.32E-4 |
| GOTERM_BP_ALL | GO:0002695~negative regulation of leukocyte activation | 7 | FGR, CD86, CR1, LST1, LILRB2, HLA-DRB1, CCR2 | 2.44E-4 |
| GOTERM_BP_ALL | GO:0098543~detection of other organism | 4 | CLEC7A, HLA-DRB1, NAIP, TLR2 | 2.96E-4 |
| GOTERM_BP_ALL | GO:0002440~production of molecular mediator of immune response | 8 | CD86, CR1, CLEC7A, HLA-DRA, CD36, TNFRSF1B, HLA-DRB1, CCR2 | 3.12E-4 |
| GOTERM_BP_ALL | GO:0045591~positive regulation of regulatory T cell differentiation | 4 | CR1, HLA-DRA, LILRB2, HLA-DRB1 | 3.30E-4 |
| GOTERM_BP_ALL | GO:0050866~negative regulation of cell activation | 7 | FGR, CD86, CR1, LST1, LILRB2, HLA-DRB1, CCR2 | 4.06E-4 |
| GOTERM_BP_ALL | GO:0070661~leukocyte proliferation | 8 | CD86, CSF1R, CR1, LST1, LILRB2, TNFRSF1B, HLA-DRB1, CCR2 | 4.46E-4 |
| GOTERM_BP_ALL | GO:0045785~positive regulation of cell adhesion | 9 | CD86, CR1, ITGB2, HLA-DRA, SIRPA, LILRB2, CD36, HLA-DRB1, CCR2 | 4.52E-4 |
| GOTERM_BP_ALL | GO:0050870~positive regulation of T cell activation | 7 | CD86, CR1, HLA-DRA, SIRPA, LILRB2, HLA-DRB1, CCR2 | 4.72E-4 |
| GOTERM_BP_ALL | GO:0046718~viral entry into host cell | 6 | IFITM3, CD86, CR1, IFITM2, CCR5, HLA-DRB1 | 4.91E-4 |
| GOTERM_BP_ALL | GO:0002757~immune response-activating signal transduction | 9 | FGR, HCK, CR1, CLEC7A, LILRA2, CLEC4E, CTSS, HLA-DRB1, TLR2 | 5.21E-4 |
| GOTERM_BP_ALL | GO:0098581~detection of external biotic stimulus | 4 | CLEC7A, HLA-DRB1, NAIP, TLR2 | 5.24E-4 |
| GOTERM_BP_ALL | GO:0051806~entry into cell of other organism involved in symbiotic interaction | 6 | IFITM3, CD86, CR1, IFITM2, CCR5, HLA-DRB1 | 5.26E-4 |
| GOTERM_BP_ALL | GO:0030260~entry into host cell | 6 | IFITM3, CD86, CR1, IFITM2, CCR5, HLA-DRB1 | 5.26E-4 |
| GOTERM_BP_ALL | GO:0044409~entry into host | 6 | IFITM3, CD86, CR1, IFITM2, CCR5, HLA-DRB1 | 5.26E-4 |
| GOTERM_BP_ALL | GO:0051828~entry into other organism involved in symbiotic interaction | 6 | IFITM3, CD86, CR1, IFITM2, CCR5, HLA-DRB1 | 5.41E-4 |
| GOTERM_BP_ALL | GO:0002768~immune response-regulating cell surface receptor signaling pathway | 8 | FGR, HCK, CR1, CLEC7A, LILRB2, LILRA2, CLEC4E, HLA-DRB1 | 5.62E-4 |
| GOTERM_BP_ALL | GO:0050863~regulation of T cell activation | 8 | CD86, CR1, HLA-DRA, SIRPA, LILRB2, TNFRSF1B, HLA-DRB1, CCR2 | 5.67E-4 |
| GOTERM_BP_ALL | GO:0050670~regulation of lymphocyte proliferation | 7 | CD86, CR1, LST1, LILRB2, TNFRSF1B, HLA-DRB1, CCR2 | 5.67E-4 |
| GOTERM_BP_ALL | GO:0051050~positive regulation of transport | 12 | FGR, LRP1, CLEC7A, ITGB2, SIRPA, CD36, LILRA2, CTSS, HLA-DRB1, S100A8, CCR2, TLR2 | 5.88E-4 |
| GOTERM_BP_ALL | GO:0032944~regulation of mononuclear cell proliferation | 7 | CD86, CR1, LST1, LILRB2, TNFRSF1B, HLA-DRB1, CCR2 | 5.98E-4 |
| GOTERM_BP_ALL | GO:0070663~regulation of leukocyte proliferation | 7 | CD86, CR1, LST1, LILRB2, TNFRSF1B, HLA-DRB1, CCR2 | 7.96E-4 |
| GOTERM_BP_ALL | GO:0045580~regulation of T cell differentiation | 6 | CD86, CR1, HLA-DRA, LILRB2, HLA-DRB1, CCR2 | 8.24E-4 |
| GOTERM_BP_ALL | GO:0009595~detection of biotic stimulus | 4 | CLEC7A, HLA-DRB1, NAIP, TLR2 | 8.24E-4 |
| GOTERM_BP_ALL | GO:0035556~intracellular signal transduction | 20 | CD86, CSF1R, LRP1, RHOC, TNFRSF1B, FGR, CLEC7A, CXCR2, PROK2, S100A12, SIRPA, CD36, CCR5, S100A9, S100A8, HLA-DRB1, NAIP, CCR2, ENG, TLR2 | 8.72E-4 |
| GOTERM_BP_ALL | GO:0030217~T cell differentiation | 7 | CD86, CR1, MAFB, HLA-DRA, LILRB2, CLEC4E, HLA-DRB1 | 8.77E-4 |
| GOTERM_BP_ALL | GO:0046209~nitric oxide metabolic process | 5 | CLEC7A, ITGB2, SIRPA, CD36, TLR2 | 8.93E-4 |
| GOTERM_BP_ALL | GO:0045066~regulatory T cell differentiation | 4 | CR1, HLA-DRA, LILRB2, HLA-DRB1 | 9.59E-4 |
| GOTERM_BP_ALL | GO:2001057~reactive nitrogen species metabolic process | 5 | CLEC7A, ITGB2, SIRPA, CD36, TLR2 | 9.59E-4 |
| GOTERM_BP_ALL | GO:0051250~negative regulation of lymphocyte activation | 6 | FGR, CD86, CR1, LST1, LILRB2, HLA-DRB1 | 9.84E-4 |
| GOTERM_BP_ALL | GO:0051251~positive regulation of lymphocyte activation | 8 | CD86, CR1, CLEC7A, HLA-DRA, SIRPA, LILRB2, HLA-DRB1, CCR2 | 9.84E-4 |
| GOTERM_BP_ALL | GO:0051091~positive regulation of sequence-specific DNA binding transcription factor activity | 7 | CLEC7A, ITGB2, S100A12, CD36, S100A9, S100A8, TLR2 | 0.001 |
| GOTERM_BP_ALL | GO:0002700~regulation of production of molecular mediator of immune response | 6 | CD86, CR1, CLEC7A, CD36, TNFRSF1B, CCR2 | 0.001 |
| GOTERM_BP_ALL | GO:0050672~negative regulation of lymphocyte proliferation | 5 | CD86, CR1, LST1, LILRB2, HLA-DRB1 | 0.001 |
| GOTERM_BP_ALL | GO:0032945~negative regulation of mononuclear cell proliferation | 5 | CD86, CR1, LST1, LILRB2, HLA-DRB1 | 0.001 |
| GOTERM_BP_ALL | GO:0045589~regulation of regulatory T cell differentiation | 4 | CR1, HLA-DRA, LILRB2, HLA-DRB1 | 0.001 |
| GOTERM_BP_ALL | GO:0007249~I-kappaB kinase/NF-kappaB signaling | 7 | CLEC7A, SIRPA, S100A12, CD36, RHOC, HLA-DRB1, TLR2 | 0.001 |
| GOTERM_BP_ALL | GO:0002822~regulation of adaptive immune response based on somatic recombination of immune receptors built from immunoglobulin superfamily domains | 6 | CR1, CLEC7A, HLA-DRA, TNFRSF1B, HLA-DRB1, CCR2 | 0.001 |
| GOTERM_BP_ALL | GO:0032640~tumor necrosis factor production | 6 | CLEC7A, SIRPA, CD36, LILRA2, CCR2, TLR2 | 0.001 |
| GOTERM_BP_ALL | GO:0002253~activation of immune response | 9 | FGR, HCK, CR1, CLEC7A, LILRA2, CLEC4E, CTSS, HLA-DRB1, TLR2 | 0.001 |
| GOTERM_BP_ALL | GO:1903706~regulation of hemopoiesis | 8 | CD86, CSF3R, CR1, MAFB, HLA-DRA, LILRB2, HLA-DRB1, CCR2 | 0.001 |
| GOTERM_BP_ALL | GO:0070664~negative regulation of leukocyte proliferation | 5 | CD86, CR1, LST1, LILRB2, HLA-DRB1 | 0.001 |
| GOTERM_BP_ALL | GO:0010942~positive regulation of cell death | 10 | LRP1, CLEC7A, VDR, ITGB2, CXCR2, TNFRSF10C, CD36, TNFRSF1B, S100A9, S100A8 | 0.001 |
| GOTERM_BP_ALL | GO:0042129~regulation of T cell proliferation | 6 | CD86, CR1, LILRB2, TNFRSF1B, HLA-DRB1, CCR2 | 0.001 |
| GOTERM_BP_ALL | GO:0060627~regulation of vesicle-mediated transport | 9 | FGR, HCK, LRP1, CLEC7A, ITGB2, SIRPA, CD36, CCR2, TLR2 | 0.001 |
| GOTERM_BP_ALL | GO:0045582~positive regulation of T cell differentiation | 5 | CD86, CR1, HLA-DRA, LILRB2, HLA-DRB1 | 0.001 |
| GOTERM_BP_ALL | GO:1902531~regulation of intracellular signal transduction | 16 | CD86, CSF1R, LRP1, RHOC, FGR, CLEC7A, PROK2, S100A12, SIRPA, CD36, S100A9, S100A8, HLA-DRB1, NAIP, ENG, TLR2 | 0.001 |
| GOTERM_BP_ALL | GO:0032680~regulation of tumor necrosis factor production | 6 | CLEC7A, SIRPA, CD36, LILRA2, CCR2, TLR2 | 0.001 |
| GOTERM_BP_ALL | GO:0030100~regulation of endocytosis | 7 | FGR, HCK, LRP1, CLEC7A, SIRPA, CD36, TLR2 | 0.001 |
| GOTERM_BP_ALL | GO:1902105~regulation of leukocyte differentiation | 7 | CD86, CR1, MAFB, HLA-DRA, LILRB2, HLA-DRB1, CCR2 | 0.002 |
| GOTERM_BP_ALL | GO:0045619~regulation of lymphocyte differentiation | 6 | CD86, CR1, HLA-DRA, LILRB2, HLA-DRB1, CCR2 | 0.002 |
| GOTERM_BP_ALL | GO:0032602~chemokine production | 5 | CSF1R, LRP1, CLEC7A, SIRPA, TLR2 | 0.002 |
| GOTERM_BP_ALL | GO:0002819~regulation of adaptive immune response | 6 | CR1, CLEC7A, HLA-DRA, TNFRSF1B, HLA-DRB1, CCR2 | 0.002 |
| GOTERM_BP_ALL | GO:0002758~innate immune response-activating signal transduction | 6 | HCK, CLEC7A, LILRA2, CLEC4E, CTSS, TLR2 | 0.002 |
| GOTERM_BP_ALL | GO:0046651~lymphocyte proliferation | 7 | CD86, CR1, LST1, LILRB2, TNFRSF1B, HLA-DRB1, CCR2 | 0.002 |
| GOTERM_BP_ALL | GO:0032642~regulation of chemokine production | 5 | CSF1R, LRP1, CLEC7A, SIRPA, TLR2 | 0.002 |
| GOTERM_BP_ALL | GO:0032943~mononuclear cell proliferation | 7 | CD86, CR1, LST1, LILRB2, TNFRSF1B, HLA-DRB1, CCR2 | 0.002 |
| GOTERM_BP_ALL | GO:0032760~positive regulation of tumor necrosis factor production | 5 | CLEC7A, CD36, LILRA2, CCR2, TLR2 | 0.002 |
| GOTERM_BP_ALL | GO:0002250~adaptive immune response | 10 | CD86, CR1, CLEC7A, SIGLEC10, HLA-DRA, LILRB2, TNFRSF1B, CTSS, HLA-DRB1, CCR2 | 0.002 |
| GOTERM_BP_ALL | GO:0051090~regulation of sequence-specific DNA binding transcription factor activity | 8 | HCK, CLEC7A, ITGB2, S100A12, CD36, S100A9, S100A8, TLR2 | 0.002 |
| GOTERM_BP_ALL | GO:0045621~positive regulation of lymphocyte differentiation | 5 | CD86, CR1, HLA-DRA, LILRB2, HLA-DRB1 | 0.002 |
| GOTERM_BP_ALL | GO:0051701~interaction with host | 6 | IFITM3, CD86, CR1, IFITM2, CCR5, HLA-DRB1 | 0.002 |
| GOTERM_BP_ALL | GO:0002443~leukocyte mediated immunity | 8 | FGR, CR1, CLEC7A, ITGB2, HLA-DRA, TNFRSF1B, HLA-DRB1, CCR2 | 0.003 |
| GOTERM_BP_ALL | GO:0046903~secretion | 11 | FGR, HCK, LRP1, VDR, ITGB2, TNFRSF1B, HLA-DRB1, S100A8, CCR2, ENG, TLR2 | 0.003 |
| GOTERM_BP_ALL | GO:0032649~regulation of interferon-gamma production | 5 | CR1, CLEC7A, SIRPA, HLA-DRB1, CCR2 | 0.003 |
| GOTERM_BP_ALL | GO:0008285~negative regulation of cell proliferation | 10 | CDKN1C, CD86, CSF1R, CR1, VDR, LST1, LILRB2, HLA-DRB1, ENG, TLR2 | 0.003 |
| GOTERM_BP_ALL | GO:0002218~activation of innate immune response | 6 | HCK, CLEC7A, LILRA2, CLEC4E, CTSS, TLR2 | 0.003 |
| GOTERM_BP_ALL | GO:0009611~response to wounding | 9 | SERPINB2, LRP1, CLEC7A, F13A1, CD36, RHOC, S100A8, CCR2, ENG | 0.003 |
| GOTERM_BP_ALL | GO:0000165~MAPK cascade | 10 | CSF1R, LRP1, CLEC7A, PROK2, SIRPA, S100A12, CD36, CCR5, HLA-DRB1, NAIP | 0.003 |
| GOTERM_BP_ALL | GO:0023014~signal transduction by protein phosphorylation | 10 | CSF1R, LRP1, CLEC7A, PROK2, SIRPA, S100A12, CD36, CCR5, HLA-DRB1, NAIP | 0.003 |
| GOTERM_BP_ALL | GO:0051049~regulation of transport | 15 | LRP1, ITGB2, LILRB2, LILRA2, TNFRSF1B, CTSS, FGR, HCK, CLEC7A, SIRPA, CD36, S100A8, HLA-DRB1, CCR2, TLR2 | 0.003 |
| GOTERM_BP_ALL | GO:0001934~positive regulation of protein phosphorylation | 11 | CD86, CSF1R, LRP1, CLEC7A, PECAM1, PROK2, S100A12, CD36, HLA-DRB1, NAIP, ENG | 0.004 |
| GOTERM_BP_ALL | GO:0009615~response to virus | 7 | IFITM3, FGR, IFITM2, ITGAX, RNASE2, POU2F2, TLR2 | 0.005 |
| GOTERM_BP_ALL | GO:0034113~heterotypic cell-cell adhesion | 4 | ITGB2, ITGAX, SIRPA, LILRB2 | 0.005 |
| GOTERM_BP_ALL | GO:0032623~interleukin-2 production | 4 | CD86, CR1, CLEC7A, CCR2 | 0.005 |
| GOTERM_BP_ALL | GO:0043410~positive regulation of MAPK cascade | 8 | CSF1R, LRP1, CLEC7A, PROK2, S100A12, CD36, HLA-DRB1, NAIP | 0.005 |
| GOTERM_BP_ALL | GO:0034341~response to interferon-gamma | 5 | IFITM3, HCK, IFITM2, SIRPA, TLR2 | 0.005 |
| GOTERM_BP_ALL | GO:0032663~regulation of interleukin-2 production | 4 | CD86, CR1, CLEC7A, CCR2 | 0.006 |
| GOTERM_BP_ALL | GO:0002705~positive regulation of leukocyte mediated immunity | 5 | FGR, CLEC7A, ITGB2, HLA-DRA, HLA-DRB1 | 0.006 |
| GOTERM_BP_ALL | GO:0043122~regulation of I-kappaB kinase/NF-kappaB signaling | 6 | CLEC7A, SIRPA, S100A12, CD36, RHOC, HLA-DRB1 | 0.007 |
| GOTERM_BP_ALL | GO:0045428~regulation of nitric oxide biosynthetic process | 4 | CLEC7A, ITGB2, SIRPA, CD36 | 0.007 |
| GOTERM_BP_ALL | GO:0046635~positive regulation of alpha-beta T cell activation | 4 | CD86, HLA-DRA, HLA-DRB1, CCR2 | 0.007 |
| GOTERM_BP_ALL | GO:0016045~detection of bacterium | 3 | HLA-DRB1, NAIP, TLR2 | 0.007 |
| GOTERM_BP_ALL | GO:0043408~regulation of MAPK cascade | 9 | CSF1R, LRP1, CLEC7A, PROK2, SIRPA, S100A12, CD36, HLA-DRB1, NAIP | 0.008 |
| GOTERM_BP_ALL | GO:0016192~vesicle-mediated transport | 13 | CD163, LRP1, ITGB2, FGR, HCK, CLEC7A, CXCR2, STAB1, PECAM1, SIRPA, CD36, CCR2, TLR2 | 0.008 |
| GOTERM_BP_ALL | GO:0042130~negative regulation of T cell proliferation | 4 | CD86, CR1, LILRB2, HLA-DRB1 | 0.008 |
| GOTERM_BP_ALL | GO:1902107~positive regulation of leukocyte differentiation | 5 | CD86, CR1, HLA-DRA, LILRB2, HLA-DRB1 | 0.008 |
| GOTERM_BP_ALL | GO:0050766~positive regulation of phagocytosis | 4 | LRP1, CLEC7A, SIRPA, CD36 | 0.008 |
| GOTERM_BP_ALL | GO:0045089~positive regulation of innate immune response | 6 | HCK, CLEC7A, LILRA2, CLEC4E, CTSS, TLR2 | 0.008 |
| GOTERM_BP_ALL | GO:0002292~T cell differentiation involved in immune response | 4 | CD86, HLA-DRA, CLEC4E, HLA-DRB1 | 0.009 |
| GOTERM_BP_ALL | GO:0002221~pattern recognition receptor signaling pathway | 5 | CLEC7A, LILRA2, CLEC4E, CTSS, TLR2 | 0.009 |
| GOTERM_BP_ALL | GO:0043299~leukocyte degranulation | 4 | FGR, HCK, ITGB2, CCR2 | 0.009 |
| GOTERM_BP_ALL | GO:0002706~regulation of lymphocyte mediated immunity | 5 | CR1, HLA-DRA, TNFRSF1B, HLA-DRB1, CCR2 | 0.01 |
| GOTERM_BP_ALL | GO:0006809~nitric oxide biosynthetic process | 4 | CLEC7A, ITGB2, SIRPA, CD36 | 0.01 |
| GOTERM_BP_ALL | GO:0043900~regulation of multi-organism process | 7 | IFITM3, CSF1R, IFITM2, CLEC7A, CD36, LILRA2, HLA-DRB1 | 0.01 |
| GOTERM_BP_ALL | GO:0002437~inflammatory response to antigenic stimulus | 4 | FGR, HCK, CXCR2, HLA-DRB1 | 0.01 |
| GOTERM_BP_ALL | GO:0044765~single-organism transport | 18 | CR1, LRP1, VDR, ITGB2, TNFRSF1B, FGR, HCK, CLEC7A, CXCR2, PECAM1, SIRPA, CD36, CCR5, S100A8, HLA-DRB1, CCR2, ENG, TLR2 | 0.01 |
| GOTERM_BP_ALL | GO:0007162~negative regulation of cell adhesion | 6 | CD86, CR1, LRP1, ZNF703, LILRB2, HLA-DRB1 | 0.01 |
| GOTERM_BP_ALL | GO:0002709~regulation of T cell mediated immunity | 4 | HLA-DRA, TNFRSF1B, HLA-DRB1, CCR2 | 0.01 |
| GOTERM_BP_ALL | GO:0002429~immune response-activating cell surface receptor signaling pathway | 6 | FGR, HCK, CR1, CLEC7A, LILRA2, HLA-DRB1 | 0.01 |
| GOTERM_BP_ALL | GO:0032940~secretion by cell | 9 | FGR, HCK, LRP1, ITGB2, TNFRSF1B, HLA-DRB1, CCR2, ENG, TLR2 | 0.01 |
| GOTERM_BP_ALL | GO:0050777~negative regulation of immune response | 5 | FGR, HCK, CR1, HLA-DRB1, CCR2 | 0.01 |
| GOTERM_BP_ALL | GO:0043123~positive regulation of I-kappaB kinase/NF-kappaB signaling | 5 | CLEC7A, S100A12, CD36, RHOC, HLA-DRB1 | 0.01 |
| GOTERM_BP_ALL | GO:0051046~regulation of secretion | 8 | FGR, LRP1, ITGB2, TNFRSF1B, HLA-DRB1, S100A8, CCR2, TLR2 | 0.01 |
| GOTERM_BP_ALL | GO:0002285~lymphocyte activation involved in immune response | 5 | CD86, CR1, HLA-DRA, CLEC4E, HLA-DRB1 | 0.01 |
| GOTERM_BP_ALL | GO:0002275~myeloid cell activation involved in immune response | 4 | FGR, ITGB2, LILRA2, CCR2 | 0.01 |
| GOTERM_BP_ALL | GO:0022408~negative regulation of cell-cell adhesion | 5 | CD86, CR1, ZNF703, LILRB2, HLA-DRB1 | 0.01 |
| GOTERM_BP_ALL | GO:0042098~T cell proliferation | 5 | CD86, CR1, LILRB2, HLA-DRB1, CCR2 | 0.01 |
| GOTERM_BP_ALL | GO:1903708~positive regulation of hemopoiesis | 5 | CD86, CR1, HLA-DRA, LILRB2, HLA-DRB1 | 0.02 |
| GOTERM_BP_ALL | GO:0042060~wound healing | 7 | SERPINB2, CLEC7A, F13A1, CD36, RHOC, S100A8, ENG | 0.02 |
| GOTERM_BP_ALL | GO:0002367~cytokine production involved in immune response | 4 | CLEC7A, CD36, TNFRSF1B, CCR2 | 0.02 |
| GOTERM_BP_ALL | GO:0002718~regulation of cytokine production involved in immune response | 4 | CLEC7A, CD36, TNFRSF1B, CCR2 | 0.02 |
| GOTERM_BP_ALL | GO:0046634~regulation of alpha-beta T cell activation | 4 | CD86, HLA-DRA, HLA-DRB1, CCR2 | 0.02 |
| GOTERM_BP_ALL | GO:0051051~negative regulation of transport | 7 | LRP1, SIRPA, LILRB2, CD36, TNFRSF1B, CCR2, TLR2 | 0.02 |
| GOTERM_BP_ALL | GO:0038094~Fc-gamma receptor signaling pathway | 3 | FGR, HCK, CLEC4E | 0.02 |
| GOTERM_BP_ALL | GO:0043372~positive regulation of CD4-positive, alpha-beta T cell differentiation | 3 | CD86, HLA-DRA, HLA-DRB1 | 0.02 |
| GOTERM_BP_ALL | GO:0018108~peptidyl-tyrosine phosphorylation | 6 | FGR, CSF1R, HCK, CLEC7A, PECAM1, CD36 | 0.02 |
| GOTERM_BP_ALL | GO:0006959~humoral immune response | 6 | CR1, S100A12, POU2F2, S100A9, HLA-DRB1, CCR2 | 0.02 |
| GOTERM_BP_ALL | GO:0032651~regulation of interleukin-1 beta production | 4 | CLEC7A, SIRPA, CD36, LILRA2 | 0.02 |
| GOTERM_BP_ALL | GO:0018212~peptidyl-tyrosine modification | 6 | FGR, CSF1R, HCK, CLEC7A, PECAM1, CD36 | 0.02 |
| GOTERM_BP_ALL | GO:0002862~negative regulation of inflammatory response to antigenic stimulus | 3 | FGR, HCK, HLA-DRB1 | 0.02 |
| GOTERM_BP_ALL | GO:0002456~T cell mediated immunity | 4 | HLA-DRA, TNFRSF1B, HLA-DRB1, CCR2 | 0.02 |
| GOTERM_BP_ALL | GO:0019882~antigen processing and presentation | 4 | HLA-DRA, LILRB2, CTSS, HLA-DRB1 | 0.02 |
| GOTERM_BP_ALL | GO:0002824~positive regulation of adaptive immune response based on somatic recombination of immune receptors built from immunoglobulin superfamily domains | 4 | CLEC7A, HLA-DRA, HLA-DRB1, CCR2 | 0.02 |
| GOTERM_BP_ALL | GO:1903426~regulation of reactive oxygen species biosynthetic process | 4 | CLEC7A, ITGB2, SIRPA, CD36 | 0.02 |
| GOTERM_BP_ALL | GO:0032609~interferon-gamma production | 4 | CR1, CLEC7A, HLA-DRB1, CCR2 | 0.02 |
| GOTERM_BP_ALL | GO:0002286~T cell activation involved in immune response | 4 | CD86, HLA-DRA, CLEC4E, HLA-DRB1 | 0.02 |
| GOTERM_BP_ALL | GO:0002460~adaptive immune response based on somatic recombination of immune receptors built from immunoglobulin superfamily domains | 6 | CR1, CLEC7A, HLA-DRA, TNFRSF1B, HLA-DRB1, CCR2 | 0.02 |
| GOTERM_BP_ALL | GO:0032743~positive regulation of interleukin-2 production | 3 | CD86, CLEC7A, CCR2 | 0.02 |
| GOTERM_BP_ALL | GO:0002821~positive regulation of adaptive immune response | 4 | CLEC7A, HLA-DRA, HLA-DRB1, CCR2 | 0.03 |
| GOTERM_BP_ALL | GO:0006810~transport | 23 | CD163, CR1, LRP1, VDR, ITGB2, LILRB2, LILRA2, TNFRSF1B, CTSS, FGR, HCK, CLEC7A, CXCR2, STAB1, PECAM1, SIRPA, CD36, CCR5, S100A8, HLA-DRB1, CCR2, ENG, TLR2 | 0.03 |
| GOTERM_BP_ALL | GO:0032102~negative regulation of response to external stimulus | 6 | FGR, HCK, SERPINB2, SIRPA, LILRA2, HLA-DRB1 | 0.03 |
| GOTERM_BP_ALL | GO:0045622~regulation of T-helper cell differentiation | 3 | CD86, HLA-DRA, HLA-DRB1 | 0.03 |
| GOTERM_BP_ALL | GO:2000516~positive regulation of CD4-positive, alpha-beta T cell activation | 3 | CD86, HLA-DRA, HLA-DRB1 | 0.03 |
| GOTERM_BP_ALL | GO:0019058~viral life cycle | 6 | IFITM3, CD86, CR1, IFITM2, CCR5, HLA-DRB1 | 0.03 |
| GOTERM_BP_ALL | GO:0050868~negative regulation of T cell activation | 4 | CD86, CR1, LILRB2, HLA-DRB1 | 0.03 |
| GOTERM_BP_ALL | GO:0032612~interleukin-1 production | 4 | CLEC7A, SIRPA, CD36, LILRA2 | 0.03 |
| GOTERM_BP_ALL | GO:1903530~regulation of secretion by cell | 7 | FGR, LRP1, ITGB2, TNFRSF1B, HLA-DRB1, CCR2, TLR2 | 0.03 |
| GOTERM_BP_ALL | GO:0032652~regulation of interleukin-1 production | 4 | CLEC7A, SIRPA, CD36, LILRA2 | 0.03 |
| GOTERM_BP_ALL | GO:1903409~reactive oxygen species biosynthetic process | 4 | CLEC7A, ITGB2, SIRPA, CD36 | 0.03 |
| GOTERM_BP_ALL | GO:0090322~regulation of superoxide metabolic process | 3 | CLEC7A, ITGB2, CD36 | 0.03 |
| GOTERM_BP_ALL | GO:0032735~positive regulation of interleukin-12 production | 3 | CLEC7A, CD36, TLR2 | 0.03 |
| GOTERM_BP_ALL | GO:0050730~regulation of peptidyl-tyrosine phosphorylation | 5 | CSF1R, CLEC7A, ITGB2, PECAM1, CD36 | 0.03 |
| GOTERM_BP_ALL | GO:0051234~establishment of localization | 23 | CD163, CR1, LRP1, VDR, ITGB2, LILRB2, LILRA2, TNFRSF1B, CTSS, FGR, HCK, CLEC7A, CXCR2, STAB1, PECAM1, SIRPA, CD36, CCR5, S100A8, HLA-DRB1, CCR2, ENG, TLR2 | 0.04 |
| GOTERM_BP_ALL | GO:0046596~regulation of viral entry into host cell | 3 | IFITM3, IFITM2, HLA-DRB1 | 0.04 |
| GOTERM_BP_ALL | GO:0051128~regulation of cellular component organization | 16 | CDKN1C, CSF1R, LRP1, LST1, ITGB2, LILRB2, RHOC, TNFRSF1B, FGR, HCK, CLEC7A, SIRPA, CD36, S100A9, S100A8, TLR2 | 0.04 |
| GOTERM_BP_ALL | GO:0002861~regulation of inflammatory response to antigenic stimulus | 3 | FGR, HCK, HLA-DRB1 | 0.04 |
| GOTERM_BP_ALL | GO:0051607~defense response to virus | 5 | IFITM3, IFITM2, ITGAX, RNASE2, TLR2 | 0.04 |
| GOTERM_BP_ALL | GO:0045429~positive regulation of nitric oxide biosynthetic process | 3 | CLEC7A, ITGB2, CD36 | 0.04 |
| GOTERM_BP_ALL | GO:0048585~negative regulation of response to stimulus | 12 | FGR, HCK, CR1, SERPINB2, LRP1, NAMPT, SIRPA, LILRA2, HLA-DRB1, NAIP, CCR2, ENG | 0.04 |
| GOTERM_BP_ALL | GO:0031348~negative regulation of defense response | 5 | FGR, HCK, CR1, SIRPA, HLA-DRB1 | 0.04 |
| GOTERM_BP_ALL | GO:1903038~negative regulation of leukocyte cell-cell adhesion | 4 | CD86, CR1, LILRB2, HLA-DRB1 | 0.04 |
| GOTERM_BP_ALL | GO:0046638~positive regulation of alpha-beta T cell differentiation | 3 | CD86, HLA-DRA, HLA-DRB1 | 0.04 |
| GOTERM_BP_ALL | GO:1904407~positive regulation of nitric oxide metabolic process | 3 | CLEC7A, ITGB2, CD36 | 0.04 |
| GOTERM_BP_ALL | GO:0043300~regulation of leukocyte degranulation | 3 | FGR, ITGB2, CCR2 | 0.04 |
| GOTERM_BP_ALL | GO:0043370~regulation of CD4-positive, alpha-beta T cell differentiation | 3 | CD86, HLA-DRA, HLA-DRB1 | 0.04 |
| GOTERM_BP_ALL | GO:0072593~reactive oxygen species metabolic process | 5 | CLEC7A, ITGB2, SIRPA, CD36, TLR2 | 0.04 |
|  |  |  |  |  |
| Annotation Cluster 5 | Enrichment Score: 3.6604588724274127 |  |  |  |
| Category | Term | Count | Genes | FDR |
| GOTERM_BP_ALL | GO:0002376~immune system process | 38 | CDKN1C, IFITM3, CD86, CSF1R, IFITM2, CSF3R, LRP1, LST1, ITGB2, LILRA2, CTSS, CLEC7A, NAMPT, CXCR2, ITGAX, S100A12, SIRPA, CD36, CCR5, CCR2, CR1, SIGLEC10, LILRB2, POU2F2, RNASE2, TNFRSF1B, FGR, HCK, MAFB, FCGR2A, PECAM1, HLA-DRA, CLEC4E, S100A9, S100A8, HLA-DRB1, NAIP, TLR2 | 8.70E-16 |
| GOTERM_BP_ALL | GO:0006952~defense response | 32 | IFITM3, CSF1R, IFITM2, CSF3R, ITGB2, LILRA2, CTSS, CLEC7A, CXCR2, STAB1, ITGAX, PROK2, S100A12, SIRPA, CD36, CCR5, CCR2, CD163, CR1, SIGLEC10, LILRB2, RNASE2, TNFRSF1B, NEAT1, FGR, HCK, CLEC4E, S100A9, S100A8, HLA-DRB1, NAIP, TLR2 | 8.70E-16 |
| GOTERM_BP_ALL | GO:0009605~response to external stimulus | 36 | IFITM3, CD86, CSF1R, IFITM2, CSF3R, LRP1, ITGB2, LILRA2, CLEC7A, NAMPT, CXCR2, STAB1, ITGAX, PROK2, S100A12, SIRPA, CD36, CCR5, CCR2, CR1, SERPINB2, VDR, LILRB2, POU2F2, RNASE2, TNFRSF1B, NEAT1, FGR, HCK, CLEC4E, S100A9, S100A8, HLA-DRB1, NAIP, ENG, TLR2 | 3.48E-15 |
| GOTERM_BP_ALL | GO:0006955~immune response | 31 | IFITM3, CD86, CSF1R, IFITM2, LRP1, LST1, ITGB2, LILRA2, CTSS, CLEC7A, CXCR2, S100A12, SIRPA, CD36, CCR5, CCR2, CR1, SIGLEC10, LILRB2, POU2F2, RNASE2, TNFRSF1B, FGR, HCK, HLA-DRA, CLEC4E, S100A9, S100A8, HLA-DRB1, NAIP, TLR2 | 5.27E-14 |
| GOTERM_BP_ALL | GO:0006954~inflammatory response | 22 | CSF1R, CD163, CR1, ITGB2, TNFRSF1B, NEAT1, FGR, HCK, CLEC7A, CXCR2, STAB1, PROK2, S100A12, SIRPA, CD36, CCR5, S100A9, S100A8, HLA-DRB1, NAIP, CCR2, TLR2 | 1.96E-13 |
| GOTERM_BP_ALL | GO:0006950~response to stress | 38 | IFITM3, CSF1R, IFITM2, CSF3R, LRP1, ITGB2, F13A1, LILRA2, CTSS, CLEC7A, NAMPT, CXCR2, STAB1, ITGAX, PROK2, S100A12, SIRPA, CD36, CCR5, CCR2, CD163, CR1, SERPINB2, SIGLEC10, LILRB2, RHOC, RNASE2, TNFRSF1B, NEAT1, FGR, HCK, CLEC4E, S100A9, S100A8, HLA-DRB1, NAIP, ENG, TLR2 | 2.02E-11 |
| GOTERM_BP_ALL | GO:0051707~response to other organism | 23 | IFITM3, CD86, IFITM2, LILRB2, POU2F2, LILRA2, RNASE2, TNFRSF1B, FGR, HCK, CLEC7A, STAB1, ITGAX, S100A12, SIRPA, CD36, CLEC4E, CCR5, S100A9, S100A8, HLA-DRB1, NAIP, TLR2 | 4.55E-11 |
| GOTERM_BP_ALL | GO:0043207~response to external biotic stimulus | 23 | IFITM3, CD86, IFITM2, LILRB2, POU2F2, LILRA2, RNASE2, TNFRSF1B, FGR, HCK, CLEC7A, STAB1, ITGAX, S100A12, SIRPA, CD36, CLEC4E, CCR5, S100A9, S100A8, HLA-DRB1, NAIP, TLR2 | 4.55E-11 |
| GOTERM_BP_ALL | GO:0009607~response to biotic stimulus | 23 | IFITM3, CD86, IFITM2, LILRB2, POU2F2, LILRA2, RNASE2, TNFRSF1B, FGR, HCK, CLEC7A, STAB1, ITGAX, S100A12, SIRPA, CD36, CLEC4E, CCR5, S100A9, S100A8, HLA-DRB1, NAIP, TLR2 | 9.74E-11 |
| GOTERM_BP_ALL | GO:0050793~regulation of developmental process | 30 | CDKN1C, CD86, CSF1R, CSF3R, LRP1, LST1, ITGB2, CLEC7A, ZNF703, NAMPT, CXCR2, STAB1, ITGAX, PROK2, CD36, CCR2, SRGN, CR1, VDR, LILRB2, RHOC, TNFRSF1B, FGR, HCK, MAFB, HLA-DRA, S100A9, HLA-DRB1, ENG, TLR2 | 1.48E-9 |
| GOTERM_BP_ALL | GO:0007166~cell surface receptor signaling pathway | 30 | CDKN1C, IFITM3, CD86, CSF1R, IFITM2, CSF3R, LRP1, ITGB2, MS4A7, LILRA2, CLEC7A, ZNF703, CXCR2, ITGAX, CD36, CCR5, CCR2, CR1, TNFRSF10C, LILRB2, TNFRSF1B, FGR, HCK, FCGR2A, PECAM1, CLEC4E, HLA-DRB1, NAIP, ENG, TLR2 | 2.20E-9 |
| GOTERM_BP_ALL | GO:0002252~immune effector process | 19 | IFITM3, CD86, CR1, IFITM2, LRP1, ITGB2, LILRA2, RNASE2, TNFRSF1B, FGR, HCK, CLEC7A, ITGAX, HLA-DRA, CD36, CLEC4E, HLA-DRB1, CCR2, TLR2 | 2.70E-9 |
| GOTERM_BP_ALL | GO:0032101~regulation of response to external stimulus | 19 | CSF1R, CR1, SERPINB2, LRP1, VDR, LILRA2, NEAT1, FGR, HCK, CLEC7A, CXCR2, S100A12, SIRPA, CD36, S100A9, S100A8, HLA-DRB1, CCR2, TLR2 | 3.48E-9 |
| GOTERM_BP_ALL | GO:0045087~innate immune response | 19 | IFITM3, CSF1R, CR1, IFITM2, SIGLEC10, LILRA2, RNASE2, CTSS, FGR, HCK, CLEC7A, S100A12, SIRPA, CD36, CLEC4E, S100A9, S100A8, NAIP, TLR2 | 6.04E-9 |
| GOTERM_BP_ALL | GO:0051704~multi-organism process | 28 | IFITM3, CD86, CSF1R, IFITM2, LILRA2, CLEC7A, NAMPT, STAB1, ITGAX, PROK2, S100A12, SIRPA, CD36, CCR5, CR1, VDR, LILRB2, POU2F2, RNASE2, TNFRSF1B, FGR, HCK, CLEC4E, S100A9, S100A8, HLA-DRB1, NAIP, TLR2 | 7.58E-9 |
| GOTERM_BP_ALL | GO:0051239~regulation of multicellular organismal process | 31 | CDKN1C, CD86, CSF1R, CSF3R, LRP1, ITGB2, LILRA2, CLEC7A, ZNF703, CXCR2, STAB1, ITGAX, PROK2, SIRPA, CD36, CCR2, SRGN, CR1, SERPINB2, VDR, LILRB2, POU2F2, TNFRSF1B, FGR, MAFB, HLA-DRA, CLEC4E, S100A9, HLA-DRB1, ENG, TLR2 | 1.23E-8 |
| GOTERM_BP_ALL | GO:0050896~response to stimulus | 49 | CDKN1C, IFITM3, CD86, CSF3R, IFITM2, LST1, ITGB2, MS4A7, F13A1, CTSS, NAMPT, STAB1, ITGAX, PROK2, SIRPA, CD36, CCR5, CCR2, SERPINB2, CR1, RHOC, RNASE2, TNFRSF1B, NEAT1, FGR, HCK, PECAM1, CLEC4E, S100A9, S100A8, NAIP, TLR2, ENG, CSF1R, LRP1, LILRA2, CLEC7A, ZNF703, CXCR2, S100A12, CD163, VDR, SIGLEC10, TNFRSF10C, LILRB2, POU2F2, FCGR2A, HLA-DRA, HLA-DRB1 | 1.31E-8 |
| GOTERM_BP_ALL | GO:0048584~positive regulation of response to stimulus | 27 | CDKN1C, CD86, CSF1R, LRP1, ITGB2, LILRA2, CTSS, CLEC7A, CXCR2, PROK2, S100A12, CD36, CCR2, CR1, VDR, RHOC, NEAT1, FGR, HCK, HLA-DRA, CLEC4E, S100A9, S100A8, HLA-DRB1, NAIP, ENG, TLR2 | 1.63E-8 |
| GOTERM_BP_ALL | GO:0051240~positive regulation of multicellular organismal process | 24 | CD86, CSF1R, CR1, LRP1, VDR, ITGB2, LILRB2, POU2F2, LILRA2, TNFRSF1B, FGR, CLEC7A, ZNF703, CXCR2, ITGAX, PROK2, HLA-DRA, CD36, CLEC4E, S100A9, HLA-DRB1, CCR2, ENG, TLR2 | 1.90E-8 |
| GOTERM_BP_ALL | GO:0007155~cell adhesion | 22 | CD86, CSF3R, CR1, LRP1, ITGB2, SIGLEC10, LILRB2, TNFRSF1B, HCK, CLEC7A, ZNF703, STAB1, ITGAX, PECAM1, HLA-DRA, SIRPA, CD36, S100A9, S100A8, HLA-DRB1, CCR2, ENG | 2.43E-8 |
| GOTERM_BP_ALL | GO:0022610~biological adhesion | 22 | CD86, CSF3R, CR1, LRP1, ITGB2, SIGLEC10, LILRB2, TNFRSF1B, HCK, CLEC7A, ZNF703, STAB1, ITGAX, PECAM1, HLA-DRA, SIRPA, CD36, S100A9, S100A8, HLA-DRB1, CCR2, ENG | 2.53E-8 |
| GOTERM_BP_ALL | GO:0009617~response to bacterium | 17 | CD86, LILRB2, LILRA2, TNFRSF1B, FGR, HCK, STAB1, S100A12, SIRPA, CD36, CLEC4E, CCR5, S100A9, S100A8, HLA-DRB1, NAIP, TLR2 | 2.81E-8 |
| GOTERM_BP_ALL | GO:0048583~regulation of response to stimulus | 34 | CDKN1C, CD86, CSF1R, LRP1, ITGB2, LILRA2, CTSS, CLEC7A, ZNF703, NAMPT, CXCR2, PROK2, S100A12, SIRPA, CD36, CCR2, CR1, SERPINB2, VDR, LILRB2, RHOC, TNFRSF1B, NEAT1, FGR, HCK, FCGR2A, HLA-DRA, CLEC4E, S100A9, S100A8, HLA-DRB1, NAIP, ENG, TLR2 | 2.96E-8 |
| GOTERM_BP_ALL | GO:0001816~cytokine production | 17 | CD86, SRGN, CSF1R, CR1, LRP1, LILRB2, POU2F2, LILRA2, TNFRSF1B, FGR, CLEC7A, SIRPA, CD36, CLEC4E, HLA-DRB1, CCR2, TLR2 | 3.46E-8 |
| GOTERM_BP_ALL | GO:0001817~regulation of cytokine production | 17 | CD86, SRGN, CSF1R, CR1, LRP1, LILRB2, POU2F2, LILRA2, TNFRSF1B, FGR, CLEC7A, SIRPA, CD36, CLEC4E, HLA-DRB1, CCR2, TLR2 | 4.56E-8 |
| GOTERM_BP_ALL | GO:0070887~cellular response to chemical stimulus | 29 | CDKN1C, IFITM3, CD86, CSF1R, IFITM2, CSF3R, LRP1, ITGB2, LILRA2, CTSS, CLEC7A, ZNF703, NAMPT, CXCR2, S100A12, SIRPA, CD36, CCR5, CCR2, VDR, LILRB2, TNFRSF1B, HCK, S100A9, S100A8, HLA-DRB1, NAIP, ENG, TLR2 | 5.90E-8 |
| GOTERM_BP_ALL | GO:0023052~signaling | 41 | CDKN1C, IFITM3, CD86, CSF1R, IFITM2, CSF3R, LRP1, ITGB2, MS4A7, LILRA2, CTSS, CLEC7A, ZNF703, NAMPT, CXCR2, STAB1, ITGAX, PROK2, S100A12, SIRPA, CD36, CCR5, CCR2, SRGN, CR1, VDR, TNFRSF10C, LILRB2, RHOC, TNFRSF1B, FGR, HCK, FCGR2A, PECAM1, CLEC4E, S100A9, S100A8, HLA-DRB1, NAIP, ENG, TLR2 | 8.64E-8 |
| GOTERM_BP_ALL | GO:0071310~cellular response to organic substance | 26 | CDKN1C, IFITM3, CD86, CSF1R, IFITM2, CSF3R, LRP1, ITGB2, LILRA2, CTSS, CLEC7A, ZNF703, NAMPT, CXCR2, SIRPA, CD36, CCR5, CCR2, VDR, LILRB2, TNFRSF1B, HCK, HLA-DRB1, NAIP, ENG, TLR2 | 1.06E-7 |
| GOTERM_BP_ALL | GO:0007154~cell communication | 41 | CDKN1C, IFITM3, CD86, CSF1R, IFITM2, CSF3R, LRP1, ITGB2, MS4A7, LILRA2, CTSS, CLEC7A, ZNF703, NAMPT, CXCR2, STAB1, ITGAX, PROK2, S100A12, SIRPA, CD36, CCR5, CCR2, SRGN, CR1, VDR, TNFRSF10C, LILRB2, RHOC, TNFRSF1B, FGR, HCK, FCGR2A, PECAM1, CLEC4E, S100A9, S100A8, HLA-DRB1, NAIP, ENG, TLR2 | 1.06E-7 |
| GOTERM_BP_ALL | GO:0031347~regulation of defense response | 16 | CR1, LILRA2, NEAT1, CTSS, FGR, HCK, CLEC7A, S100A12, SIRPA, CD36, CLEC4E, S100A9, S100A8, HLA-DRB1, CCR2, TLR2 | 1.18E-7 |
| GOTERM_BP_ALL | GO:0007165~signal transduction | 39 | CDKN1C, IFITM3, CD86, CSF1R, IFITM2, CSF3R, LRP1, ITGB2, MS4A7, LILRA2, CTSS, CLEC7A, ZNF703, NAMPT, CXCR2, ITGAX, PROK2, S100A12, SIRPA, CD36, CCR5, CCR2, CR1, VDR, TNFRSF10C, LILRB2, RHOC, TNFRSF1B, FGR, HCK, FCGR2A, PECAM1, CLEC4E, S100A9, S100A8, HLA-DRB1, NAIP, ENG, TLR2 | 1.84E-7 |
| GOTERM_BP_ALL | GO:0050776~regulation of immune response | 17 | CD86, CR1, ITGB2, LILRB2, LILRA2, TNFRSF1B, CTSS, FGR, HCK, FCGR2A, CLEC7A, HLA-DRA, CD36, CLEC4E, HLA-DRB1, CCR2, TLR2 | 1.88E-7 |
| GOTERM_BP_ALL | GO:0002684~positive regulation of immune system process | 18 | CD86, CSF1R, CR1, ITGB2, LILRB2, LILRA2, CTSS, FGR, HCK, CLEC7A, CXCR2, HLA-DRA, SIRPA, CD36, CLEC4E, HLA-DRB1, CCR2, TLR2 | 2.16E-7 |
| GOTERM_BP_ALL | GO:0044700~single organism signaling | 40 | CDKN1C, IFITM3, CD86, CSF1R, IFITM2, CSF3R, LRP1, ITGB2, MS4A7, LILRA2, CTSS, CLEC7A, ZNF703, NAMPT, CXCR2, STAB1, ITGAX, PROK2, S100A12, SIRPA, CD36, CCR5, CCR2, CR1, VDR, TNFRSF10C, LILRB2, RHOC, TNFRSF1B, FGR, HCK, FCGR2A, PECAM1, CLEC4E, S100A9, S100A8, HLA-DRB1, NAIP, ENG, TLR2 | 3.15E-7 |
| GOTERM_BP_ALL | GO:0010033~response to organic substance | 28 | CDKN1C, IFITM3, CD86, CSF1R, IFITM2, CSF3R, LRP1, ITGB2, LILRA2, CTSS, CLEC7A, ZNF703, NAMPT, CXCR2, SIRPA, CD36, CCR5, CCR2, VDR, LILRB2, TNFRSF1B, HCK, S100A9, S100A8, HLA-DRB1, NAIP, ENG, TLR2 | 6.04E-7 |
| GOTERM_BP_ALL | GO:0080134~regulation of response to stress | 20 | CR1, SERPINB2, LRP1, LILRA2, NEAT1, CTSS, FGR, HCK, CLEC7A, NAMPT, S100A12, SIRPA, CD36, CLEC4E, S100A9, S100A8, HLA-DRB1, NAIP, CCR2, TLR2 | 8.34E-7 |
| GOTERM_BP_ALL | GO:0050727~regulation of inflammatory response | 12 | FGR, HCK, CR1, CLEC7A, SIRPA, S100A12, NEAT1, S100A9, HLA-DRB1, S100A8, CCR2, TLR2 | 1.50E-6 |
| GOTERM_BP_ALL | GO:0044707~single-multicellular organism process | 38 | CDKN1C, CD86, CSF1R, CSF3R, LRP1, LST1, ITGB2, F13A1, LILRA2, CTSS, CLEC7A, ZNF703, NAMPT, CXCR2, STAB1, ITGAX, PROK2, SIRPA, CD36, CCR2, SRGN, CR1, SERPINB2, VDR, LILRB2, POU2F2, TNFRSF1B, FGR, MAFB, PECAM1, HLA-DRA, CLEC4E, S100A9, S100A8, HLA-DRB1, NAIP, ENG, TLR2 | 3.78E-6 |
| GOTERM_BP_ALL | GO:2000026~regulation of multicellular organismal development | 22 | CDKN1C, CD86, SRGN, CSF3R, CR1, LRP1, VDR, ITGB2, LILRB2, TNFRSF1B, MAFB, ZNF703, CXCR2, STAB1, ITGAX, PROK2, HLA-DRA, S100A9, HLA-DRB1, CCR2, ENG, TLR2 | 4.29E-6 |
| GOTERM_BP_ALL | GO:0042127~regulation of cell proliferation | 20 | CDKN1C, CD86, CSF1R, CR1, VDR, LST1, LILRB2, TNFRSF1B, NEAT1, HCK, CLEC7A, ZNF703, NAMPT, CXCR2, ITGAX, PROK2, HLA-DRB1, CCR2, ENG, TLR2 | 5.72E-6 |
| GOTERM_BP_ALL | GO:0008283~cell proliferation | 21 | CDKN1C, CD86, CSF1R, CR1, LRP1, VDR, LST1, LILRB2, TNFRSF1B, NEAT1, HCK, CLEC7A, ZNF703, NAMPT, CXCR2, ITGAX, PROK2, HLA-DRB1, CCR2, ENG, TLR2 | 5.76E-6 |
| GOTERM_BP_ALL | GO:0051094~positive regulation of developmental process | 18 | CD86, CR1, LRP1, VDR, ITGB2, LILRB2, TNFRSF1B, CLEC7A, ZNF703, CXCR2, ITGAX, HLA-DRA, CD36, S100A9, HLA-DRB1, CCR2, ENG, TLR2 | 1.02E-5 |
| GOTERM_BP_ALL | GO:0042221~response to chemical | 31 | CDKN1C, IFITM3, CD86, CSF1R, IFITM2, CSF3R, LRP1, ITGB2, LILRA2, CTSS, CLEC7A, ZNF703, NAMPT, CXCR2, PROK2, S100A12, SIRPA, CD36, CCR5, CCR2, VDR, LILRB2, RNASE2, TNFRSF1B, HCK, S100A9, S100A8, HLA-DRB1, NAIP, ENG, TLR2 | 1.19E-5 |
| GOTERM_BP_ALL | GO:0048518~positive regulation of biological process | 37 | CDKN1C, CD86, CSF1R, LRP1, ITGB2, LILRA2, CTSS, CLEC7A, ZNF703, NAMPT, CXCR2, ITGAX, PROK2, S100A12, SIRPA, CD36, CCR2, CR1, VDR, TNFRSF10C, LILRB2, RHOC, POU2F2, TNFRSF1B, NEAT1, FGR, HCK, MAFB, PECAM1, HLA-DRA, CLEC4E, S100A9, S100A8, HLA-DRB1, NAIP, ENG, TLR2 | 1.19E-5 |
| GOTERM_BP_ALL | GO:0048522~positive regulation of cellular process | 35 | CDKN1C, CD86, CSF1R, LRP1, ITGB2, LILRA2, CLEC7A, ZNF703, NAMPT, CXCR2, ITGAX, PROK2, S100A12, SIRPA, CD36, CCR2, CR1, VDR, TNFRSF10C, LILRB2, RHOC, POU2F2, TNFRSF1B, NEAT1, FGR, HCK, MAFB, PECAM1, HLA-DRA, S100A9, S100A8, HLA-DRB1, NAIP, ENG, TLR2 | 1.19E-5 |
| GOTERM_BP_ALL | GO:0045597~positive regulation of cell differentiation | 15 | CD86, CR1, LRP1, VDR, LILRB2, TNFRSF1B, CLEC7A, ZNF703, HLA-DRA, CD36, S100A9, HLA-DRB1, CCR2, ENG, TLR2 | 1.91E-5 |
| GOTERM_BP_ALL | GO:0009967~positive regulation of signal transduction | 18 | CDKN1C, CD86, CSF1R, LRP1, VDR, RHOC, FGR, CLEC7A, PROK2, S100A12, CD36, S100A9, S100A8, HLA-DRB1, NAIP, CCR2, ENG, TLR2 | 3.13E-5 |
| GOTERM_BP_ALL | GO:0044093~positive regulation of molecular function | 18 | CD86, CSF1R, CR1, LRP1, VDR, ITGB2, RHOC, CTSS, FGR, CLEC7A, PROK2, S100A12, CD36, S100A9, S100A8, HLA-DRB1, CCR2, TLR2 | 3.17E-5 |
| GOTERM_BP_ALL | GO:1902533~positive regulation of intracellular signal transduction | 15 | CD86, CSF1R, LRP1, RHOC, FGR, CLEC7A, PROK2, S100A12, CD36, S100A9, S100A8, HLA-DRB1, NAIP, ENG, TLR2 | 3.39E-5 |
| GOTERM_BP_ALL | GO:0030155~regulation of cell adhesion | 13 | CD86, CR1, LRP1, ITGB2, LILRB2, TNFRSF1B, ZNF703, HLA-DRA, SIRPA, CD36, HLA-DRB1, CCR2, ENG | 3.75E-5 |
| GOTERM_BP_ALL | GO:0065008~regulation of biological quality | 28 | CSF1R, LRP1, LST1, ITGB2, F13A1, CLEC7A, NAMPT, CXCR2, PROK2, S100A12, CD36, CCR5, CCR2, SRGN, SERPINB2, VDR, LILRB2, RHOC, TNFRSF1B, FGR, HCK, MAFB, PECAM1, S100A9, S100A8, HLA-DRB1, ENG, TLR2 | 3.75E-5 |
| GOTERM_BP_ALL | GO:0002697~regulation of immune effector process | 10 | FGR, CD86, CR1, CLEC7A, ITGB2, HLA-DRA, CD36, TNFRSF1B, HLA-DRB1, CCR2 | 5.07E-5 |
| GOTERM_BP_ALL | GO:0033993~response to lipid | 14 | CD86, VDR, LILRB2, LILRA2, TNFRSF1B, HCK, ZNF703, SIRPA, CD36, CCR5, S100A9, S100A8, ENG, TLR2 | 5.12E-5 |
| GOTERM_BP_ALL | GO:0051716~cellular response to stimulus | 39 | CDKN1C, IFITM3, CD86, CSF1R, IFITM2, CSF3R, LRP1, ITGB2, MS4A7, LILRA2, CTSS, CLEC7A, ZNF703, NAMPT, CXCR2, ITGAX, PROK2, S100A12, SIRPA, CD36, CCR5, CCR2, CR1, VDR, TNFRSF10C, LILRB2, RHOC, TNFRSF1B, FGR, HCK, FCGR2A, PECAM1, CLEC4E, S100A9, S100A8, HLA-DRB1, NAIP, ENG, TLR2 | 6.04E-5 |
| GOTERM_BP_ALL | GO:0048856~anatomical structure development | 34 | CDKN1C, CD86, CSF1R, CSF3R, LRP1, LST1, ITGB2, CLEC7A, ZNF703, CXCR2, STAB1, ITGAX, PROK2, CD36, CCR2, SRGN, CR1, VDR, LILRB2, RHOC, POU2F2, TNFRSF1B, FGR, HCK, MAFB, PECAM1, HLA-DRA, CLEC4E, S100A9, S100A8, HLA-DRB1, NAIP, ENG, TLR2 | 6.16E-5 |
| GOTERM_BP_ALL | GO:0006909~phagocytosis | 9 | FGR, HCK, LRP1, CLEC7A, ITGB2, PECAM1, SIRPA, CD36, TLR2 | 6.16E-5 |
| GOTERM_BP_ALL | GO:1901701~cellular response to oxygen-containing compound | 15 | CD86, LRP1, VDR, LILRB2, LILRA2, TNFRSF1B, HCK, CLEC7A, ZNF703, NAMPT, SIRPA, CD36, CCR5, HLA-DRB1, TLR2 | 6.16E-5 |
| GOTERM_BP_ALL | GO:0002521~leukocyte differentiation | 11 | CD86, CSF1R, CR1, MAFB, HLA-DRA, LILRB2, POU2F2, CLEC4E, HLA-DRB1, CCR2, TLR2 | 6.32E-5 |
| GOTERM_BP_ALL | GO:0048869~cellular developmental process | 29 | CDKN1C, CD86, CSF1R, CSF3R, LRP1, LST1, ITGB2, CLEC7A, ZNF703, NAMPT, CD36, CCR2, CR1, VDR, LILRB2, RHOC, POU2F2, TNFRSF1B, FGR, HCK, MAFB, PECAM1, HLA-DRA, CLEC4E, S100A9, S100A8, HLA-DRB1, ENG, TLR2 | 6.32E-5 |
| GOTERM_BP_ALL | GO:0030097~hemopoiesis | 13 | CDKN1C, CD86, CSF1R, CSF3R, CR1, LILRB2, POU2F2, MAFB, HLA-DRA, CLEC4E, HLA-DRB1, CCR2, TLR2 | 7.62E-5 |
| GOTERM_BP_ALL | GO:0044767~single-organism developmental process | 35 | CDKN1C, CD86, CSF1R, CSF3R, LRP1, LST1, ITGB2, CLEC7A, ZNF703, NAMPT, CXCR2, STAB1, ITGAX, PROK2, CD36, CCR2, SRGN, CR1, VDR, LILRB2, RHOC, POU2F2, TNFRSF1B, FGR, HCK, MAFB, PECAM1, HLA-DRA, CLEC4E, S100A9, S100A8, HLA-DRB1, NAIP, ENG, TLR2 | 8.51E-5 |
| GOTERM_BP_ALL | GO:0032879~regulation of localization | 23 | CSF1R, LRP1, ITGB2, LILRB2, RHOC, LILRA2, TNFRSF1B, CTSS, FGR, HCK, CLEC7A, ZNF703, CXCR2, ITGAX, PECAM1, SIRPA, CD36, CCR5, S100A8, HLA-DRB1, CCR2, ENG, TLR2 | 8.51E-5 |
| GOTERM_BP_ALL | GO:0065009~regulation of molecular function | 24 | CDKN1C, CD86, CSF1R, CR1, SERPINB2, LRP1, VDR, ITGB2, RHOC, CTSS, FGR, HCK, CLEC7A, PROK2, S100A12, SIRPA, CD36, S100A9, S100A8, HLA-DRB1, NAIP, CCR2, ENG, TLR2 | 9.26E-5 |
| GOTERM_BP_ALL | GO:0010647~positive regulation of cell communication | 18 | CDKN1C, CD86, CSF1R, LRP1, VDR, RHOC, FGR, CLEC7A, PROK2, S100A12, CD36, S100A9, S100A8, HLA-DRB1, NAIP, CCR2, ENG, TLR2 | 1.02E-4 |
| GOTERM_BP_ALL | GO:0023056~positive regulation of signaling | 18 | CDKN1C, CD86, CSF1R, LRP1, VDR, RHOC, FGR, CLEC7A, PROK2, S100A12, CD36, S100A9, S100A8, HLA-DRB1, NAIP, CCR2, ENG, TLR2 | 1.07E-4 |
| GOTERM_BP_ALL | GO:0048534~hematopoietic or lymphoid organ development | 13 | CDKN1C, CD86, CSF1R, CSF3R, CR1, LILRB2, POU2F2, MAFB, HLA-DRA, CLEC4E, HLA-DRB1, CCR2, TLR2 | 1.10E-4 |
| GOTERM_BP_ALL | GO:0032502~developmental process | 35 | CDKN1C, CD86, CSF1R, CSF3R, LRP1, LST1, ITGB2, CLEC7A, ZNF703, NAMPT, CXCR2, STAB1, ITGAX, PROK2, CD36, CCR2, SRGN, CR1, VDR, LILRB2, RHOC, POU2F2, TNFRSF1B, FGR, HCK, MAFB, PECAM1, HLA-DRA, CLEC4E, S100A9, S100A8, HLA-DRB1, NAIP, ENG, TLR2 | 1.17E-4 |
| GOTERM_BP_ALL | GO:0031325~positive regulation of cellular metabolic process | 25 | CDKN1C, CD86, CSF1R, LRP1, ITGB2, CLEC7A, NAMPT, PROK2, S100A12, CD36, CR1, VDR, LILRB2, POU2F2, TNFRSF1B, NEAT1, FGR, MAFB, PECAM1, S100A9, S100A8, HLA-DRB1, NAIP, ENG, TLR2 | 1.27E-4 |
| GOTERM_BP_ALL | GO:0009893~positive regulation of metabolic process | 26 | CDKN1C, CD86, CSF1R, LRP1, ITGB2, CLEC7A, NAMPT, ITGAX, PROK2, S100A12, CD36, CR1, VDR, LILRB2, POU2F2, TNFRSF1B, NEAT1, FGR, MAFB, PECAM1, S100A9, S100A8, HLA-DRB1, NAIP, ENG, TLR2 | 1.33E-4 |
| GOTERM_BP_ALL | GO:0032270~positive regulation of cellular protein metabolic process | 17 | CD86, CSF1R, CR1, LRP1, LILRB2, TNFRSF1B, NEAT1, CLEC7A, PECAM1, PROK2, S100A12, CD36, S100A9, S100A8, HLA-DRB1, NAIP, ENG | 1.39E-4 |
| GOTERM_BP_ALL | GO:0045595~regulation of cell differentiation | 18 | CDKN1C, CD86, CSF3R, CR1, LRP1, VDR, LILRB2, TNFRSF1B, MAFB, CLEC7A, ZNF703, HLA-DRA, CD36, S100A9, HLA-DRB1, CCR2, ENG, TLR2 | 1.39E-4 |
| GOTERM_BP_ALL | GO:0048731~system development | 30 | CDKN1C, CD86, CSF1R, CSF3R, LRP1, LST1, ITGB2, ZNF703, CXCR2, STAB1, ITGAX, PROK2, CCR2, SRGN, CR1, VDR, LILRB2, POU2F2, TNFRSF1B, FGR, MAFB, PECAM1, HLA-DRA, CLEC4E, S100A9, S100A8, HLA-DRB1, NAIP, ENG, TLR2 | 1.40E-4 |
| GOTERM_BP_ALL | GO:0006468~protein phosphorylation | 18 | CDKN1C, CD86, CSF1R, LRP1, ITGB2, FGR, HCK, CLEC7A, PECAM1, PROK2, S100A12, SIRPA, CD36, CCR5, HLA-DRB1, NAIP, ENG, TLR2 | 1.75E-4 |
| GOTERM_BP_ALL | GO:0002520~immune system development | 13 | CDKN1C, CD86, CSF1R, CSF3R, CR1, LILRB2, POU2F2, MAFB, HLA-DRA, CLEC4E, HLA-DRB1, CCR2, TLR2 | 1.75E-4 |
| GOTERM_BP_ALL | GO:0048519~negative regulation of biological process | 33 | CDKN1C, IFITM3, CD86, CSF1R, IFITM2, LRP1, LST1, ITGB2, LILRA2, ZNF703, NAMPT, CXCR2, STAB1, PROK2, SIRPA, CD36, CCR5, CCR2, SRGN, CR1, SERPINB2, VDR, LILRB2, TNFRSF1B, NEAT1, FGR, HCK, MAFB, MXD1, HLA-DRB1, NAIP, ENG, TLR2 | 1.75E-4 |
| GOTERM_BP_ALL | GO:0050794~regulation of cellular process | 48 | CDKN1C, IFITM3, CD86, CSF1R, IFITM2, CSF3R, LRP1, LST1, ITGB2, MS4A7, LILRA2, CTSS, CLEC7A, ZNF703, NAMPT, CXCR2, ITGAX, PROK2, S100A12, SIRPA, CD36, CCR5, CCR2, SRGN, CR1, SERPINB2, VDR, SECISBP2L, TNFRSF10C, LILRB2, RHOC, POU2F2, TNFRSF1B, NEAT1, FGR, HCK, MAFB, FCGR2A, PECAM1, HLA-DRA, CLEC4E, MXD1, S100A9, S100A8, HLA-DRB1, NAIP, ENG, TLR2 | 1.98E-4 |
| GOTERM_BP_ALL | GO:1901700~response to oxygen-containing compound | 17 | CD86, LRP1, VDR, LILRB2, LILRA2, TNFRSF1B, HCK, CLEC7A, ZNF703, NAMPT, SIRPA, CD36, CCR5, S100A9, S100A8, HLA-DRB1, TLR2 | 1.98E-4 |
| GOTERM_BP_ALL | GO:0051247~positive regulation of protein metabolic process | 17 | CD86, CSF1R, CR1, LRP1, LILRB2, TNFRSF1B, NEAT1, CLEC7A, PECAM1, PROK2, S100A12, CD36, S100A9, S100A8, HLA-DRB1, NAIP, ENG | 2.33E-4 |
| GOTERM_BP_ALL | GO:0032501~multicellular organismal process | 38 | CDKN1C, CD86, CSF1R, CSF3R, LRP1, LST1, ITGB2, F13A1, LILRA2, CTSS, CLEC7A, ZNF703, NAMPT, CXCR2, STAB1, ITGAX, PROK2, SIRPA, CD36, CCR2, SRGN, CR1, SERPINB2, VDR, LILRB2, POU2F2, TNFRSF1B, FGR, MAFB, PECAM1, HLA-DRA, CLEC4E, S100A9, S100A8, HLA-DRB1, NAIP, ENG, TLR2 | 2.34E-4 |
| GOTERM_BP_ALL | GO:0030154~cell differentiation | 27 | CDKN1C, CD86, CSF1R, CSF3R, LRP1, LST1, ITGB2, CLEC7A, ZNF703, CD36, CCR2, CR1, VDR, LILRB2, POU2F2, TNFRSF1B, FGR, HCK, MAFB, PECAM1, HLA-DRA, CLEC4E, S100A9, S100A8, HLA-DRB1, ENG, TLR2 | 2.42E-4 |
| GOTERM_BP_ALL | GO:0019220~regulation of phosphate metabolic process | 17 | CDKN1C, CD86, CSF1R, LRP1, ITGB2, LILRB2, FGR, CLEC7A, PECAM1, PROK2, S100A12, SIRPA, CD36, HLA-DRB1, NAIP, CCR2, ENG | 2.86E-4 |
| GOTERM_BP_ALL | GO:0051174~regulation of phosphorus metabolic process | 17 | CDKN1C, CD86, CSF1R, LRP1, ITGB2, LILRB2, FGR, CLEC7A, PECAM1, PROK2, S100A12, SIRPA, CD36, HLA-DRB1, NAIP, CCR2, ENG | 2.95E-4 |
| GOTERM_BP_ALL | GO:0050790~regulation of catalytic activity | 20 | CDKN1C, CD86, CSF1R, CR1, SERPINB2, LRP1, VDR, ITGB2, RHOC, FGR, CLEC7A, PROK2, S100A12, SIRPA, S100A9, S100A8, HLA-DRB1, NAIP, CCR2, ENG | 2.98E-4 |
| GOTERM_BP_ALL | GO:0050789~regulation of biological process | 49 | CDKN1C, IFITM3, CD86, CSF3R, IFITM2, LST1, ITGB2, MS4A7, CTSS, NAMPT, STAB1, ITGAX, PROK2, SIRPA, CD36, CCR5, CCR2, SRGN, SERPINB2, CR1, SECISBP2L, RHOC, TNFRSF1B, NEAT1, FGR, HCK, PECAM1, CLEC4E, S100A9, S100A8, NAIP, TLR2, ENG, CSF1R, LRP1, LILRA2, CLEC7A, ZNF703, CXCR2, S100A12, VDR, TNFRSF10C, LILRB2, POU2F2, FCGR2A, MAFB, HLA-DRA, MXD1, HLA-DRB1 | 3.02E-4 |
| GOTERM_BP_ALL | GO:0010604~positive regulation of macromolecule metabolic process | 24 | CDKN1C, CD86, CSF1R, CR1, LRP1, VDR, LILRB2, POU2F2, TNFRSF1B, NEAT1, MAFB, CLEC7A, NAMPT, ITGAX, PECAM1, PROK2, S100A12, CD36, S100A9, S100A8, HLA-DRB1, NAIP, ENG, TLR2 | 3.02E-4 |
| GOTERM_BP_ALL | GO:0032268~regulation of cellular protein metabolic process | 23 | CDKN1C, CD86, CSF1R, CR1, SERPINB2, LRP1, SECISBP2L, ITGB2, LILRB2, TNFRSF1B, NEAT1, FGR, CLEC7A, PECAM1, PROK2, S100A12, SIRPA, CD36, S100A9, S100A8, HLA-DRB1, NAIP, ENG | 3.09E-4 |
| GOTERM_BP_ALL | GO:0048523~negative regulation of cellular process | 31 | CDKN1C, IFITM3, CD86, CSF1R, IFITM2, LRP1, LST1, ITGB2, LILRA2, ZNF703, NAMPT, CXCR2, PROK2, SIRPA, CD36, CCR5, CCR2, CR1, SERPINB2, VDR, LILRB2, TNFRSF1B, NEAT1, FGR, HCK, MAFB, MXD1, HLA-DRB1, NAIP, ENG, TLR2 | 3.17E-4 |
| GOTERM_BP_ALL | GO:0001932~regulation of protein phosphorylation | 15 | CDKN1C, CD86, CSF1R, LRP1, ITGB2, FGR, CLEC7A, PECAM1, PROK2, S100A12, SIRPA, CD36, HLA-DRB1, NAIP, ENG | 4.81E-4 |
| GOTERM_BP_ALL | GO:0007275~multicellular organism development | 30 | CDKN1C, CD86, CSF1R, CSF3R, LRP1, LST1, ITGB2, ZNF703, CXCR2, STAB1, ITGAX, PROK2, CCR2, SRGN, CR1, VDR, LILRB2, POU2F2, TNFRSF1B, FGR, MAFB, PECAM1, HLA-DRA, CLEC4E, S100A9, S100A8, HLA-DRB1, NAIP, ENG, TLR2 | 4.81E-4 |
| GOTERM_BP_ALL | GO:0065007~biological regulation | 50 | CDKN1C, IFITM3, CD86, CSF3R, IFITM2, LST1, ITGB2, MS4A7, F13A1, CTSS, NAMPT, STAB1, ITGAX, PROK2, SIRPA, CD36, CCR5, CCR2, SRGN, SERPINB2, CR1, SECISBP2L, RHOC, TNFRSF1B, NEAT1, FGR, HCK, PECAM1, CLEC4E, S100A9, S100A8, NAIP, TLR2, ENG, CSF1R, LRP1, LILRA2, CLEC7A, ZNF703, CXCR2, S100A12, VDR, TNFRSF10C, LILRB2, POU2F2, FCGR2A, MAFB, HLA-DRA, MXD1, HLA-DRB1 | 5.45E-4 |
| GOTERM_BP_ALL | GO:0051050~positive regulation of transport | 12 | FGR, LRP1, CLEC7A, ITGB2, SIRPA, CD36, LILRA2, CTSS, HLA-DRB1, S100A8, CCR2, TLR2 | 5.88E-4 |
| GOTERM_BP_ALL | GO:0051246~regulation of protein metabolic process | 23 | CDKN1C, CD86, CSF1R, CR1, SERPINB2, LRP1, SECISBP2L, ITGB2, LILRB2, TNFRSF1B, NEAT1, FGR, CLEC7A, PECAM1, PROK2, S100A12, SIRPA, CD36, S100A9, S100A8, HLA-DRB1, NAIP, ENG | 6.13E-4 |
| GOTERM_BP_ALL | GO:0035556~intracellular signal transduction | 20 | CD86, CSF1R, LRP1, RHOC, TNFRSF1B, FGR, CLEC7A, CXCR2, PROK2, S100A12, SIRPA, CD36, CCR5, S100A9, S100A8, HLA-DRB1, NAIP, CCR2, ENG, TLR2 | 8.72E-4 |
| GOTERM_BP_ALL | GO:0010646~regulation of cell communication | 23 | CDKN1C, CD86, SRGN, CSF1R, LRP1, VDR, LILRB2, RHOC, LILRA2, FGR, CLEC7A, ZNF703, PROK2, S100A12, SIRPA, CD36, S100A9, S100A8, HLA-DRB1, NAIP, CCR2, ENG, TLR2 | 8.91E-4 |
| GOTERM_BP_ALL | GO:0045937~positive regulation of phosphate metabolic process | 13 | CD86, CSF1R, LRP1, LILRB2, FGR, CLEC7A, PECAM1, PROK2, S100A12, CD36, HLA-DRB1, NAIP, ENG | 9.45E-4 |
| GOTERM_BP_ALL | GO:0010562~positive regulation of phosphorus metabolic process | 13 | CD86, CSF1R, LRP1, LILRB2, FGR, CLEC7A, PECAM1, PROK2, S100A12, CD36, HLA-DRB1, NAIP, ENG | 9.45E-4 |
| GOTERM_BP_ALL | GO:0023051~regulation of signaling | 23 | CDKN1C, CD86, SRGN, CSF1R, LRP1, VDR, LILRB2, RHOC, LILRA2, FGR, CLEC7A, ZNF703, PROK2, S100A12, SIRPA, CD36, S100A9, S100A8, HLA-DRB1, NAIP, CCR2, ENG, TLR2 | 9.73E-4 |
| GOTERM_BP_ALL | GO:0042325~regulation of phosphorylation | 15 | CDKN1C, CD86, CSF1R, LRP1, ITGB2, FGR, CLEC7A, PECAM1, PROK2, S100A12, SIRPA, CD36, HLA-DRB1, NAIP, ENG | 0.001 |
| GOTERM_BP_ALL | GO:0009653~anatomical structure morphogenesis | 20 | CDKN1C, CSF1R, CSF3R, LRP1, VDR, LST1, ITGB2, RHOC, TNFRSF1B, FGR, HCK, MAFB, CXCR2, STAB1, ITGAX, PROK2, CD36, CCR2, ENG, TLR2 | 0.001 |
| GOTERM_BP_ALL | GO:0048513~animal organ development | 23 | CDKN1C, CD86, SRGN, CSF1R, CSF3R, CR1, LRP1, VDR, LILRB2, POU2F2, TNFRSF1B, FGR, MAFB, ZNF703, CXCR2, ITGAX, PECAM1, HLA-DRA, CLEC4E, HLA-DRB1, CCR2, ENG, TLR2 | 0.001 |
| GOTERM_BP_ALL | GO:0016310~phosphorylation | 18 | CDKN1C, CD86, CSF1R, LRP1, ITGB2, FGR, HCK, CLEC7A, PECAM1, PROK2, S100A12, SIRPA, CD36, CCR5, HLA-DRB1, NAIP, ENG, TLR2 | 0.001 |
| GOTERM_BP_ALL | GO:0060627~regulation of vesicle-mediated transport | 9 | FGR, HCK, LRP1, CLEC7A, ITGB2, SIRPA, CD36, CCR2, TLR2 | 0.001 |
| GOTERM_BP_ALL | GO:0009966~regulation of signal transduction | 21 | CDKN1C, CD86, CSF1R, LRP1, VDR, RHOC, LILRA2, FGR, CLEC7A, ZNF703, PROK2, S100A12, SIRPA, CD36, S100A9, S100A8, HLA-DRB1, NAIP, CCR2, ENG, TLR2 | 0.001 |
| GOTERM_BP_ALL | GO:1902531~regulation of intracellular signal transduction | 16 | CD86, CSF1R, LRP1, RHOC, FGR, CLEC7A, PROK2, S100A12, SIRPA, CD36, S100A9, S100A8, HLA-DRB1, NAIP, ENG, TLR2 | 0.001 |
| GOTERM_BP_ALL | GO:0031399~regulation of protein modification process | 16 | CDKN1C, CD86, CSF1R, LRP1, ITGB2, LILRB2, FGR, CLEC7A, PECAM1, PROK2, S100A12, SIRPA, CD36, HLA-DRB1, NAIP, ENG | 0.001 |
| GOTERM_BP_ALL | GO:0043085~positive regulation of catalytic activity | 13 | CD86, CSF1R, CR1, VDR, ITGB2, RHOC, FGR, CLEC7A, PROK2, S100A12, S100A9, S100A8, HLA-DRB1 | 0.002 |
| GOTERM_BP_ALL | GO:0042327~positive regulation of phosphorylation | 12 | FGR, CD86, CSF1R, LRP1, CLEC7A, PECAM1, PROK2, S100A12, CD36, HLA-DRB1, NAIP, ENG | 0.002 |
| GOTERM_BP_ALL | GO:0080090~regulation of primary metabolic process | 33 | CDKN1C, IFITM3, CD86, CSF1R, LRP1, ITGB2, CLEC7A, ZNF703, NAMPT, PROK2, S100A12, SIRPA, CD36, CCR2, CR1, SERPINB2, VDR, SECISBP2L, LILRB2, POU2F2, TNFRSF1B, NEAT1, FGR, HCK, MAFB, PECAM1, MXD1, S100A9, S100A8, HLA-DRB1, NAIP, ENG, TLR2 | 0.002 |
| GOTERM_BP_ALL | GO:0006796~phosphate-containing compound metabolic process | 21 | CDKN1C, CD86, CSF1R, LRP1, ITGB2, LILRB2, FGR, HCK, CLEC7A, NAMPT, PECAM1, PROK2, S100A12, SIRPA, CD36, CCR5, HLA-DRB1, NAIP, CCR2, ENG, TLR2 | 0.002 |
| GOTERM_BP_ALL | GO:0060255~regulation of macromolecule metabolic process | 33 | CDKN1C, IFITM3, CD86, CSF1R, LRP1, ITGB2, CLEC7A, ZNF703, NAMPT, ITGAX, PROK2, S100A12, SIRPA, CD36, CR1, SERPINB2, VDR, SECISBP2L, LILRB2, POU2F2, TNFRSF1B, NEAT1, FGR, HCK, MAFB, PECAM1, MXD1, S100A9, S100A8, HLA-DRB1, NAIP, ENG, TLR2 | 0.002 |
| GOTERM_BP_ALL | GO:0031323~regulation of cellular metabolic process | 33 | CDKN1C, IFITM3, CD86, CSF1R, LRP1, ITGB2, CLEC7A, ZNF703, NAMPT, PROK2, S100A12, SIRPA, CD36, CCR2, CR1, SERPINB2, VDR, SECISBP2L, LILRB2, POU2F2, TNFRSF1B, NEAT1, FGR, HCK, MAFB, PECAM1, MXD1, S100A9, S100A8, HLA-DRB1, NAIP, ENG, TLR2 | 0.003 |
| GOTERM_BP_ALL | GO:0006793~phosphorus metabolic process | 21 | CDKN1C, CD86, CSF1R, LRP1, ITGB2, LILRB2, FGR, HCK, CLEC7A, NAMPT, PECAM1, PROK2, S100A12, SIRPA, CD36, CCR5, HLA-DRB1, NAIP, CCR2, ENG, TLR2 | 0.003 |
| GOTERM_BP_ALL | GO:0008285~negative regulation of cell proliferation | 10 | CDKN1C, CD86, CSF1R, CR1, VDR, LST1, LILRB2, HLA-DRB1, ENG, TLR2 | 0.003 |
| GOTERM_BP_ALL | GO:0000165~MAPK cascade | 10 | CSF1R, LRP1, CLEC7A, PROK2, SIRPA, S100A12, CD36, CCR5, HLA-DRB1, NAIP | 0.003 |
| GOTERM_BP_ALL | GO:0023014~signal transduction by protein phosphorylation | 10 | CSF1R, LRP1, CLEC7A, PROK2, SIRPA, S100A12, CD36, CCR5, HLA-DRB1, NAIP | 0.003 |
| GOTERM_BP_ALL | GO:0051049~regulation of transport | 15 | LRP1, ITGB2, LILRB2, LILRA2, TNFRSF1B, CTSS, FGR, HCK, CLEC7A, SIRPA, CD36, S100A8, HLA-DRB1, CCR2, TLR2 | 0.003 |
| GOTERM_BP_ALL | GO:0001934~positive regulation of protein phosphorylation | 11 | CD86, CSF1R, LRP1, CLEC7A, PECAM1, PROK2, S100A12, CD36, HLA-DRB1, NAIP, ENG | 0.004 |
| GOTERM_BP_ALL | GO:0019222~regulation of metabolic process | 34 | CDKN1C, IFITM3, CD86, CSF1R, LRP1, ITGB2, CLEC7A, ZNF703, NAMPT, ITGAX, PROK2, S100A12, SIRPA, CD36, CCR2, CR1, SERPINB2, VDR, SECISBP2L, LILRB2, POU2F2, TNFRSF1B, NEAT1, FGR, HCK, MAFB, PECAM1, MXD1, S100A9, S100A8, HLA-DRB1, NAIP, ENG, TLR2 | 0.004 |
| GOTERM_BP_ALL | GO:0051179~localization | 31 | CSF1R, CSF3R, LRP1, ITGB2, LILRA2, CTSS, CLEC7A, ZNF703, CXCR2, STAB1, ITGAX, S100A12, SIRPA, CD36, CCR5, CCR2, SRGN, CD163, CR1, VDR, LILRB2, RHOC, TNFRSF1B, FGR, HCK, PECAM1, S100A9, S100A8, HLA-DRB1, ENG, TLR2 | 0.004 |
| GOTERM_BP_ALL | GO:0043410~positive regulation of MAPK cascade | 8 | CSF1R, LRP1, CLEC7A, PROK2, S100A12, CD36, HLA-DRB1, NAIP | 0.005 |
| GOTERM_BP_ALL | GO:0031401~positive regulation of protein modification process | 12 | CD86, CSF1R, LRP1, CLEC7A, PECAM1, PROK2, S100A12, LILRB2, CD36, HLA-DRB1, NAIP, ENG | 0.005 |
| GOTERM_BP_ALL | GO:0044267~cellular protein metabolic process | 28 | CDKN1C, CD86, CSF1R, LRP1, ITGB2, F13A1, CTSS, CLEC7A, PROK2, S100A12, SIRPA, CD36, CCR5, CR1, SERPINB2, SECISBP2L, LILRB2, TNFRSF1B, NEAT1, FGR, HCK, PECAM1, S100A9, S100A8, HLA-DRB1, NAIP, ENG, TLR2 | 0.007 |
| GOTERM_BP_ALL | GO:0043408~regulation of MAPK cascade | 9 | CSF1R, LRP1, CLEC7A, PROK2, SIRPA, S100A12, CD36, HLA-DRB1, NAIP | 0.008 |
| GOTERM_BP_ALL | GO:1902578~single-organism localization | 19 | SRGN, CR1, LRP1, VDR, ITGB2, TNFRSF1B, FGR, HCK, CLEC7A, CXCR2, PECAM1, SIRPA, CD36, CCR5, S100A8, HLA-DRB1, CCR2, ENG, TLR2 | 0.008 |
| GOTERM_BP_ALL | GO:0043900~regulation of multi-organism process | 7 | IFITM3, CSF1R, IFITM2, CLEC7A, CD36, LILRA2, HLA-DRB1 | 0.01 |
| GOTERM_BP_ALL | GO:0010468~regulation of gene expression | 26 | CDKN1C, IFITM3, CD86, LRP1, ITGB2, CLEC7A, ZNF703, NAMPT, ITGAX, S100A12, SIRPA, CD36, CR1, VDR, SECISBP2L, POU2F2, TNFRSF1B, NEAT1, HCK, MAFB, MXD1, S100A9, S100A8, HLA-DRB1, ENG, TLR2 | 0.01 |
| GOTERM_BP_ALL | GO:0006464~cellular protein modification process | 22 | CDKN1C, CD86, CSF1R, LRP1, ITGB2, F13A1, LILRB2, FGR, HCK, CLEC7A, PECAM1, PROK2, S100A12, SIRPA, CD36, CCR5, S100A9, S100A8, HLA-DRB1, NAIP, ENG, TLR2 | 0.01 |
| GOTERM_BP_ALL | GO:0036211~protein modification process | 22 | CDKN1C, CD86, CSF1R, LRP1, ITGB2, F13A1, LILRB2, FGR, HCK, CLEC7A, PECAM1, PROK2, S100A12, SIRPA, CD36, CCR5, S100A9, S100A8, HLA-DRB1, NAIP, ENG, TLR2 | 0.01 |
| GOTERM_BP_ALL | GO:0044763~single-organism cellular process | 44 | CDKN1C, CD86, CSF1R, CSF3R, LRP1, LST1, ITGB2, LILRA2, CTSS, CLEC7A, ZNF703, NAMPT, CXCR2, STAB1, ITGAX, PROK2, S100A12, SIRPA, CD36, CCR5, CCR2, SRGN, CR1, SERPINB2, VDR, TNFRSF10C, LILRB2, RHOC, POU2F2, TNFRSF1B, NEAT1, FGR, HCK, MAFB, VNN2, PECAM1, HLA-DRA, CLEC4E, S100A9, S100A8, HLA-DRB1, NAIP, ENG, TLR2 | 0.01 |
| GOTERM_BP_ALL | GO:0044765~single-organism transport | 18 | CR1, LRP1, VDR, ITGB2, TNFRSF1B, FGR, HCK, CLEC7A, CXCR2, PECAM1, SIRPA, CD36, CCR5, S100A8, HLA-DRB1, CCR2, ENG, TLR2 | 0.01 |
| GOTERM_BP_ALL | GO:0019538~protein metabolic process | 29 | CDKN1C, CD86, CSF1R, LRP1, ITGB2, F13A1, CTSS, CLEC7A, PROK2, S100A12, SIRPA, CD36, CCR5, SRGN, CR1, SERPINB2, SECISBP2L, LILRB2, TNFRSF1B, NEAT1, FGR, HCK, PECAM1, S100A9, S100A8, HLA-DRB1, NAIP, ENG, TLR2 | 0.02 |
| GOTERM_BP_ALL | GO:0043412~macromolecule modification | 22 | CDKN1C, CD86, CSF1R, LRP1, ITGB2, F13A1, LILRB2, FGR, HCK, CLEC7A, PECAM1, PROK2, S100A12, SIRPA, CD36, CCR5, S100A9, S100A8, HLA-DRB1, NAIP, ENG, TLR2 | 0.02 |
| GOTERM_BP_ALL | GO:2001141~regulation of RNA biosynthetic process | 20 | CDKN1C, IFITM3, CD86, VDR, ITGB2, POU2F2, TNFRSF1B, HCK, MAFB, CLEC7A, ZNF703, NAMPT, S100A12, CD36, MXD1, S100A9, S100A8, HLA-DRB1, ENG, TLR2 | 0.02 |
| GOTERM_BP_ALL | GO:0018108~peptidyl-tyrosine phosphorylation | 6 | FGR, CSF1R, HCK, CLEC7A, PECAM1, CD36 | 0.02 |
| GOTERM_BP_ALL | GO:0018212~peptidyl-tyrosine modification | 6 | FGR, CSF1R, HCK, CLEC7A, PECAM1, CD36 | 0.02 |
| GOTERM_BP_ALL | GO:0031326~regulation of cellular biosynthetic process | 24 | CDKN1C, IFITM3, CD86, VDR, SECISBP2L, ITGB2, POU2F2, TNFRSF1B, NEAT1, HCK, MAFB, CLEC7A, ZNF703, NAMPT, S100A12, SIRPA, CD36, MXD1, S100A9, S100A8, HLA-DRB1, CCR2, ENG, TLR2 | 0.02 |
| GOTERM_BP_ALL | GO:0006810~transport | 23 | CD163, CR1, LRP1, VDR, ITGB2, LILRB2, LILRA2, TNFRSF1B, CTSS, FGR, HCK, CLEC7A, CXCR2, STAB1, PECAM1, SIRPA, CD36, CCR5, S100A8, HLA-DRB1, CCR2, ENG, TLR2 | 0.03 |
| GOTERM_BP_ALL | GO:0009889~regulation of biosynthetic process | 24 | CDKN1C, IFITM3, CD86, VDR, SECISBP2L, ITGB2, POU2F2, TNFRSF1B, NEAT1, HCK, MAFB, CLEC7A, ZNF703, NAMPT, S100A12, SIRPA, CD36, MXD1, S100A9, S100A8, HLA-DRB1, CCR2, ENG, TLR2 | 0.03 |
| GOTERM_BP_ALL | GO:0044710~single-organism metabolic process | 21 | CSF1R, LRP1, VDR, ITGB2, CTSS, FGR, HCK, VNN2, CLEC7A, NAMPT, CXCR2, PROK2, S100A12, SIRPA, CD36, CCR5, HLA-DRB1, NAIP, CCR2, ENG, TLR2 | 0.03 |
| GOTERM_BP_ALL | GO:0044699~single-organism process | 49 | CDKN1C, IFITM3, CD86, CSF3R, IFITM2, LST1, ITGB2, MS4A7, F13A1, CTSS, NAMPT, STAB1, ITGAX, PROK2, SIRPA, CD36, CCR5, CCR2, SRGN, SERPINB2, CR1, RHOC, TNFRSF1B, NEAT1, FGR, HCK, PECAM1, CLEC4E, S100A9, S100A8, NAIP, TLR2, ENG, CSF1R, LRP1, LILRA2, CLEC7A, ZNF703, CXCR2, S100A12, VDR, TNFRSF10C, LILRB2, POU2F2, FCGR2A, MAFB, VNN2, HLA-DRA, HLA-DRB1 | 0.03 |
| GOTERM_BP_ALL | GO:0010628~positive regulation of gene expression | 14 | CDKN1C, CD86, CR1, VDR, POU2F2, NEAT1, MAFB, CLEC7A, NAMPT, ITGAX, CD36, HLA-DRB1, ENG, TLR2 | 0.03 |
| GOTERM_BP_ALL | GO:0051252~regulation of RNA metabolic process | 20 | CDKN1C, IFITM3, CD86, VDR, ITGB2, POU2F2, TNFRSF1B, HCK, MAFB, CLEC7A, ZNF703, NAMPT, S100A12, CD36, MXD1, S100A9, S100A8, HLA-DRB1, ENG, TLR2 | 0.03 |
| GOTERM_BP_ALL | GO:0050730~regulation of peptidyl-tyrosine phosphorylation | 5 | CSF1R, CLEC7A, ITGB2, PECAM1, CD36 | 0.03 |
| GOTERM_BP_ALL | GO:0051171~regulation of nitrogen compound metabolic process | 24 | CDKN1C, IFITM3, CD86, VDR, SECISBP2L, ITGB2, POU2F2, TNFRSF1B, NEAT1, HCK, MAFB, CLEC7A, ZNF703, NAMPT, S100A12, SIRPA, CD36, MXD1, S100A9, S100A8, HLA-DRB1, CCR2, ENG, TLR2 | 0.03 |
| GOTERM_BP_ALL | GO:0051234~establishment of localization | 23 | CD163, CR1, LRP1, VDR, ITGB2, LILRB2, LILRA2, TNFRSF1B, CTSS, FGR, HCK, CLEC7A, CXCR2, STAB1, PECAM1, SIRPA, CD36, CCR5, S100A8, HLA-DRB1, CCR2, ENG, TLR2 | 0.04 |
| GOTERM_BP_ALL | GO:0051128~regulation of cellular component organization | 16 | CDKN1C, CSF1R, LRP1, LST1, ITGB2, LILRB2, RHOC, TNFRSF1B, FGR, HCK, CLEC7A, SIRPA, CD36, S100A9, S100A8, TLR2 | 0.04 |
| GOTERM_BP_ALL | GO:0019219~regulation of nucleobase-containing compound metabolic process | 21 | CDKN1C, IFITM3, CD86, VDR, ITGB2, POU2F2, TNFRSF1B, HCK, MAFB, CLEC7A, ZNF703, NAMPT, S100A12, CD36, MXD1, S100A9, S100A8, HLA-DRB1, CCR2, ENG, TLR2 | 0.04 |
| GOTERM_BP_ALL | GO:0030225~macrophage differentiation | 3 | CSF1R, HLA-DRB1, TLR2 | 0.04 |
|  |  |  |  |  |

| Annotation Cluster 1 | Enrichment Score: 2.4601174713788336 |  |  |  |
| --- | --- | --- | --- | --- |
| Category | Term | Count | Genes | FDR |
| KEGG_PATHWAY | hsa05152:Tuberculosis | 11 | CR1, FCGR2A, CLEC7A, VDR, ITGB2, ITGAX, HLA-DRA, CLEC4E, CTSS, HLA-DRB1, TLR2 | 2.72E-7 |
| KEGG_PATHWAY | hsa04145:Phagosome | 8 | FCGR2A, CLEC7A, ITGB2, HLA-DRA, CD36, CTSS, HLA-DRB1, TLR2 | 9.85E-5 |
| KEGG_PATHWAY | hsa05140:Leishmaniasis | 6 | CR1, FCGR2A, ITGB2, HLA-DRA, HLA-DRB1, TLR2 | 4.03E-4 |
| KEGG_PATHWAY | hsa05323:Rheumatoid arthritis | 5 | CD86, ITGB2, HLA-DRA, HLA-DRB1, TLR2 | 0.007 |
| KEGG_PATHWAY | hsa05134:Legionellosis | 4 | CR1, ITGB2, NAIP, TLR2 | 0.02 |
| KEGG_PATHWAY | hsa05416:Viral myocarditis | 4 | CD86, ITGB2, HLA-DRA, HLA-DRB1 | 0.02 |
| KEGG_PATHWAY | hsa04514:Cell adhesion molecules | 5 | CD86, ITGB2, PECAM1, HLA-DRA, HLA-DRB1 | 0.03 |

## Supplementary Table 4A. Demographic and clinical characteristics in the participants receiving monocytic subtyping by flow cytometry

| Characteristics | Schizophrenia (n=29) | Healthy Controls  (n=27) | Z/X^2^ | *p*-value |
| --- | --- | --- | --- | --- |
| Age (years) | 24.90±5.70 | 25.41±3.83 | -0.387 | 0.699 |
| Male/female | 12/17 | 18/9 | 3.595 | 0.058 |
| Education (years)  Smoker/Non-smoker  Illness duration (months)  PANSS total  P subscore  N subscore  G subscore | 12.86±2.33  3/26  15.17±11.73  77.48±11.58  21.59±5.99  17.14±6.28  38.76±7.61 | 13.78±1.50  4/23  NA  NA  NA  NA  NA | -1.945  0.010  NA  NA  NA  NA  NA | 0.052  0.919  NA  NA  NA  NA  NA |

*PANSS* Positive and Negative Syndrome Scale.

## Supplementary Table 4B. Distribution of antipsychotics at the time of enrollment in the patients receiving monocytic subtyping by flow cytometry

| **Antipsychotics** | **Number of patients (n=29)** |
| --- | --- |
| Drug-naïve | 4 |
| Aripiprazole | 4 |
| Olanzapine | 3 |
| Olanzapine + Haloperidol injection | 1 |
| Olanzapine + Risperidone | 1 |
| Haloperidol injection | 1 |
| Risperidone | 12 |
| Paliperidone | 3 |
| Chlorpromazine equivalents were 236.00±104.88 mg/day. | |

## Supplementary Table 5. Comparations of the absolute counts of total blood monocytes

## and monocytic subsets between FES patients and HCs

| **Variables ^a^** | **FES (*n*=29)** | **HC (*n*=27)** | **F** | ***p*-value** |
| --- | --- | --- | --- | --- |
| Absolute counts of total blood monocytes (10^9^/L)  Percentage of total blood monocytes (%)  Absolute counts of classical monocytes (10^7^/L)  Absolute counts of intermediate monocytes (10^7^/L)  Absolute counts of nonclassical monocytes (10^7^/L) | 0.37±0.06  5.55±1.15  29.88±4.94  1.36±0.84  2.00±1.19 | 0.34±0.10  5.10±1.19  27.33±7.99  1.14±0.64  2.57±1.33 | 1.629  1.728  2.386  1.009  3.130 | 0.207  0.194  0.129  0.320  0.083 |

^a^Analysis of covariance with age and sex as covariates.

## Supplementary Table 6A. Demographic and clinical characteristics in participants undergoing brain imaging

| Characteristics | Schizophrenia  (n=60) | Healthy Controls  (n=54) | Z/X^2^ | *p*-value |
| --- | --- | --- | --- | --- |
| Age (years) | 30.47±8.28 | 32.69±9.33 | -1.037 | 0.300 |
| Male/female | 25/35 | 31/23 | 2.818 | 0.093 |
| Education (years)  Smoker/Non-smoker  Illness duration (months)  PANSS  Total score  P subscore  N subscore  G subscore | 12.73±3.44  5/55  12.04±12.39  74.26±12.54  21.47±4.39  16.48±5.70  36.33±7.07 | 13.78±2.50  9/45  NA  NA  NA  NA  NA | -1.845  1.832  NA  NA  NA  NA  NA | 0.065  0.176  NA  NA  NA  NA  NA |

*PANSS* Positive and Negative Syndrome Scale.

## Supplementary Table 6B. Distribution of antipsychotics at the time of enrollment in patients undergoing brain imaging

| **Antipsychotics** | **Number of patients (n=60)** |
| --- | --- |
| Drug-naïve | 10 |
| Aripiprazole | 4 |
| Olanzapine | 7 |
| Olanzapine + Aripiprazole | 1 |
| Olanzapine + Haloperidol injection | 2 |
| Olanzapine + Risperidone | 1 |
| Haloperidol injection | 1 |
| Risperidone | 24 |
| Risperidone + Haloperidol injection | 8 |
| Paliperidone | 2 |
| Chlorpromazine equivalents were 276.00±167.97 mg/day. | |

## Supplementary Table 7. Brain regions with significant differences in cortical thickness (mm) between FES patients and HCs

| **Cortical regions ^a^** | **FES (*n*=60)** | **HC (*n*=54)** | **F** | ***p*-value** | **FDR** |
| --- | --- | --- | --- | --- | --- |
| Supramarginal gyrus  Inferior parietal cortex  Superior parietal gyrus  Lateral occipital cortex  Inferior temporal gyrus  Pericalcarine  Precuneus  Fusiform gyrus  Lingual gyrus  Superior temporal gyrus | 2.56±0.12  2.49±0.14  2.23±0.16  2.21±0.12  2.85±0.11  1.65±0.14  2.44±0.10  2.83±0.10  2.06±0.12  2.87±0.13 | 2.67±0.11  2.60±0.10  2.35±0.10  2.31±0.11  2.91±0.11  1.58±0.13  2.50±0.11  2.88±0.10  2.00±0.12  2.91±0.13 | 28.012  24.920  20.380  16.914  8.492  8.032  8.115  6.983  7.358  6.579 | 6.218×10^-7^  2.257×10^-6^  1.601×10^-5^  7.571×10^-5^  0.004  0.005  0.005  0.009  0.008  0.012 | **2.114×10^-5***^**  **3.837×10^-5***^**  **1.814×10^-4***^**  **6.435×10^-4***^**  **0.025****^*^**  **0.025^*^**  **0.025^*^**  **0.034^*^**  **0.034^*^**  **0.041^*^** |

^a^Analysis of covariance with age and sex as covariates. ^*^FDR < 0.05; ^***^FDR < 0.001

# Supplementary figures and figure legends

## Supplementary Figure 1.

**A**
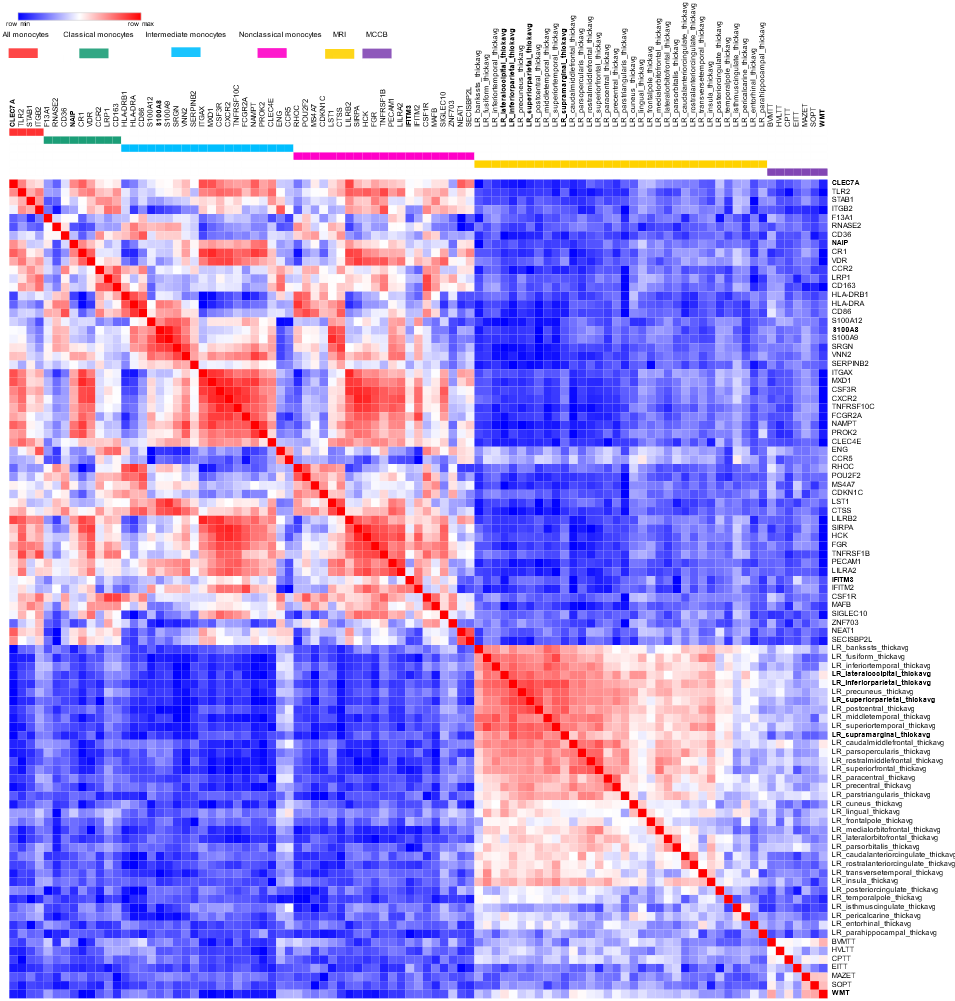


**B**


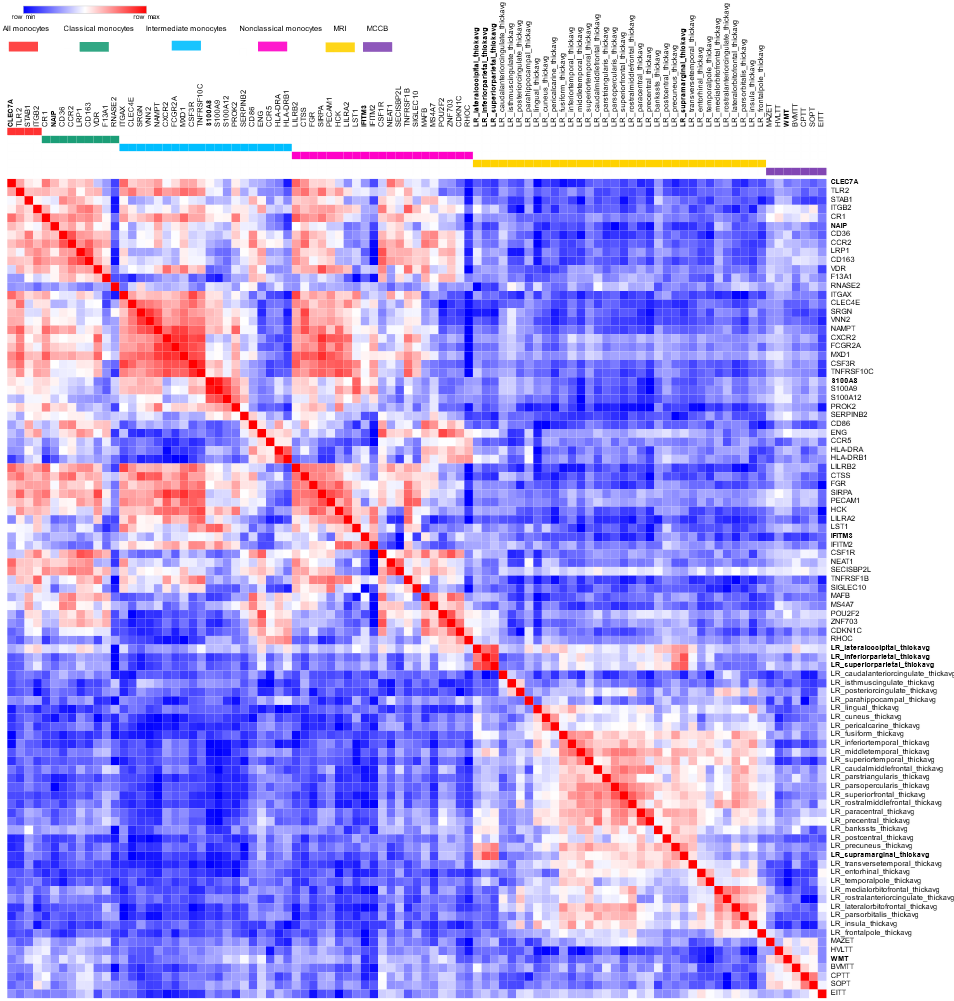


**Supplementary Fig.1. Clustered heatmaps of the correlational matrices of the 54 monocytic DEGs, the thicknesses of the 34 brain cortical regions and the MCCB subscores in HCs and FES patients.** Partial correlational analyses of the 54 monocytic DEGs normalized RNAseq counts, the thicknesses of the 34 cortical regions and the MCCB subscores, controlled for age, sex, and education years in HCs **(A)** and FES patients **(B)**, respectively. Heatmap colors represent the negative (blue) and positive (red) partial correlation coefficient, respectively. Monocyte subset-related DEGs, cortical regions and MCCB test modules are categorized with respective color bars.

## Supplementary Figure 2.

**A**


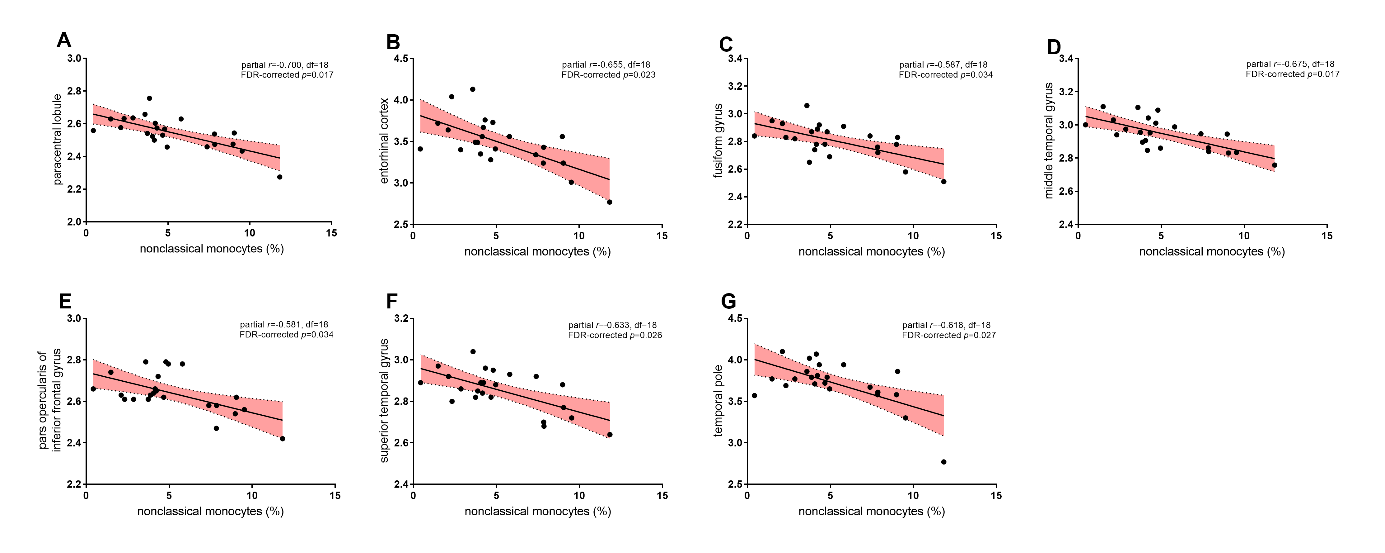


**B**


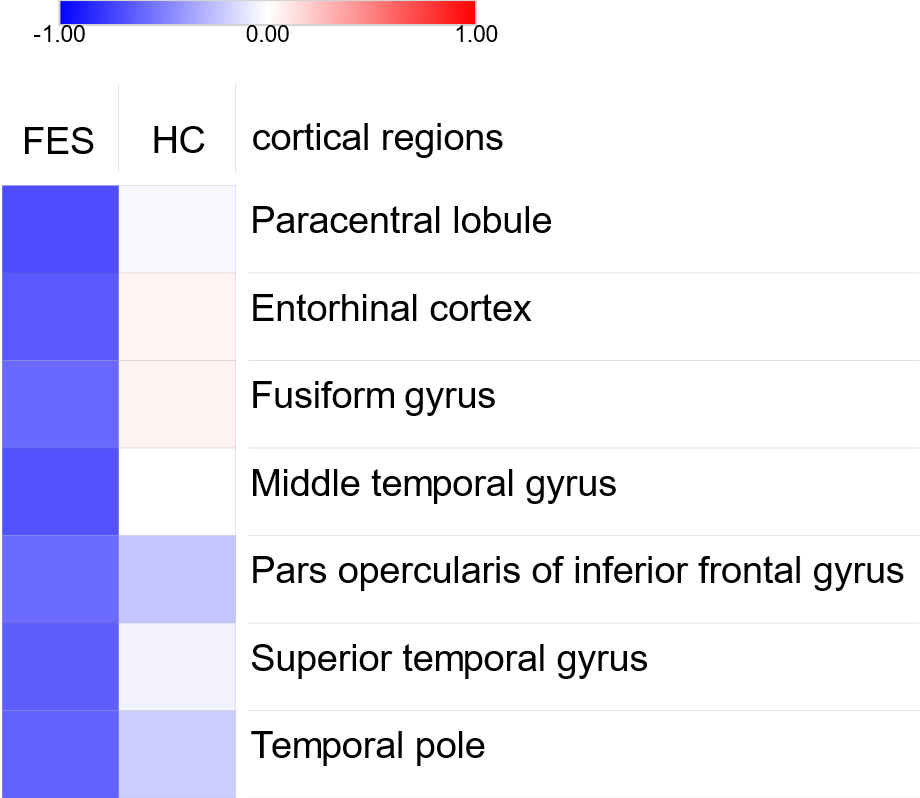


**Supplementary Fig.2. The associations between the percentage of nonclassical monocytes and the thicknesses of cortical regions. (A)** Scatter plots indicated that the percentage of nonclassical monocytes was significantly inversely correlated with the cortical thicknesses of seven anatomic regions in FES group (all FDR < 0.05). partial r and p values were obtained after adjustment for age, sex and education years. Colored regions were 95% confidence bands. **(B)** Heatmap of correlations between the percentage of nonclassical monocytes associated with the seven cortical regions as above mentioned in both FES and HC groups. Color bar represents partial correlation coefficient.
